# Supplementary figures and images for: Distinct elongation stalls during translation are linked with distinct pathways for mRNA degradation
Source: eLife. 2022 Jul 27;11:e76038. doi: 10.7554/eLife.76038 (PMC9352352; doi:10.7554/eLife.76038)

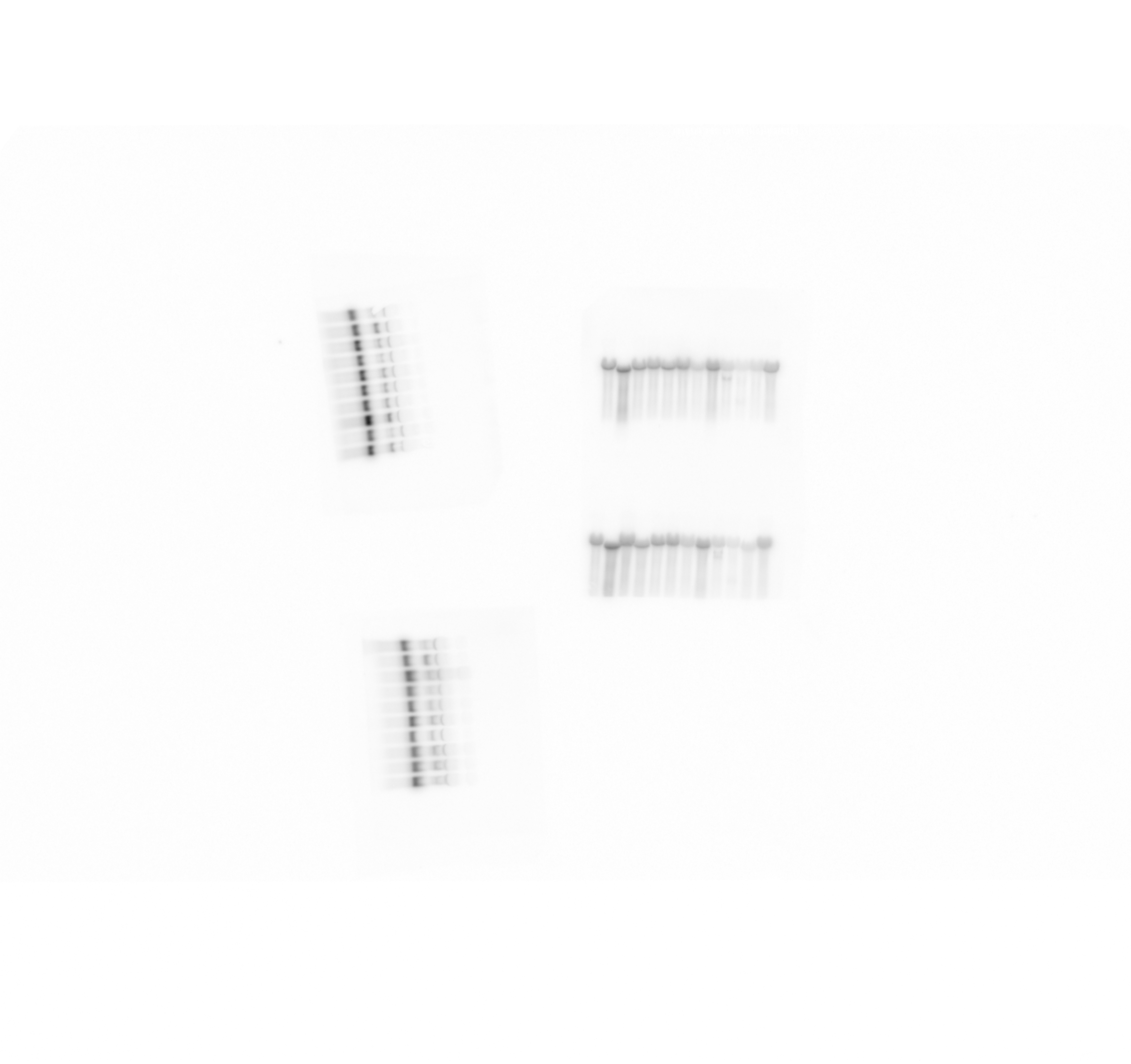

Supplement: Figure 1—source data 1. [file elife-76038-fig1-data1.zip › 1D_northern_blot_images/raw_image_gfp_reps_1-2.gel]

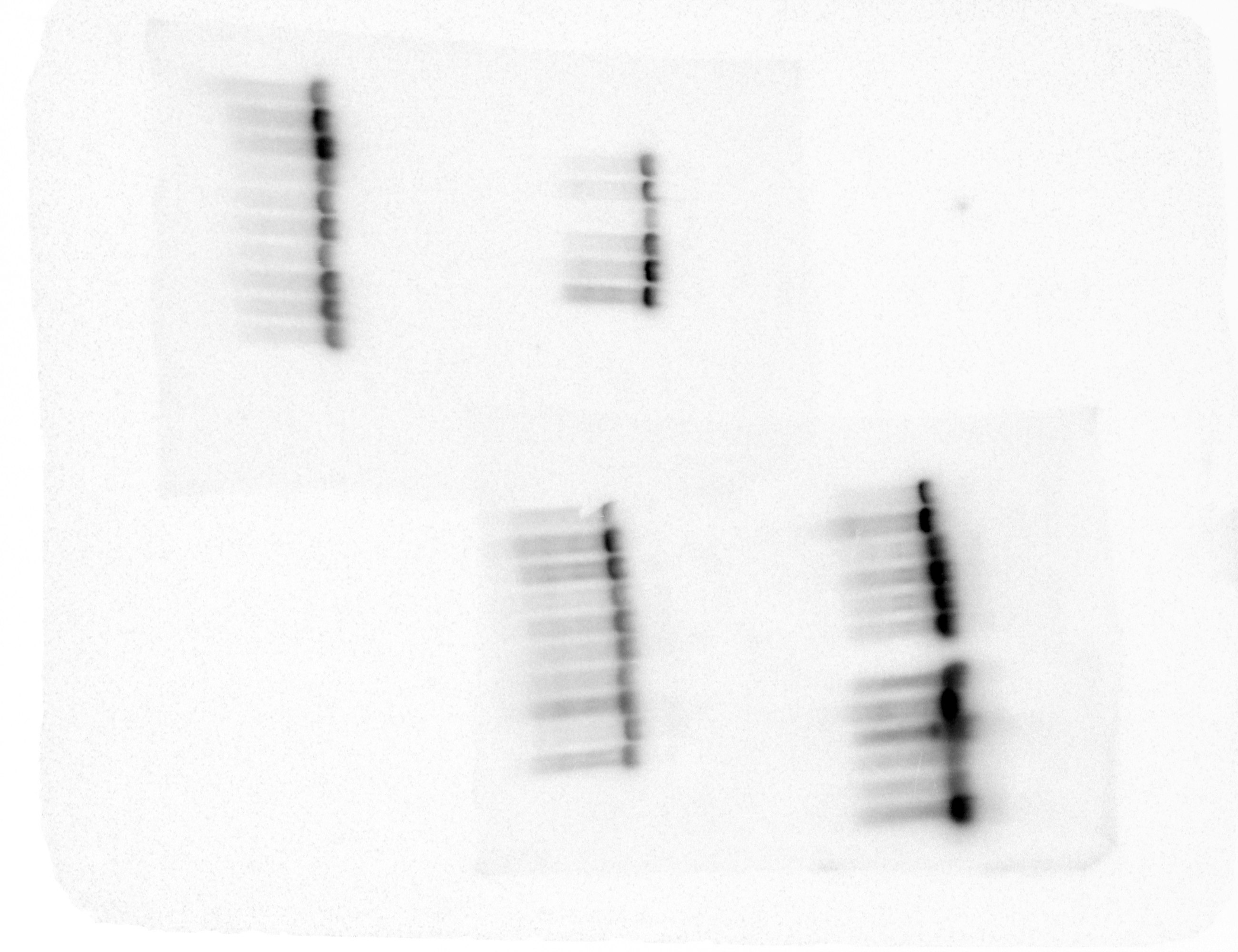

Supplement: Figure 1—source data 1. [file elife-76038-fig1-data1.zip › 1D_northern_blot_images/raw_image_gfp_rep_3.gel]

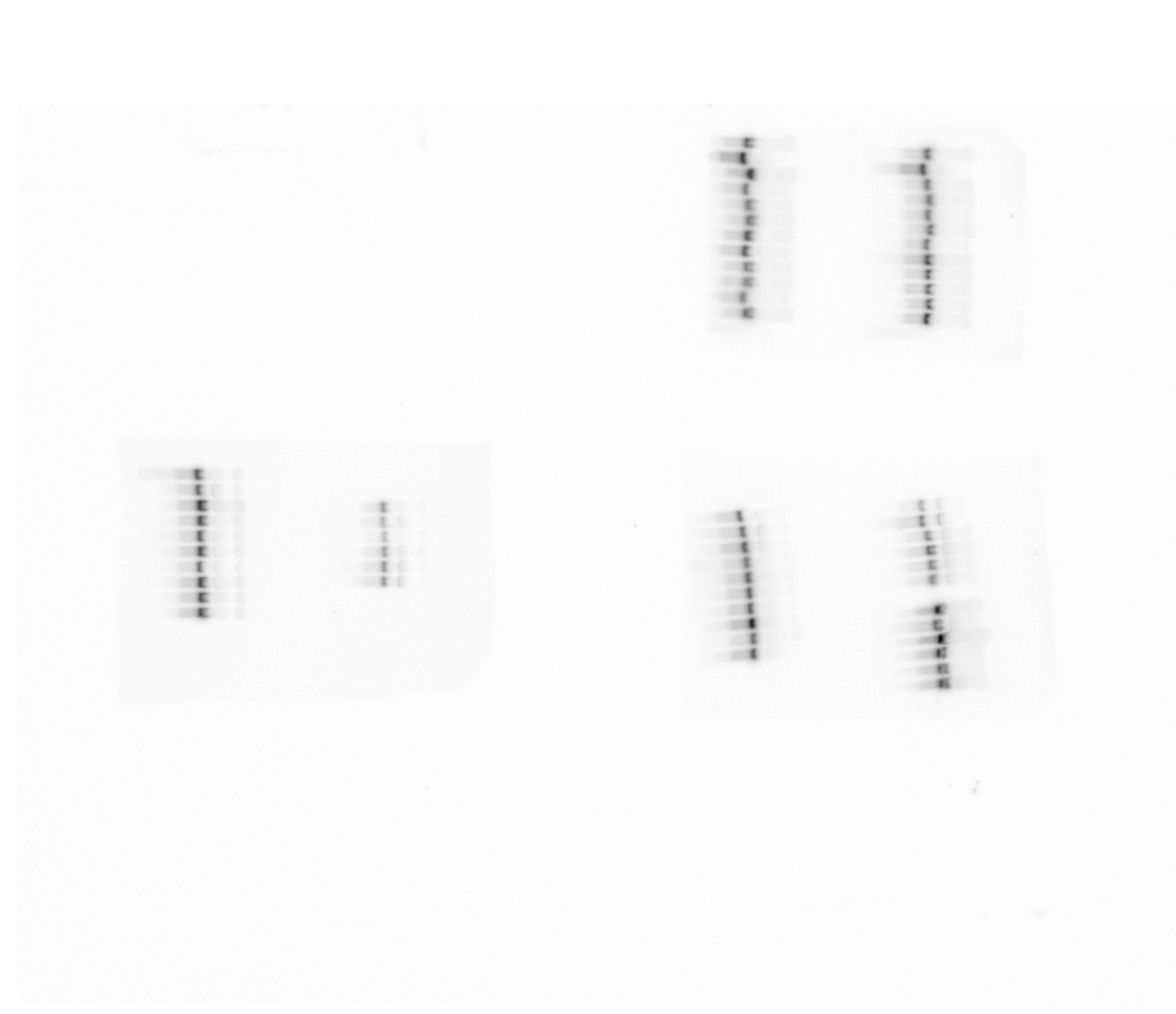

Supplement: Figure 1—source data 1. [file elife-76038-fig1-data1.zip › 1D_northern_blot_images/raw_image_rfp_reps_1-3.gel]

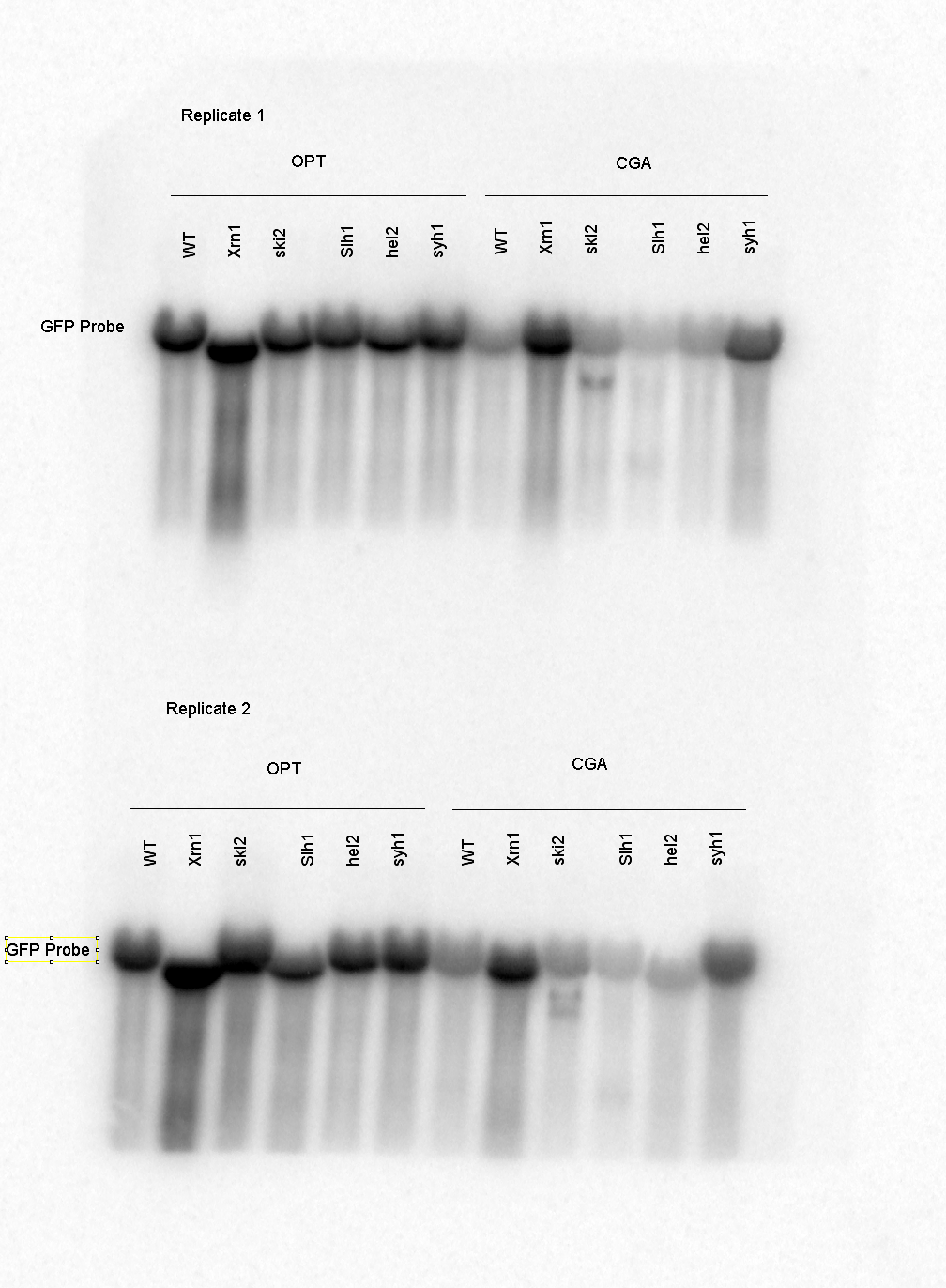

Supplement: Figure 1—source data 1. [file elife-76038-fig1-data1.zip › 1D_northern_blot_images/annotated_gfp_reps_1-2.tif]

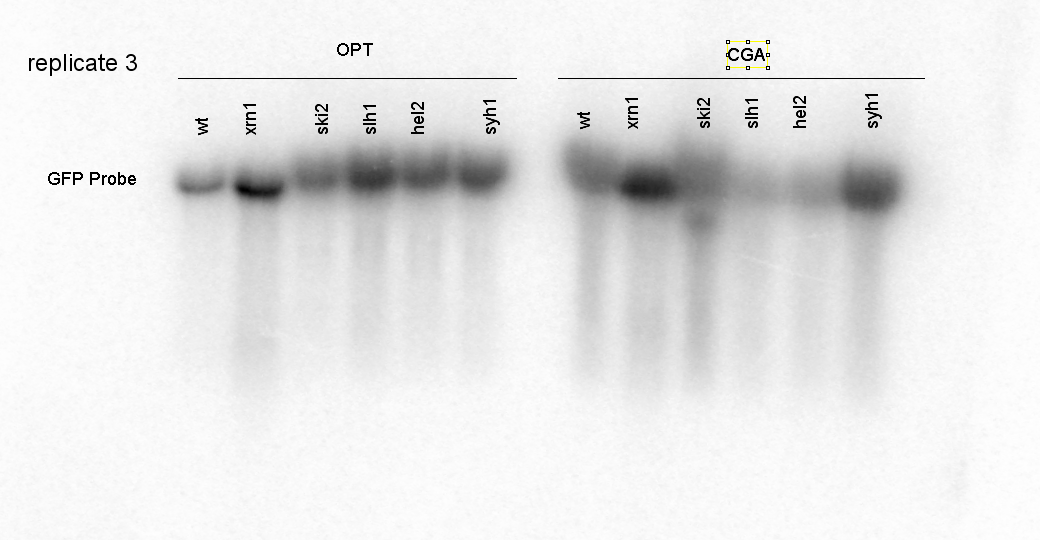

Supplement: Figure 1—source data 1. [file elife-76038-fig1-data1.zip › 1D_northern_blot_images/annotated_gfp_rep_3.tif]

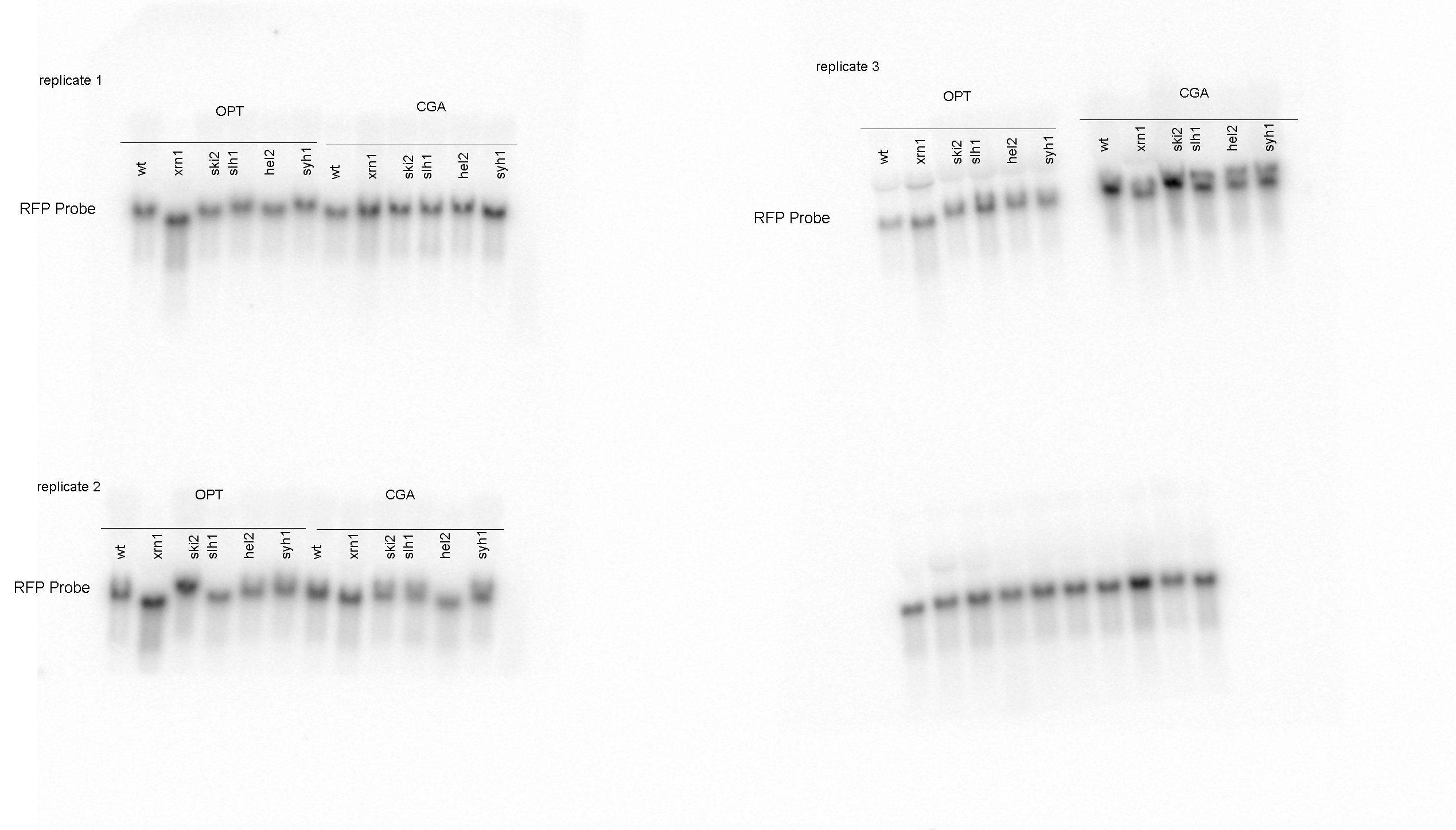

Supplement: Figure 1—source data 1. [file elife-76038-fig1-data1.zip › 1D_northern_blot_images/annotated_rfp_reps_1-3.tif]

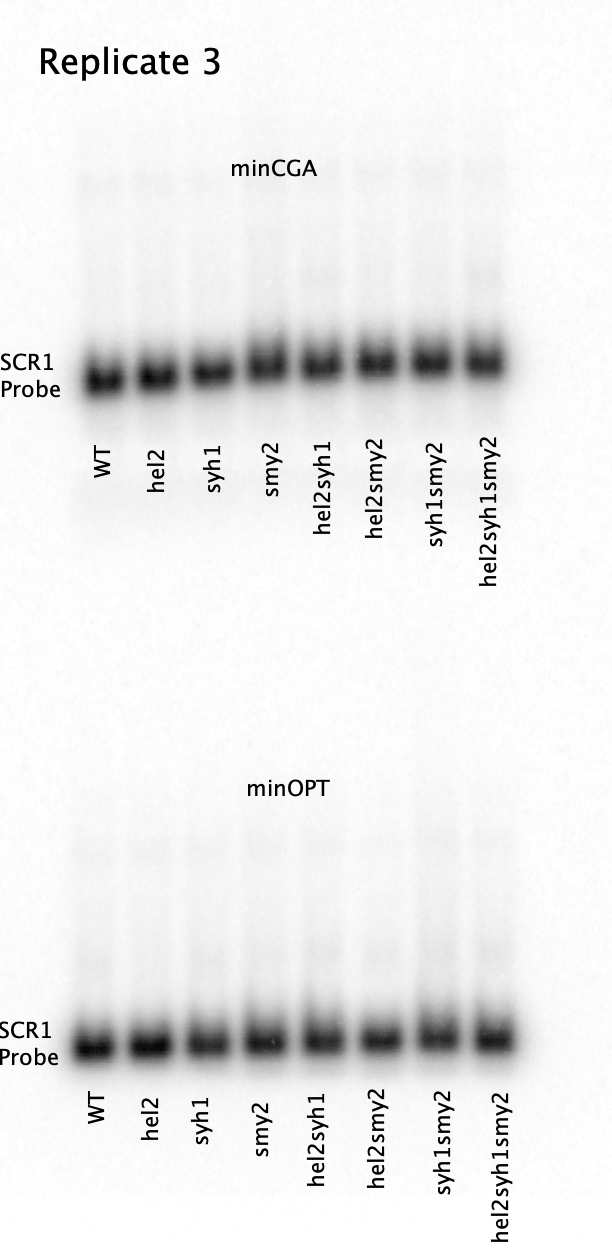

Supplement: Figure 2—source data 1. [file elife-76038-fig2-data1.zip › S2B_northern_blot_images/annotated_scr1_rep_3.tif]

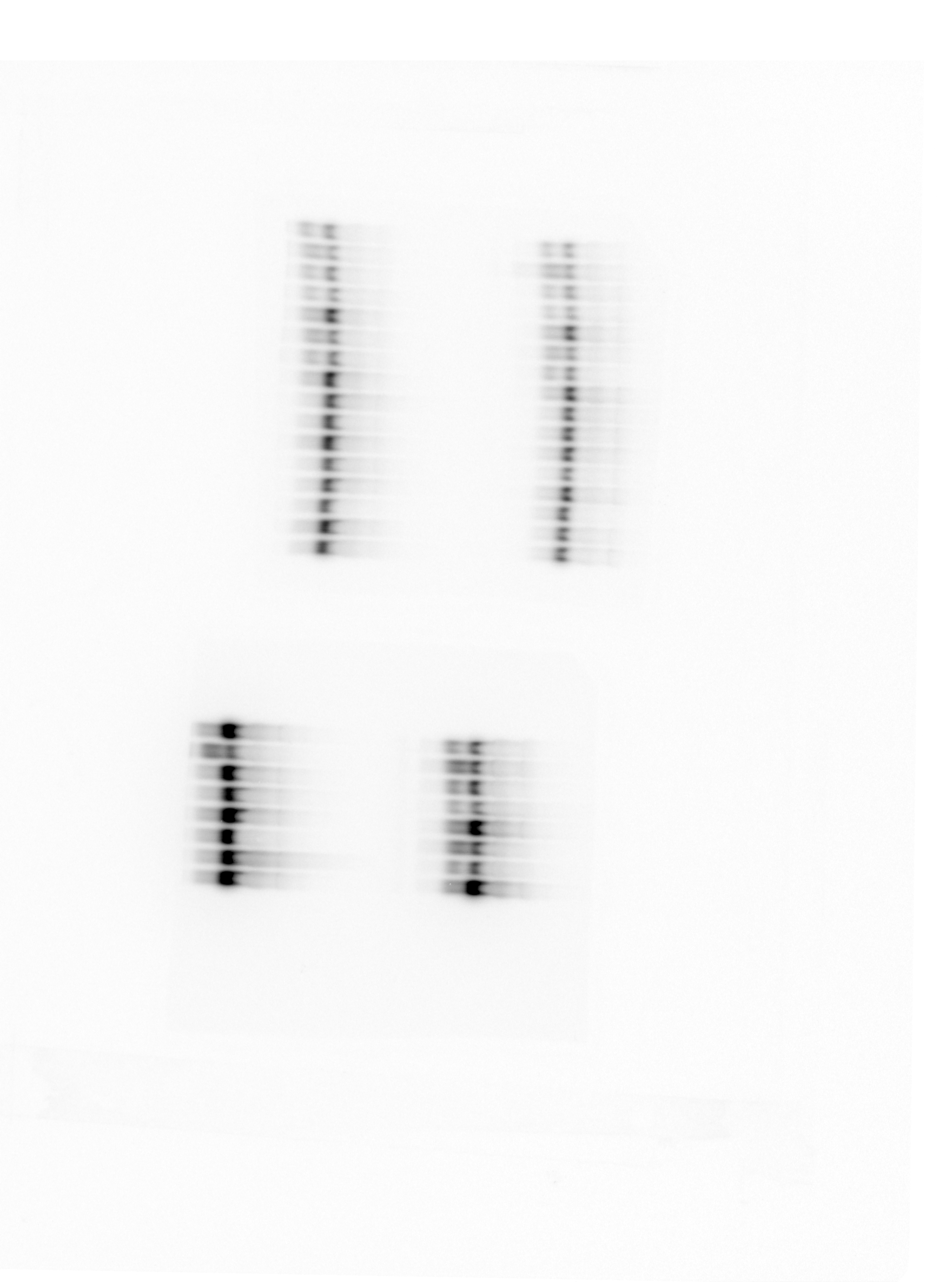

Supplement: Figure 2—source data 1. [file elife-76038-fig2-data1.zip › S2B_northern_blot_images/raw_image_his3_reps_1-3.gel]

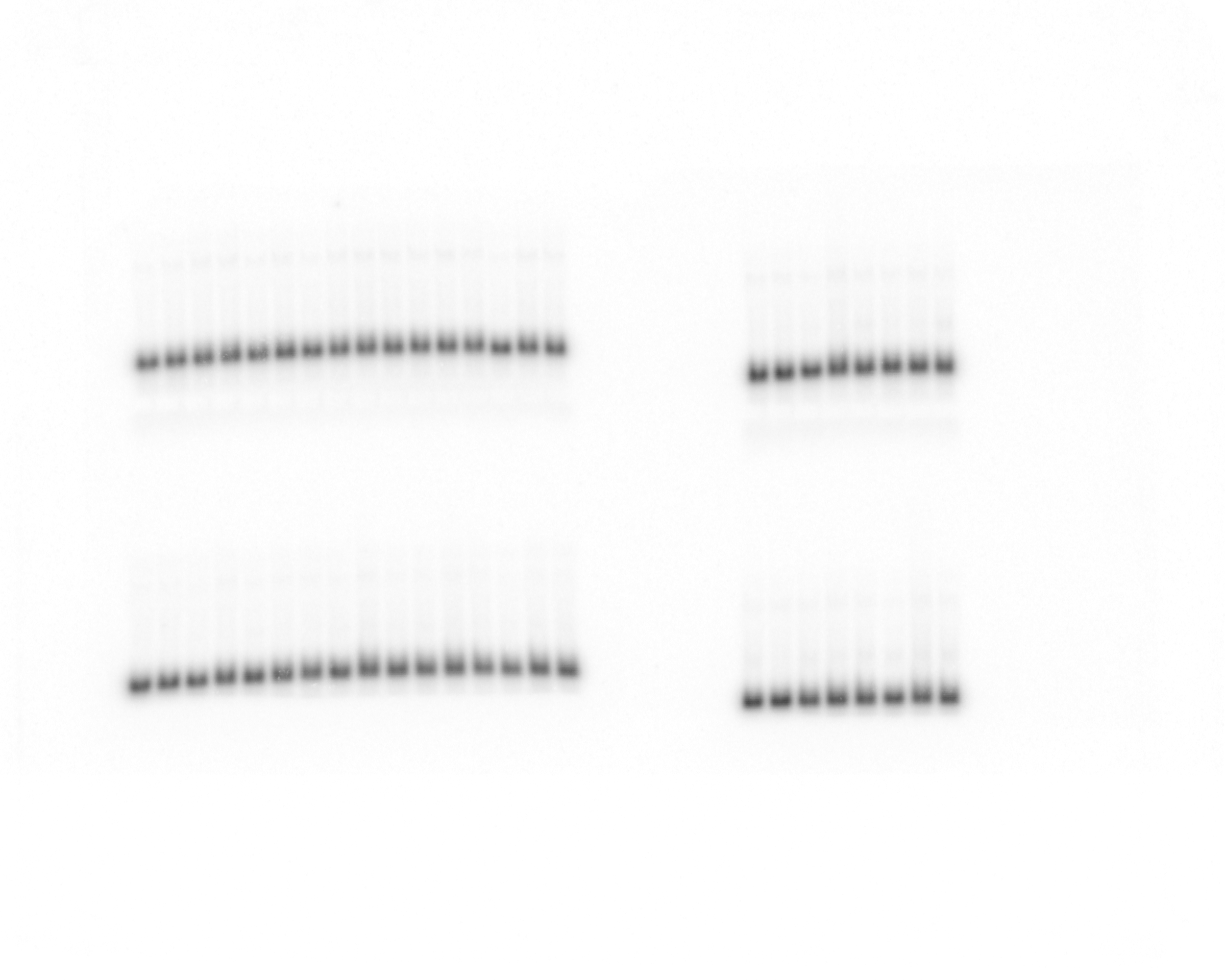

Supplement: Figure 2—source data 1. [file elife-76038-fig2-data1.zip › S2B_northern_blot_images/raw_image_scr1_reps_1-3.gel]

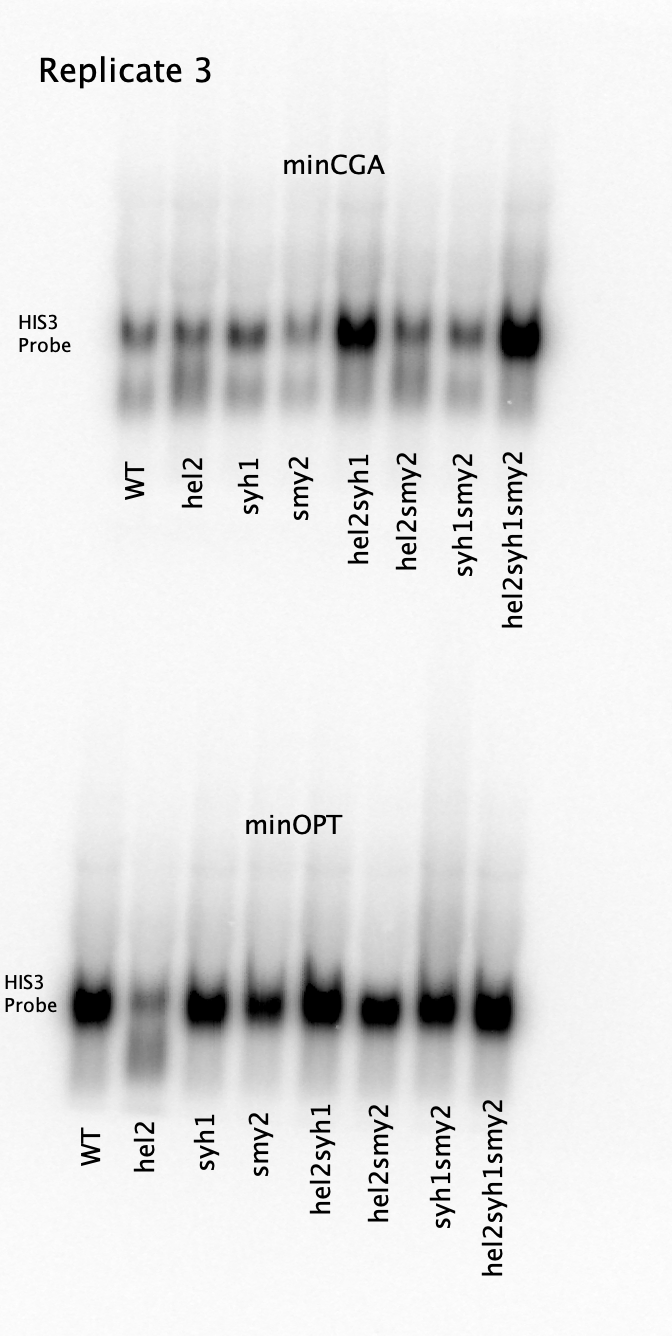

Supplement: Figure 2—source data 1. [file elife-76038-fig2-data1.zip › S2B_northern_blot_images/annotated_his3_rep_3.tif]

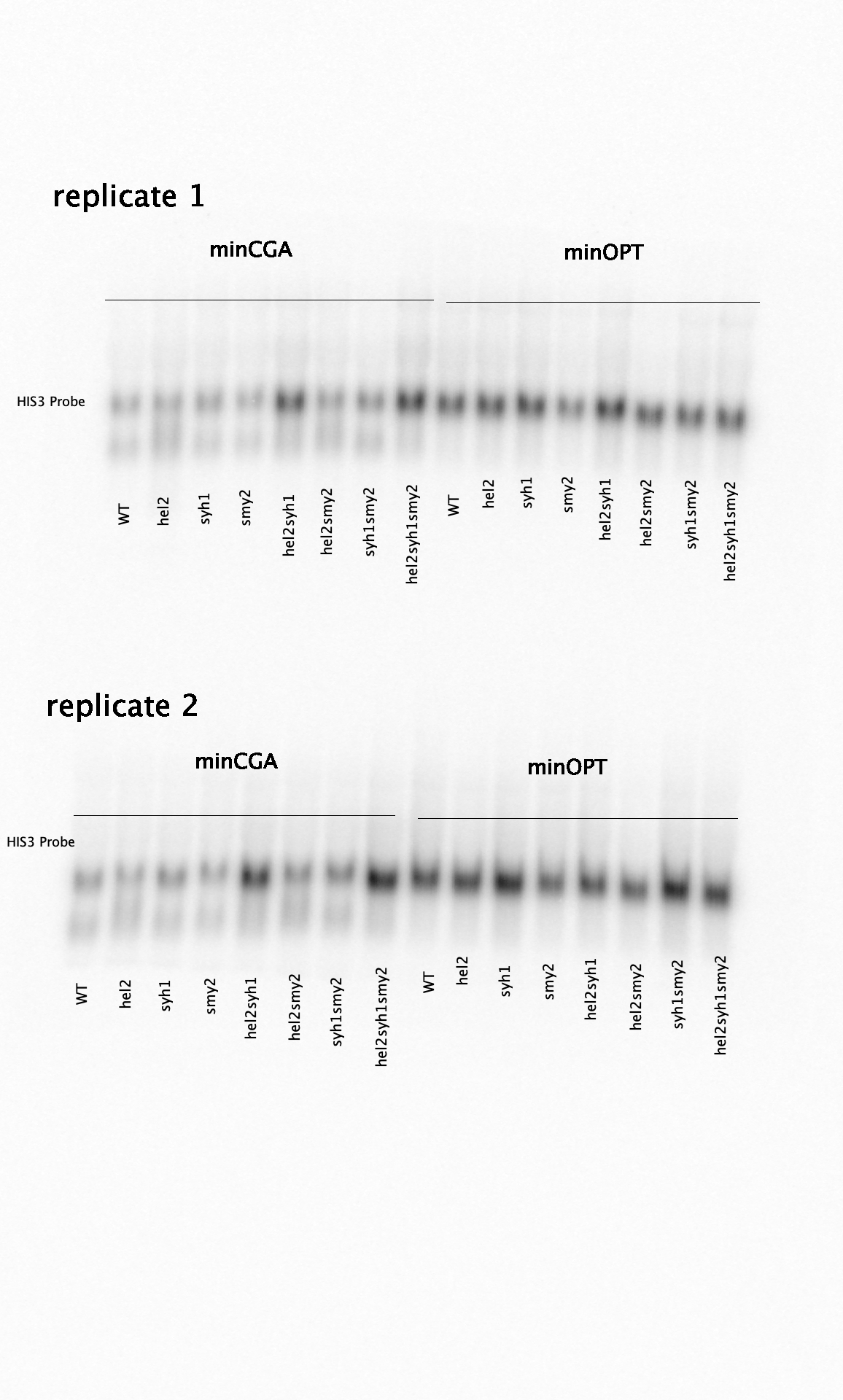

Supplement: Figure 2—source data 1. [file elife-76038-fig2-data1.zip › S2B_northern_blot_images/annotated_his3_reps_1-2.tif]

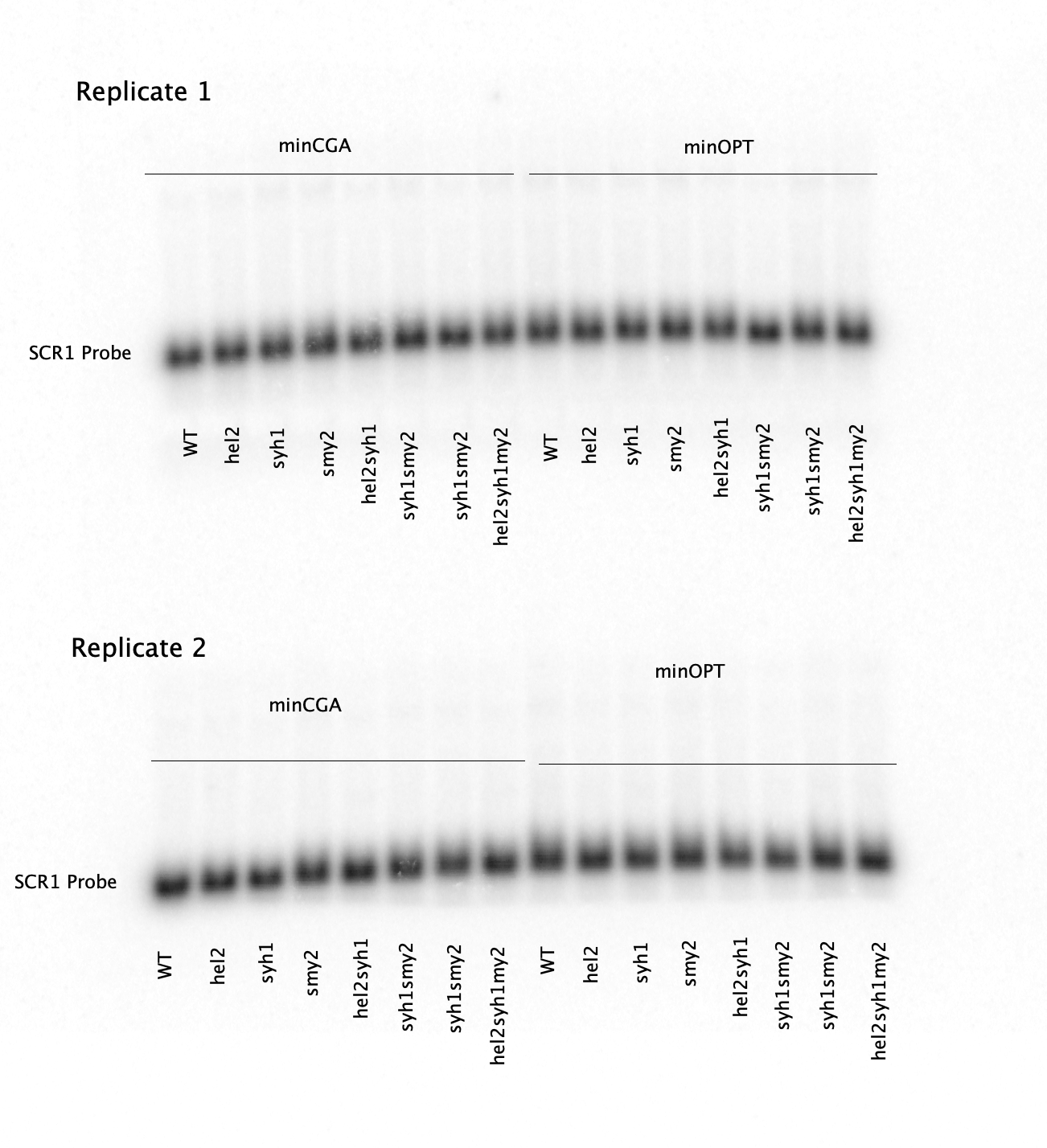

Supplement: Figure 2—source data 1. [file elife-76038-fig2-data1.zip › S2B_northern_blot_images/annotated_scr1_reps_1-2.tif]

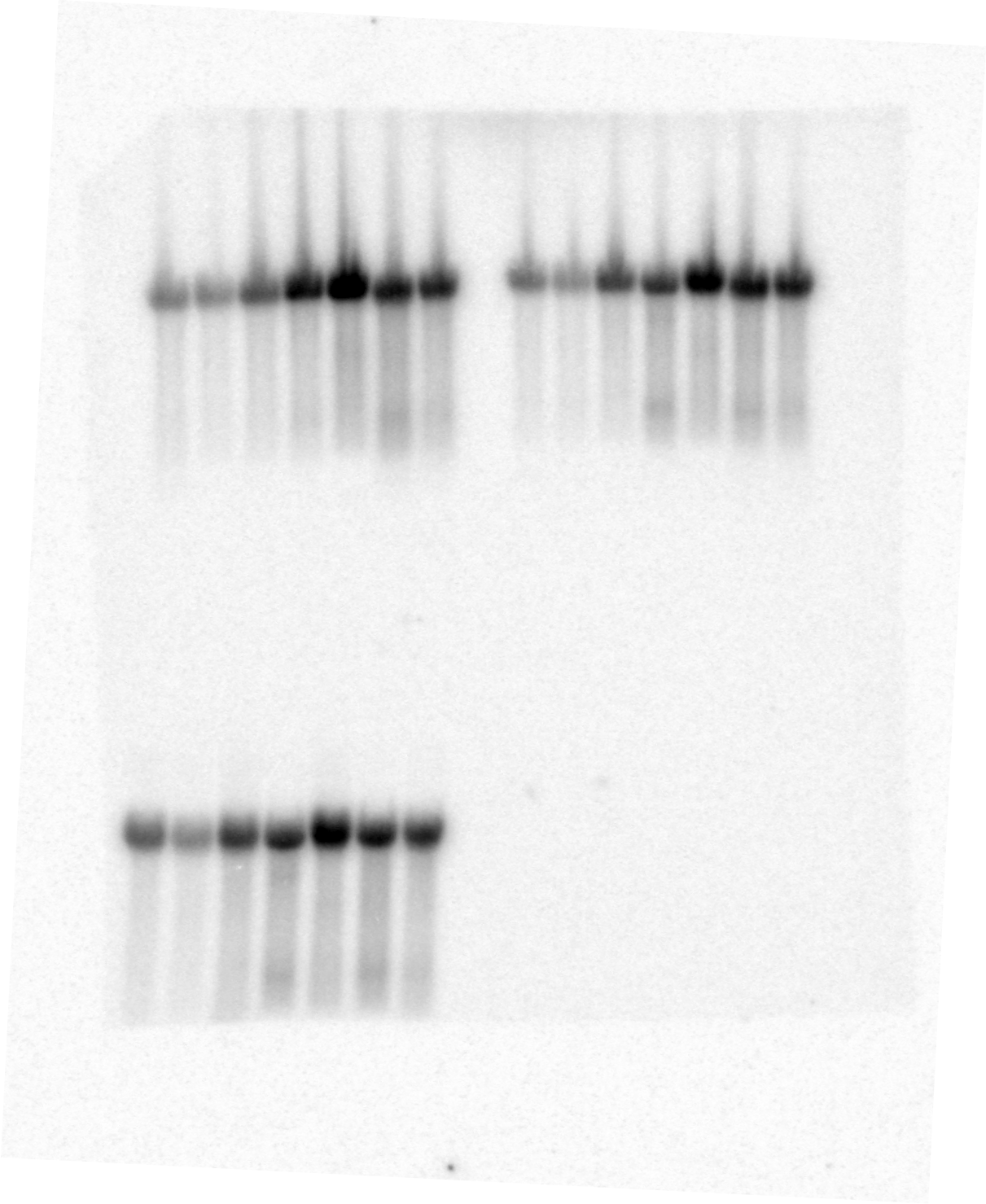

Supplement: Figure 2—source data 1. [file elife-76038-fig2-data1.zip › S2C_northern_blot_images/raw_image_gfp_reps_1-3.gel]

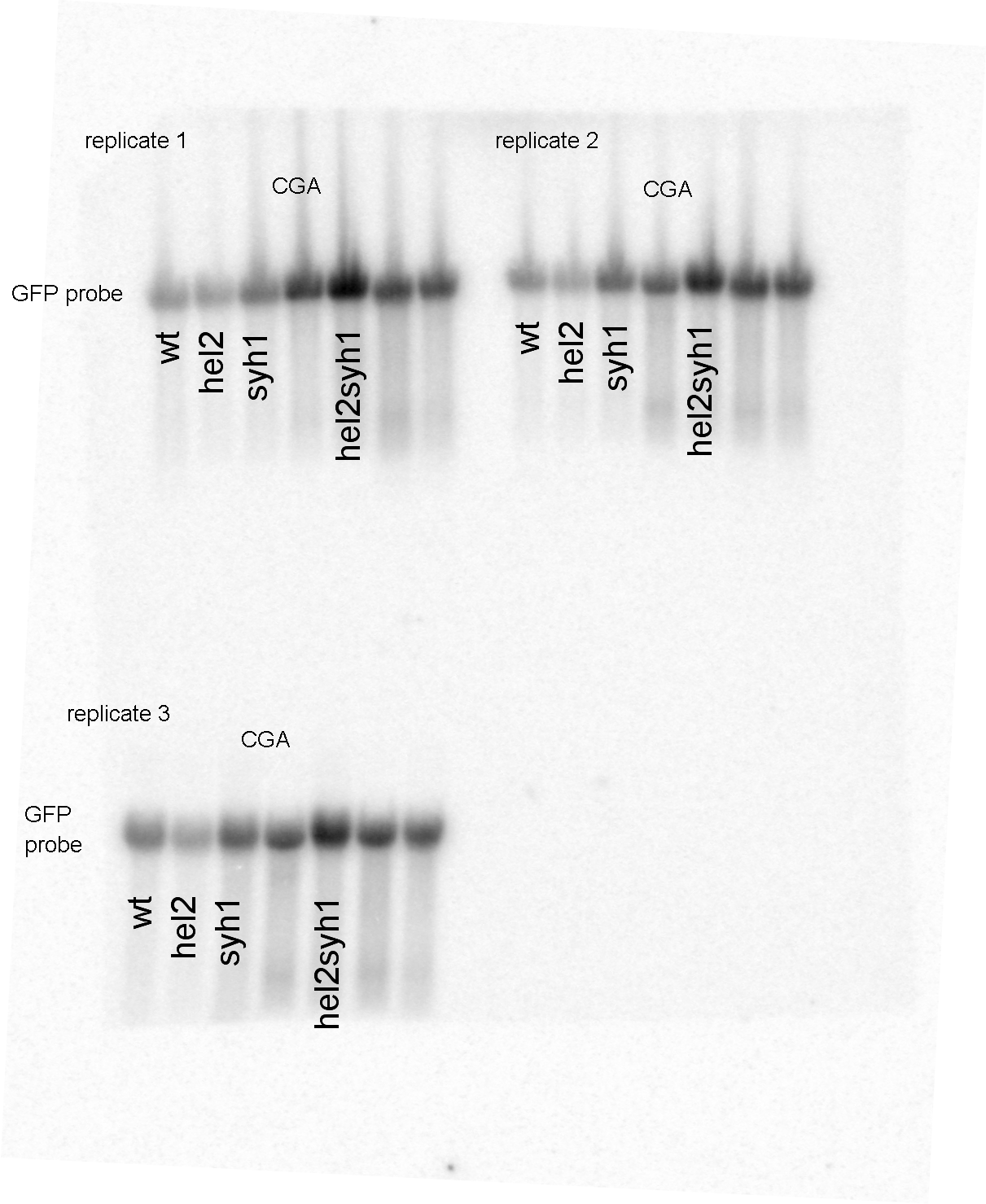

Supplement: Figure 2—source data 1. [file elife-76038-fig2-data1.zip › S2C_northern_blot_images/annotated_gfp_reps_1-3.tif]

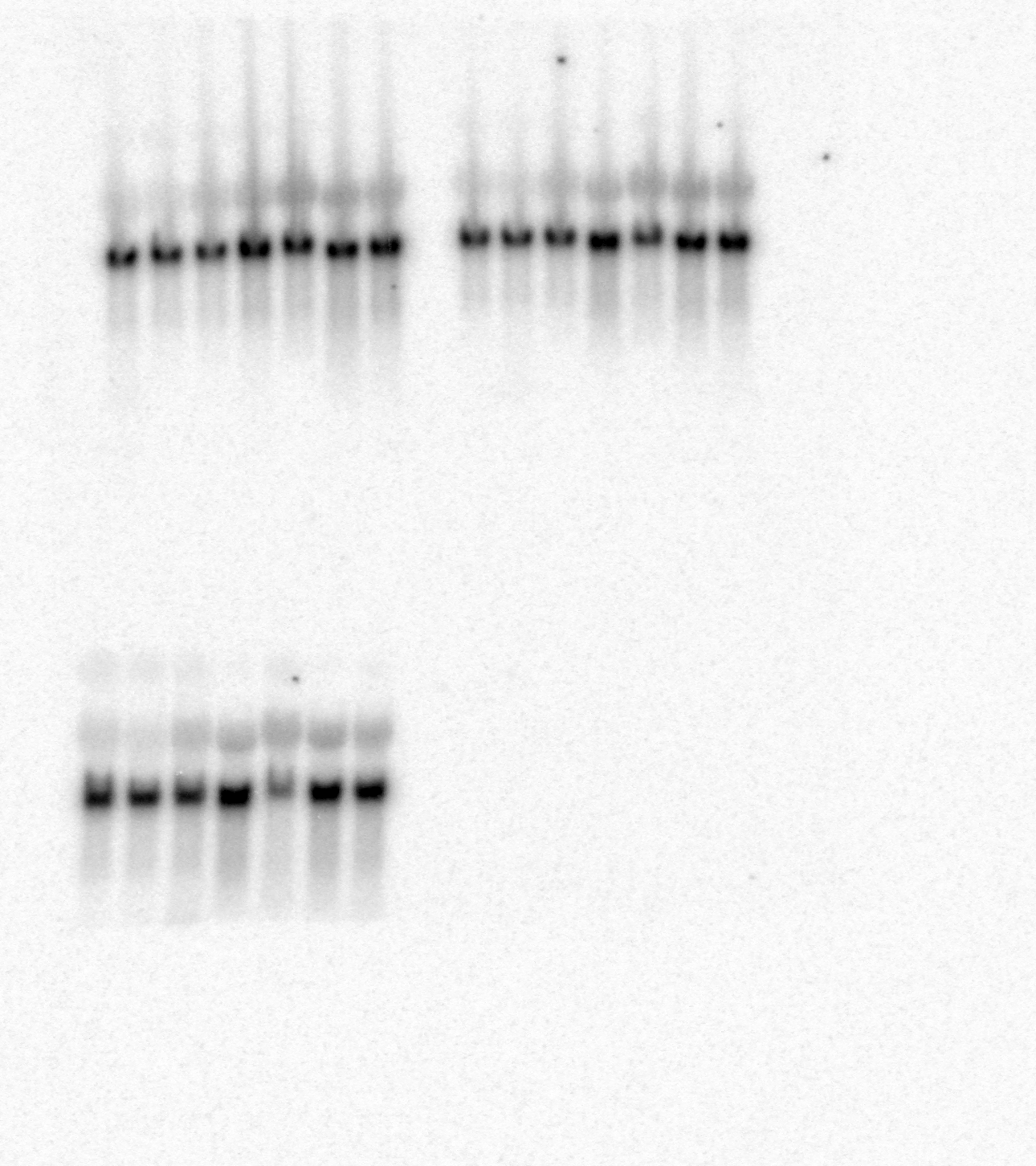

Supplement: Figure 2—source data 1. [file elife-76038-fig2-data1.zip › S2C_northern_blot_images/raw_image_rfp_reps_1-3.gel]

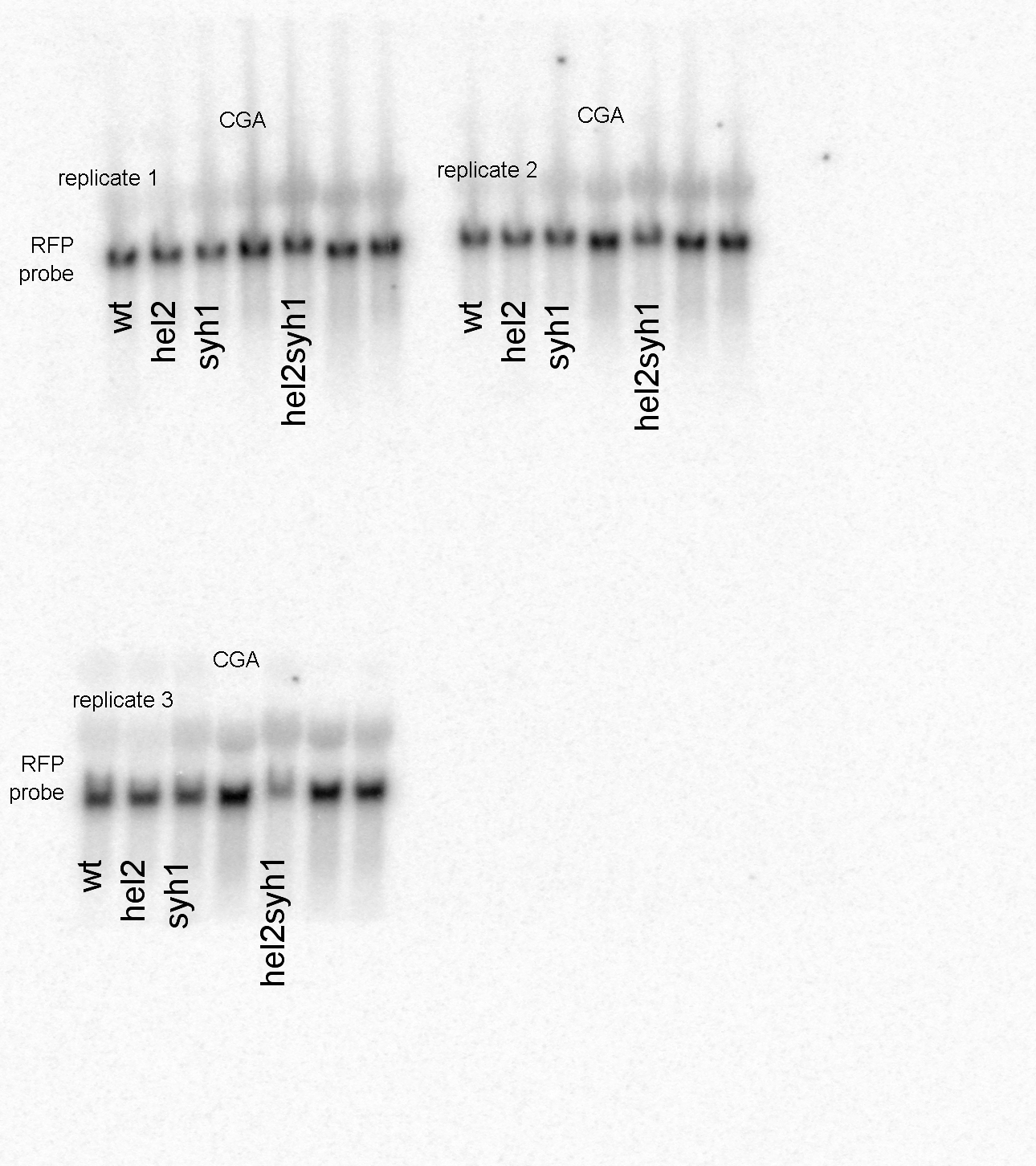

Supplement: Figure 2—source data 1. [file elife-76038-fig2-data1.zip › S2C_northern_blot_images/annotated_rfp_reps_1-3.tif]

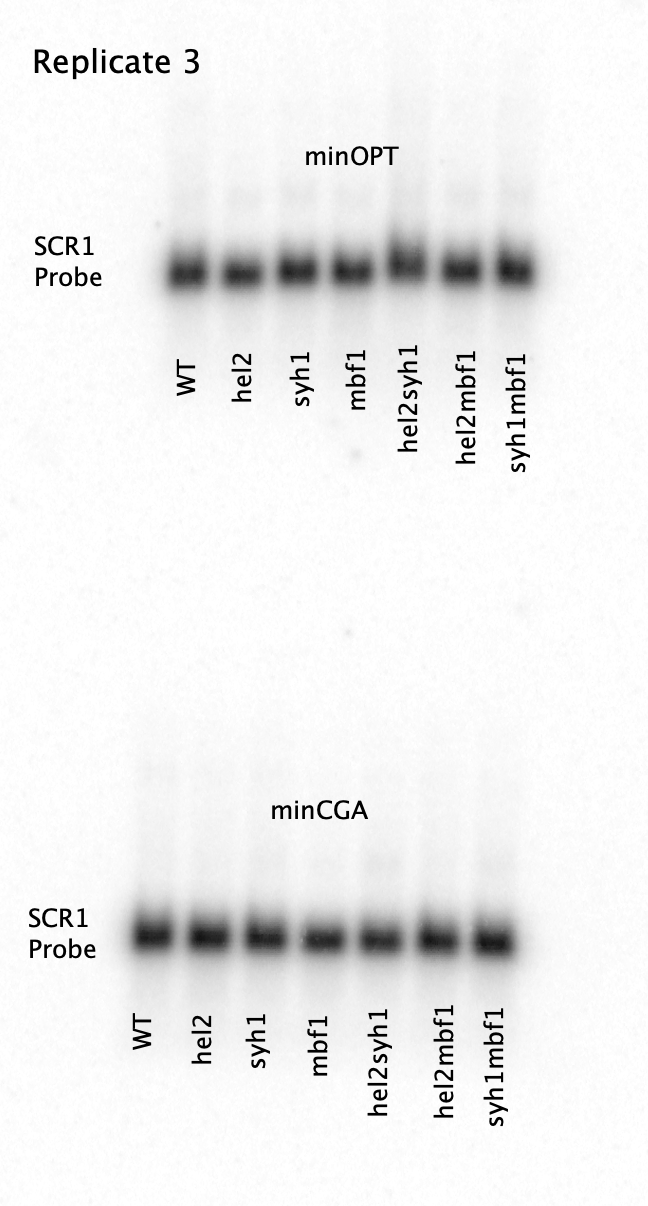

Supplement: Figure 2—source data 1. [file elife-76038-fig2-data1.zip › S2E_northern_blot_images/annotated_scr1_rep_3.tif]

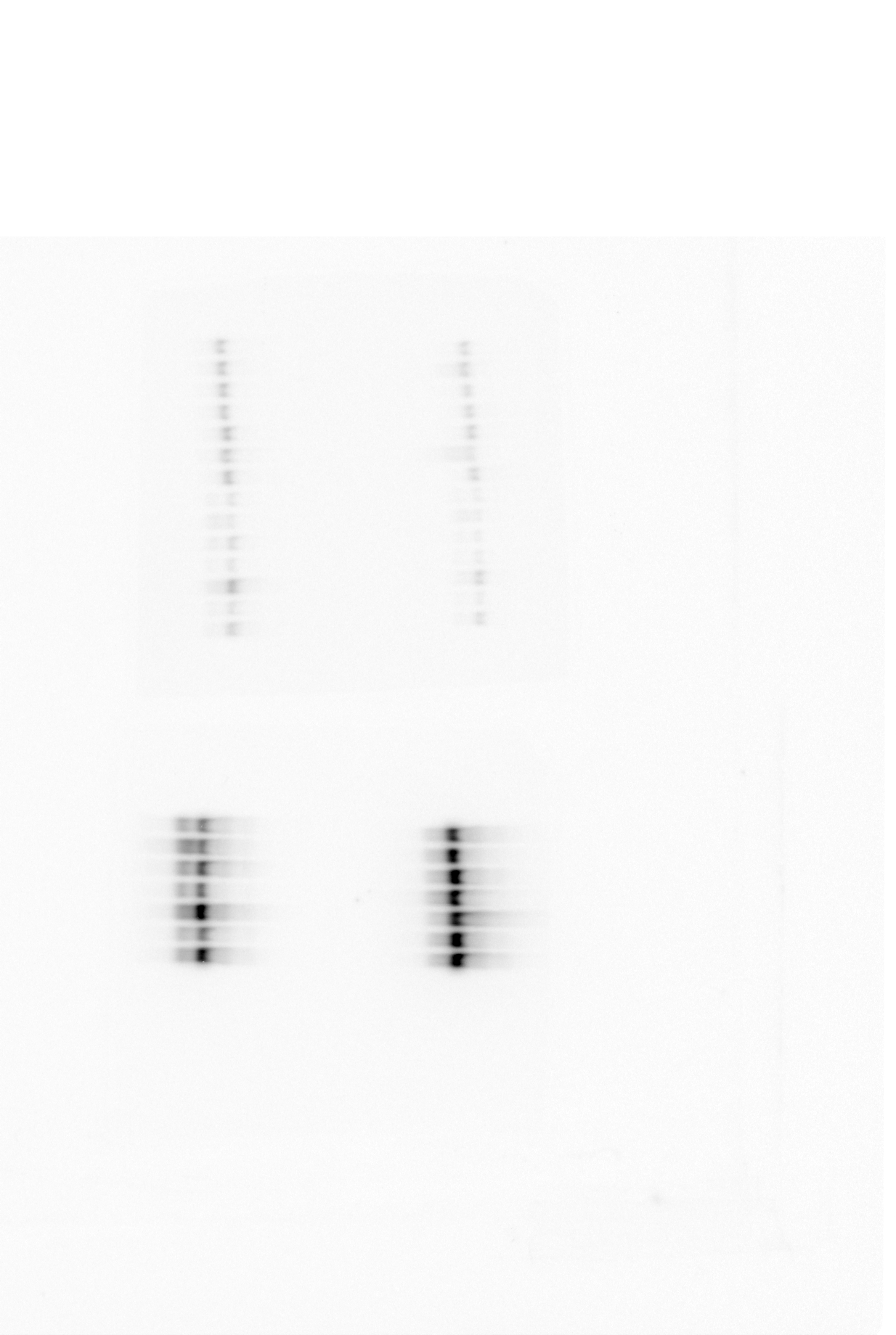

Supplement: Figure 2—source data 1. [file elife-76038-fig2-data1.zip › S2E_northern_blot_images/raw_image_his3_reps_1-3.gel]

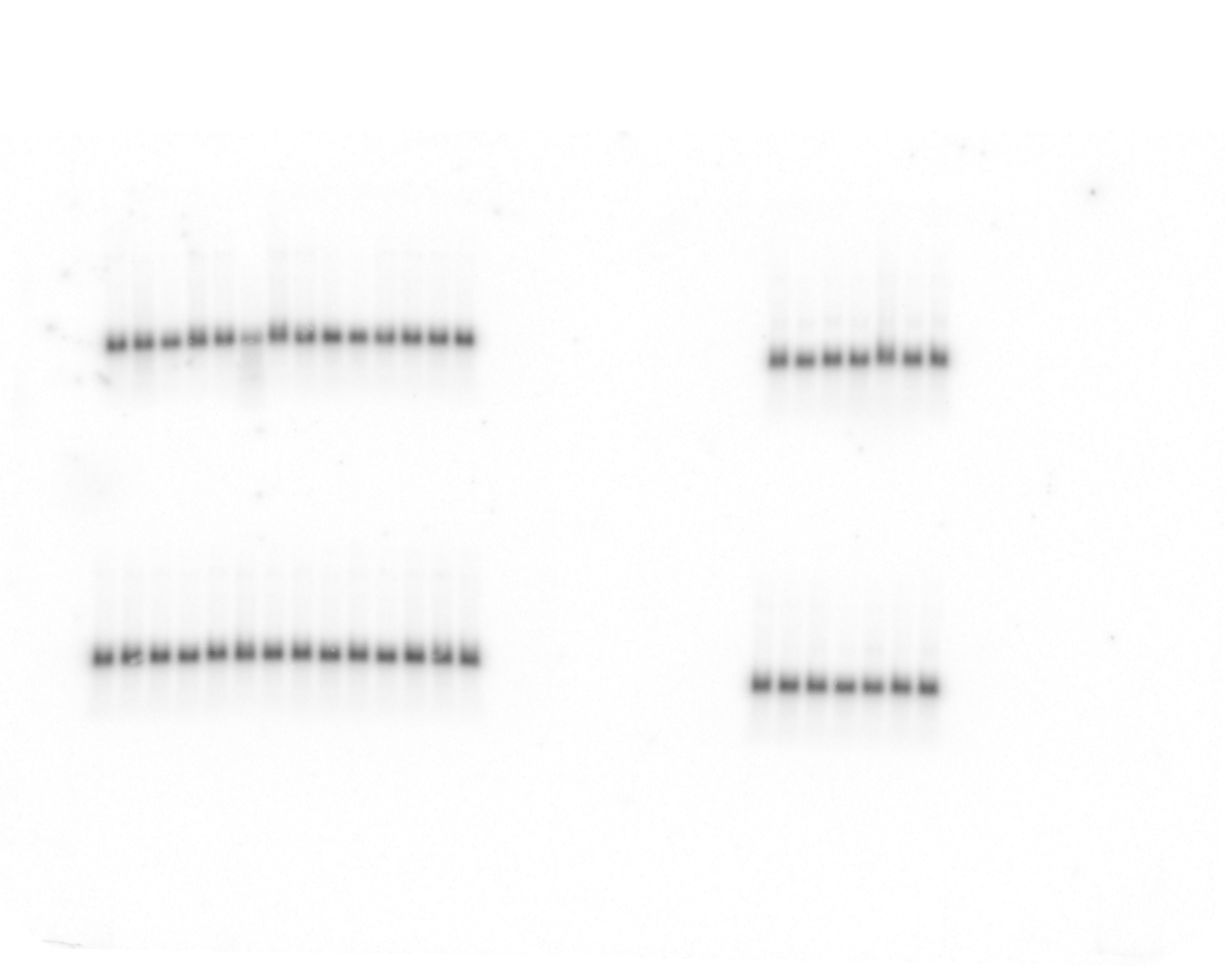

Supplement: Figure 2—source data 1. [file elife-76038-fig2-data1.zip › S2E_northern_blot_images/raw_image_scr1_reps_1-3.gel]

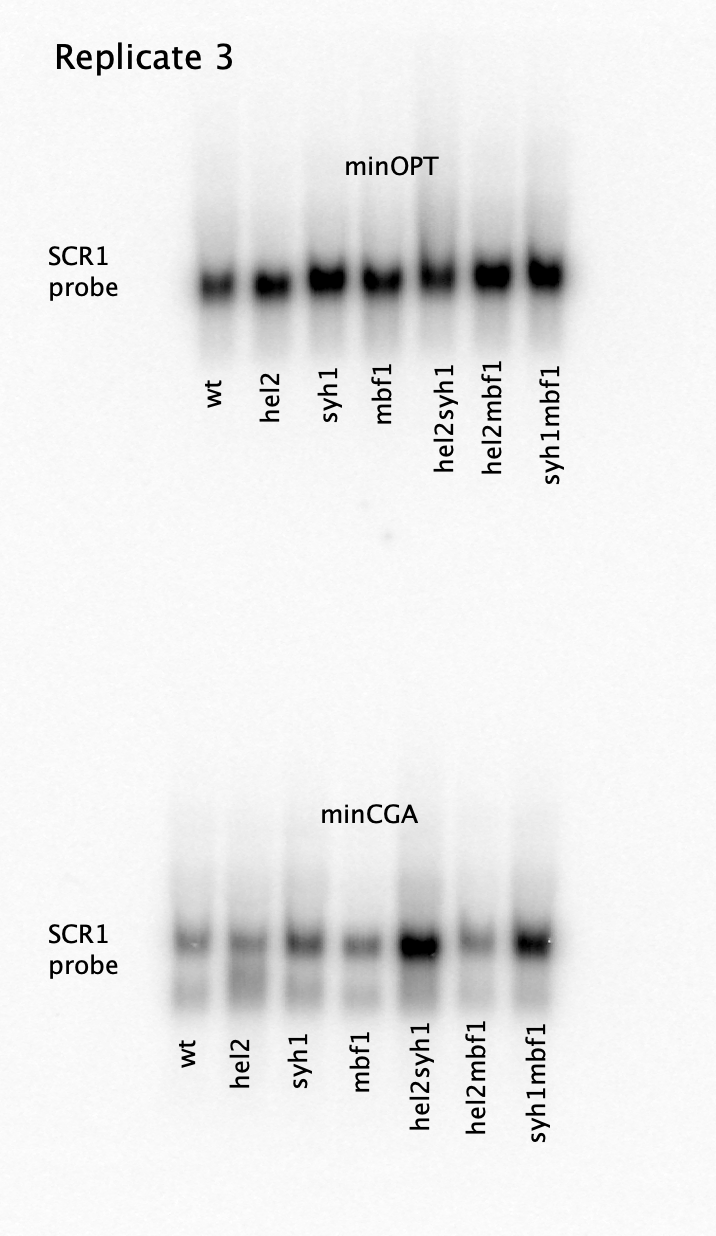

Supplement: Figure 2—source data 1. [file elife-76038-fig2-data1.zip › S2E_northern_blot_images/annotated_his3_rep_3.tif]

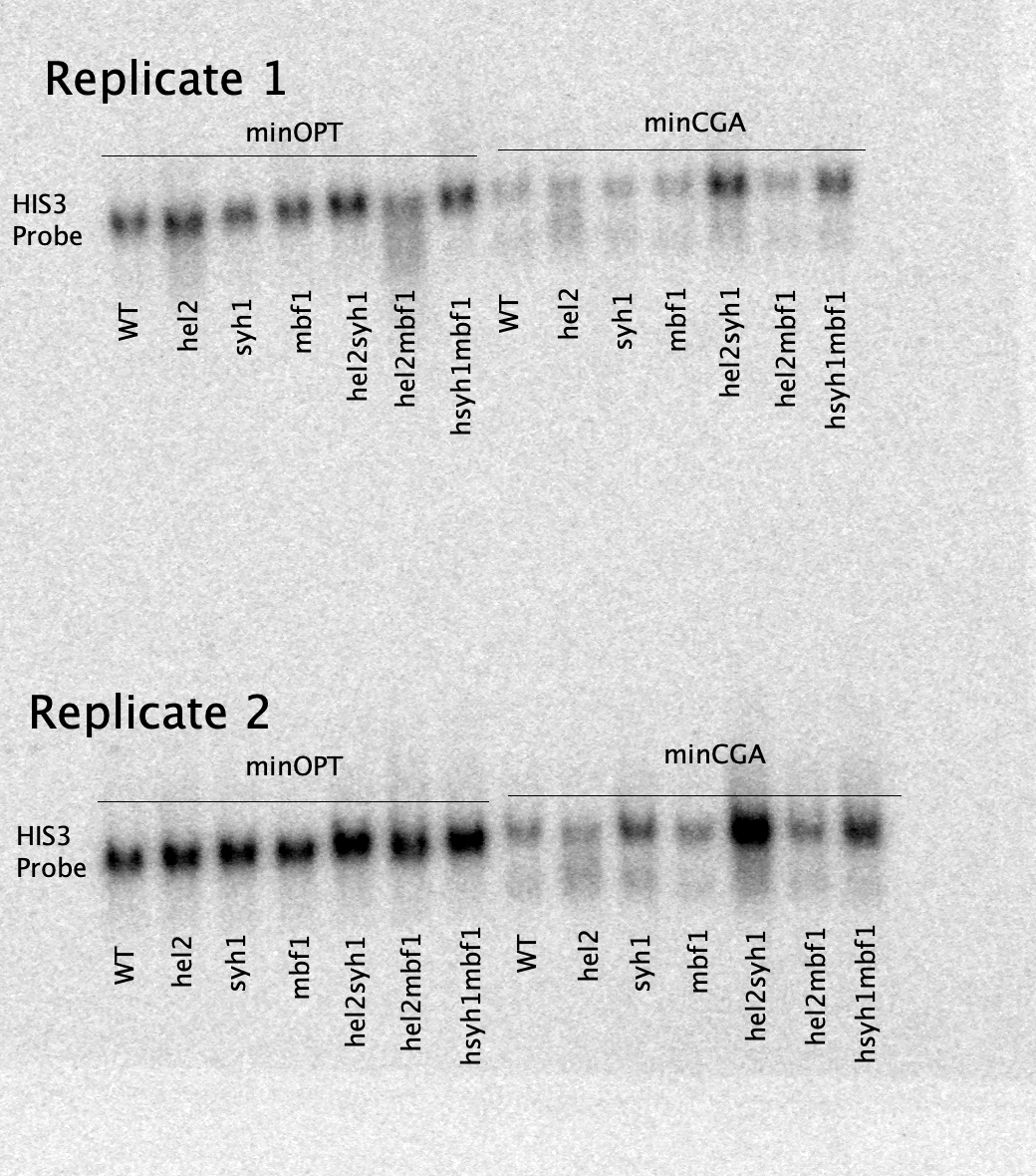

Supplement: Figure 2—source data 1. [file elife-76038-fig2-data1.zip › S2E_northern_blot_images/annotated_his3_reps_1-2.tif]

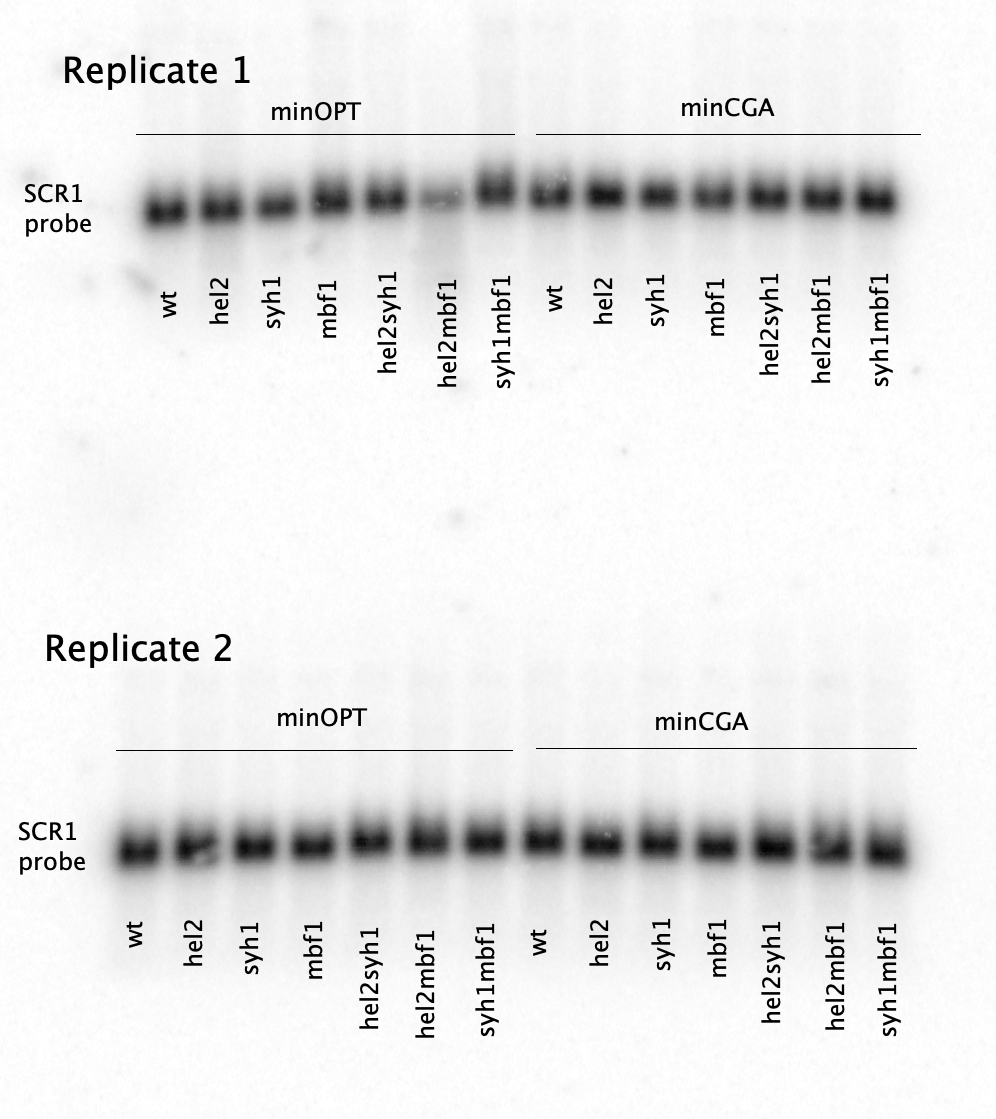

Supplement: Figure 2—source data 1. [file elife-76038-fig2-data1.zip › S2E_northern_blot_images/annotated_scr1_reps_1-2.tif]

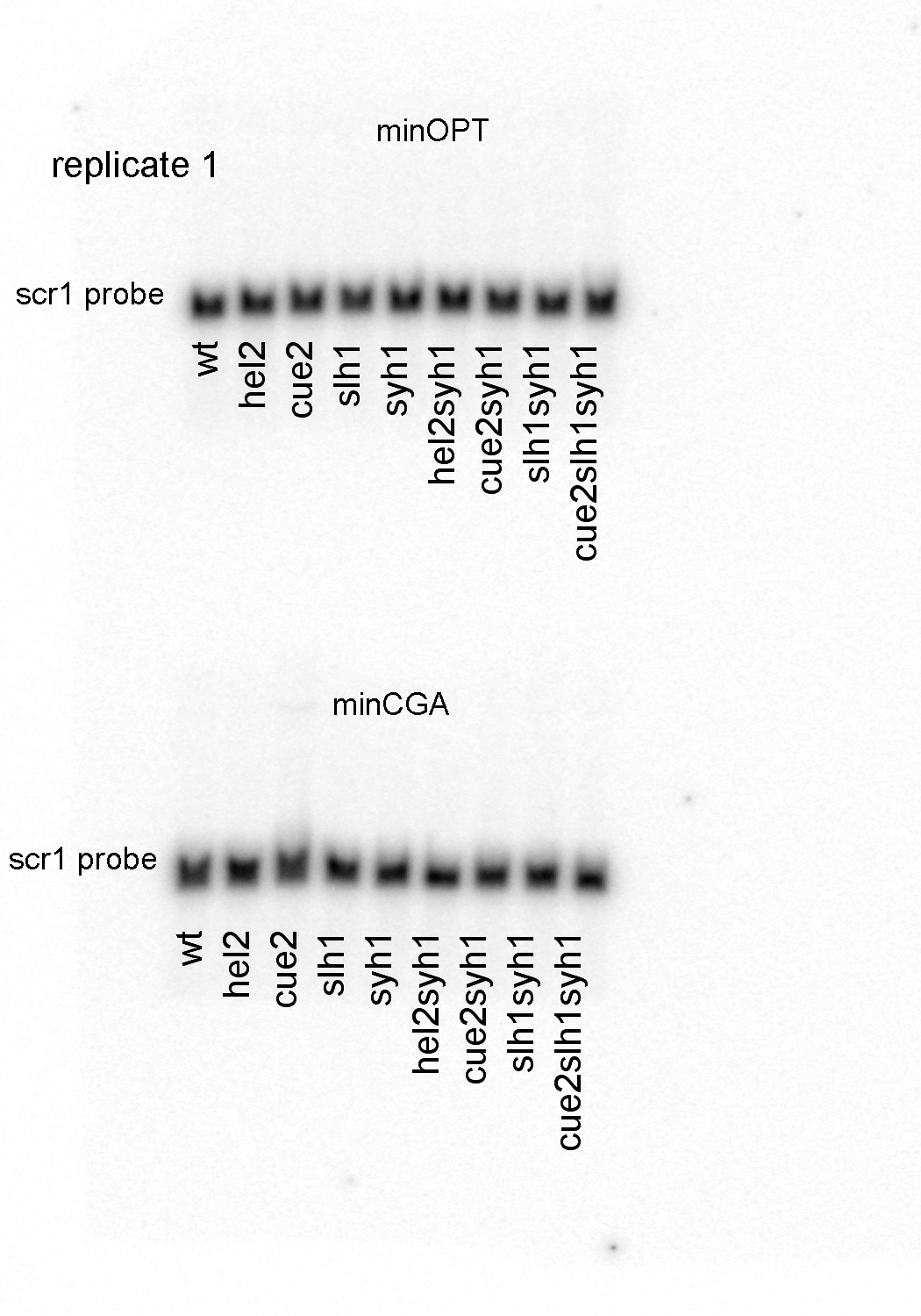

Supplement: Figure 2—source data 2. [file elife-76038-fig2-data2.zip › 2B-C_S2A_northen_blot_images/annotated_scr1_rep_1.tif]

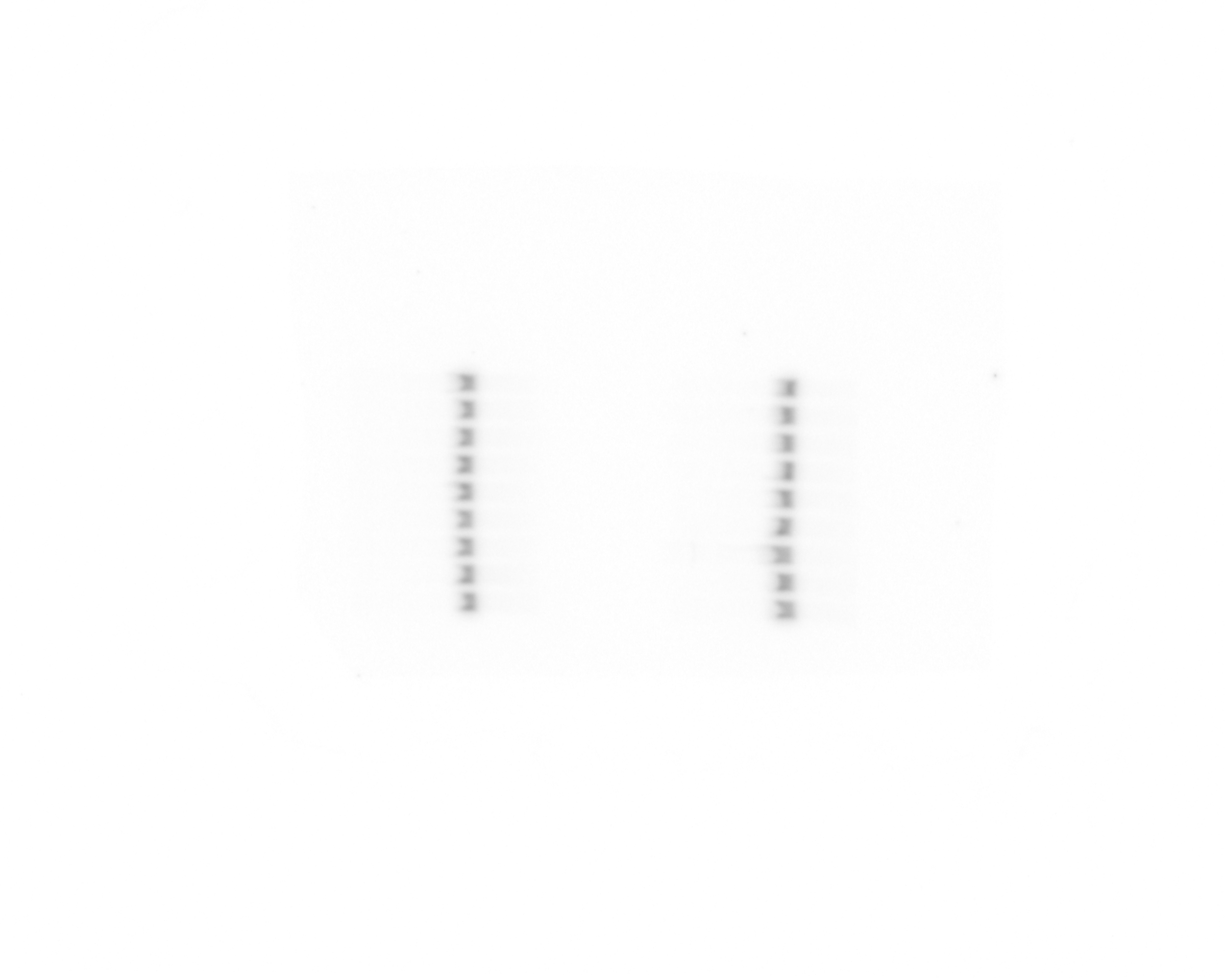

Supplement: Figure 2—source data 2. [file elife-76038-fig2-data2.zip › 2B-C_S2A_northen_blot_images/raw_image_scr1_rep_1.gel]

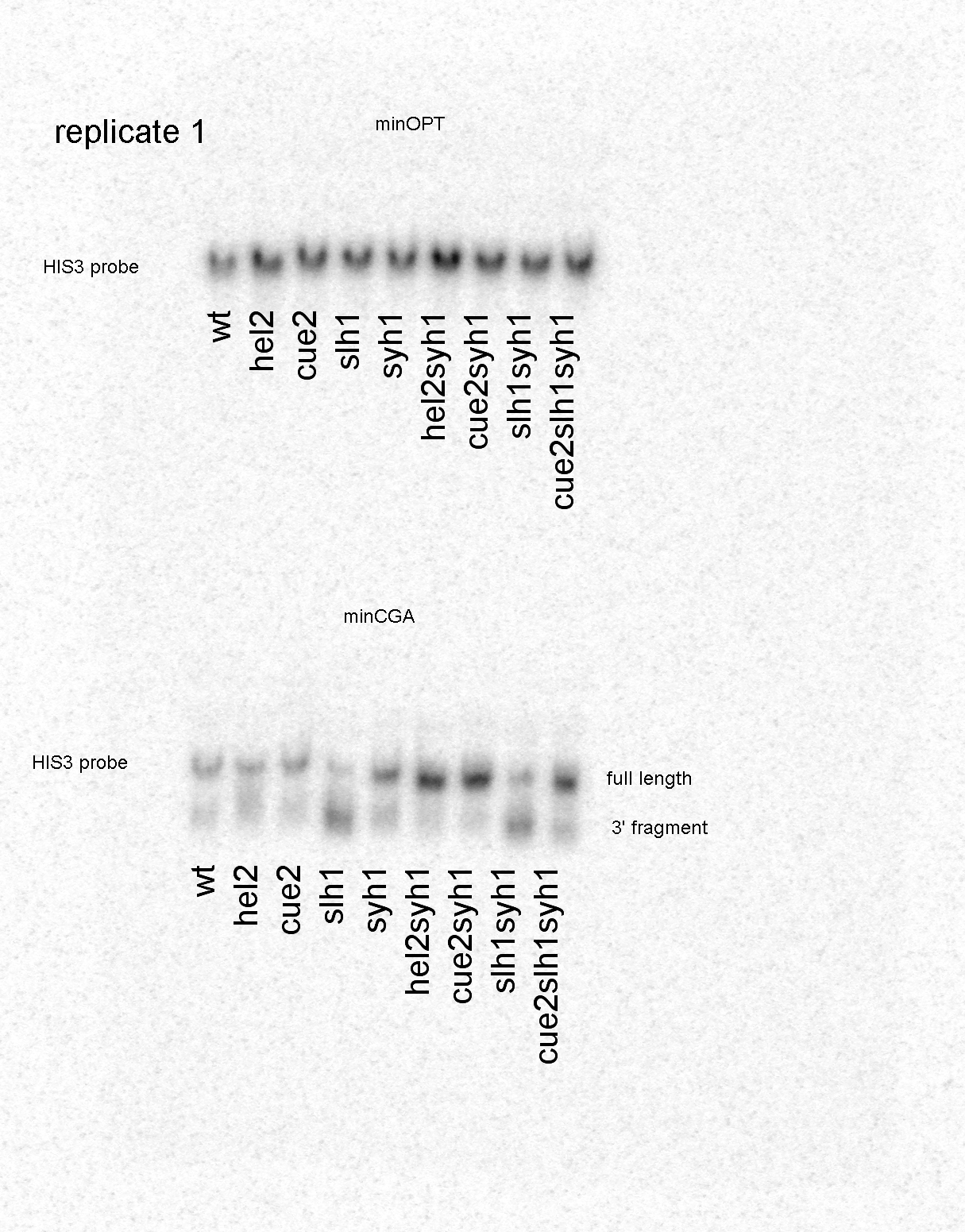

Supplement: Figure 2—source data 2. [file elife-76038-fig2-data2.zip › 2B-C_S2A_northen_blot_images/annotated_his3_rep_1.tif]

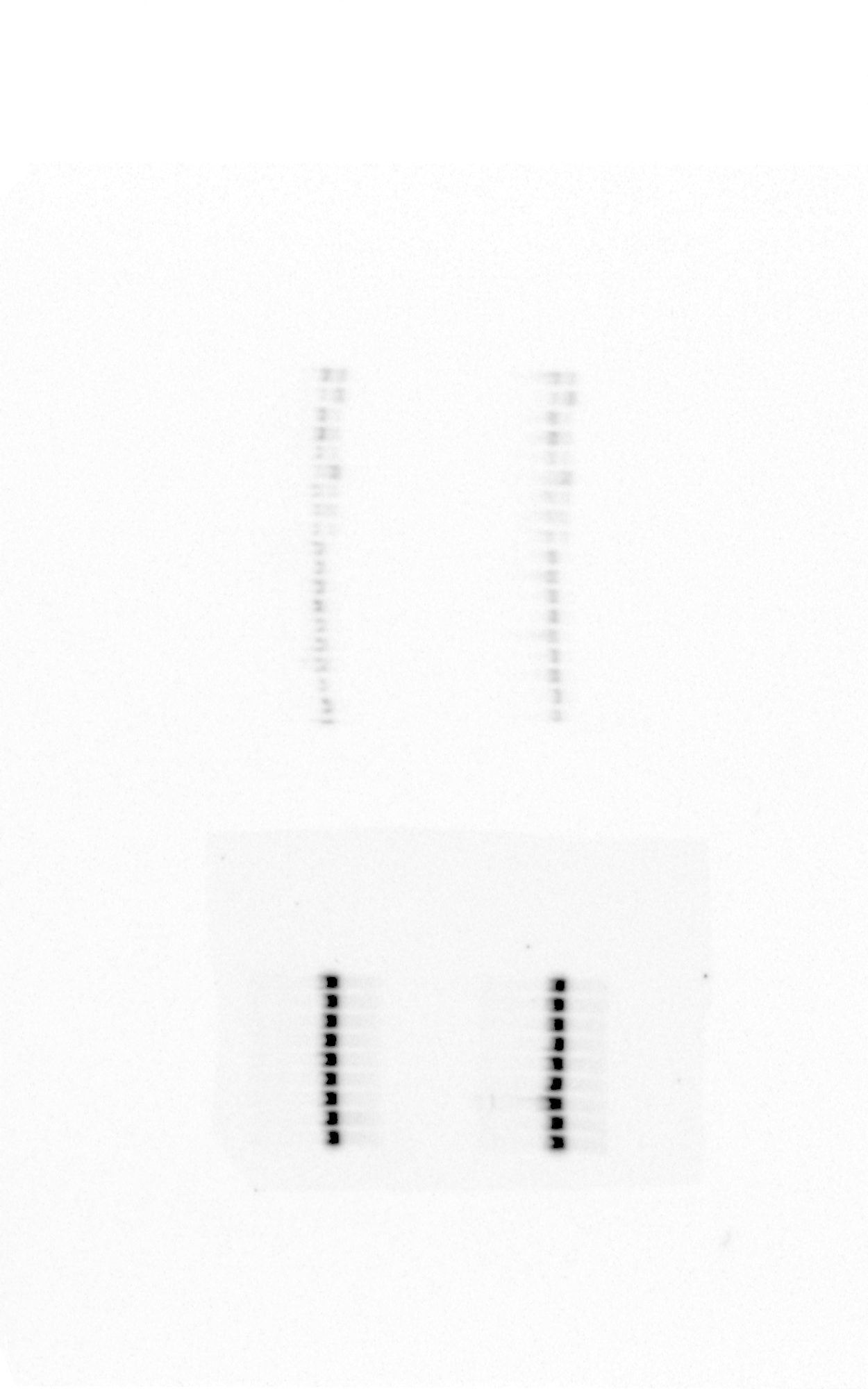

Supplement: Figure 2—source data 2. [file elife-76038-fig2-data2.zip › 2B-C_S2A_northen_blot_images/raw_image_his3_rep_2-3.gel]

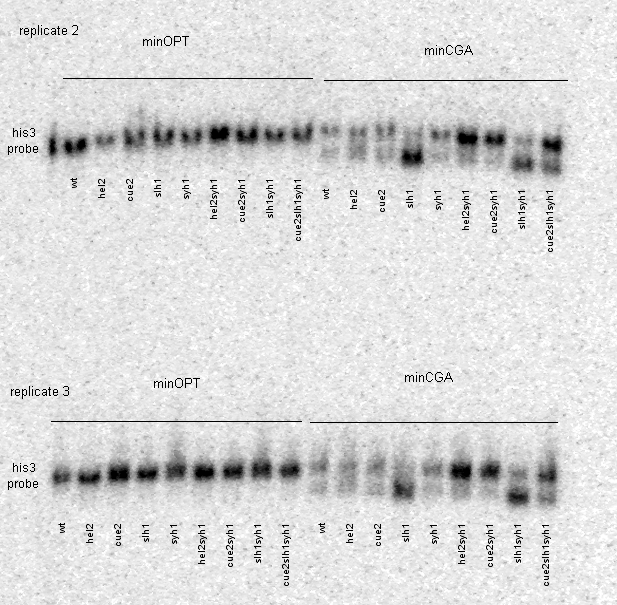

Supplement: Figure 2—source data 2. [file elife-76038-fig2-data2.zip › 2B-C_S2A_northen_blot_images/annotated_his3_reps_2-3.tif]

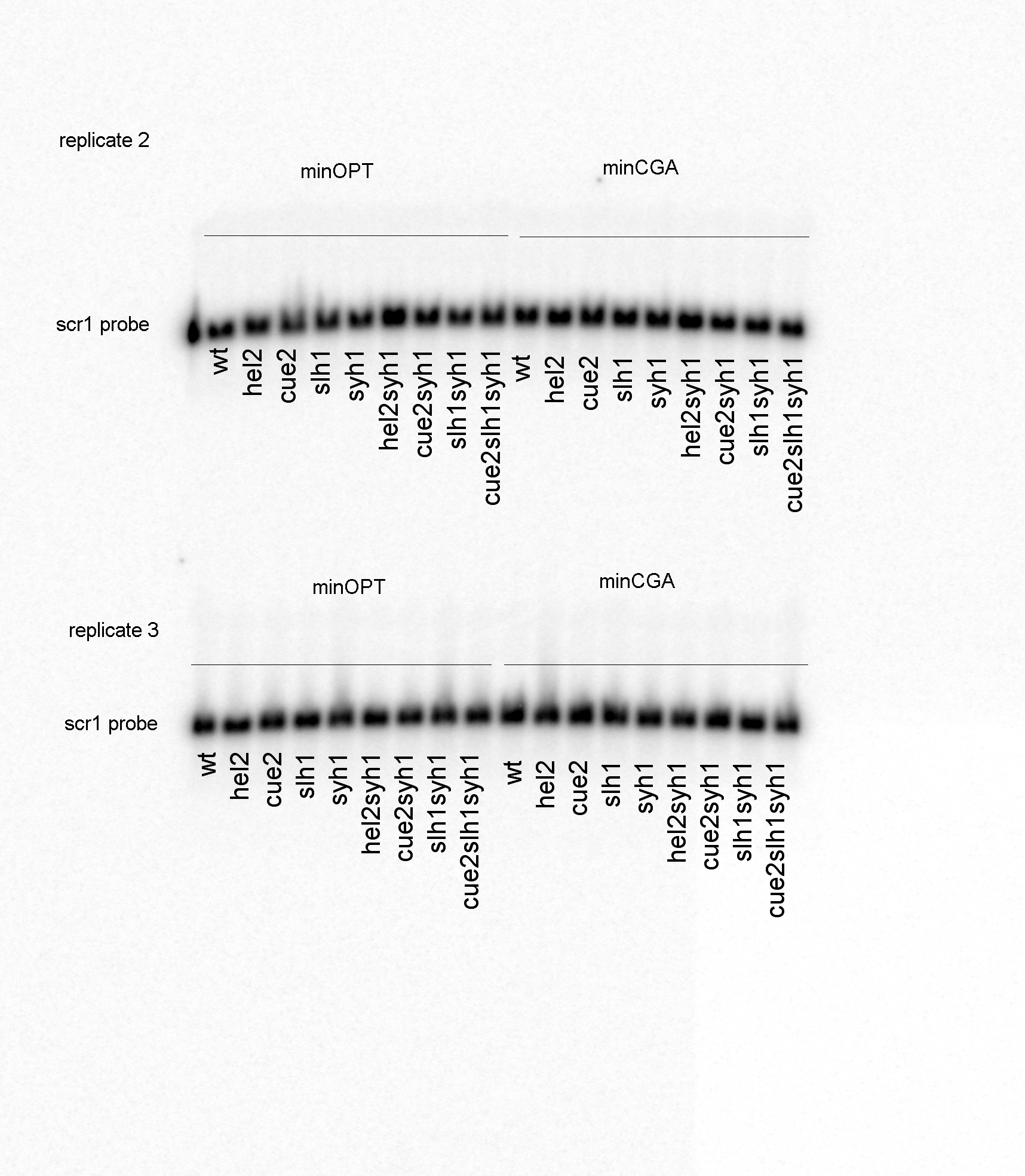

Supplement: Figure 2—source data 2. [file elife-76038-fig2-data2.zip › 2B-C_S2A_northen_blot_images/annotated_scr1_reps_2-3.tif]

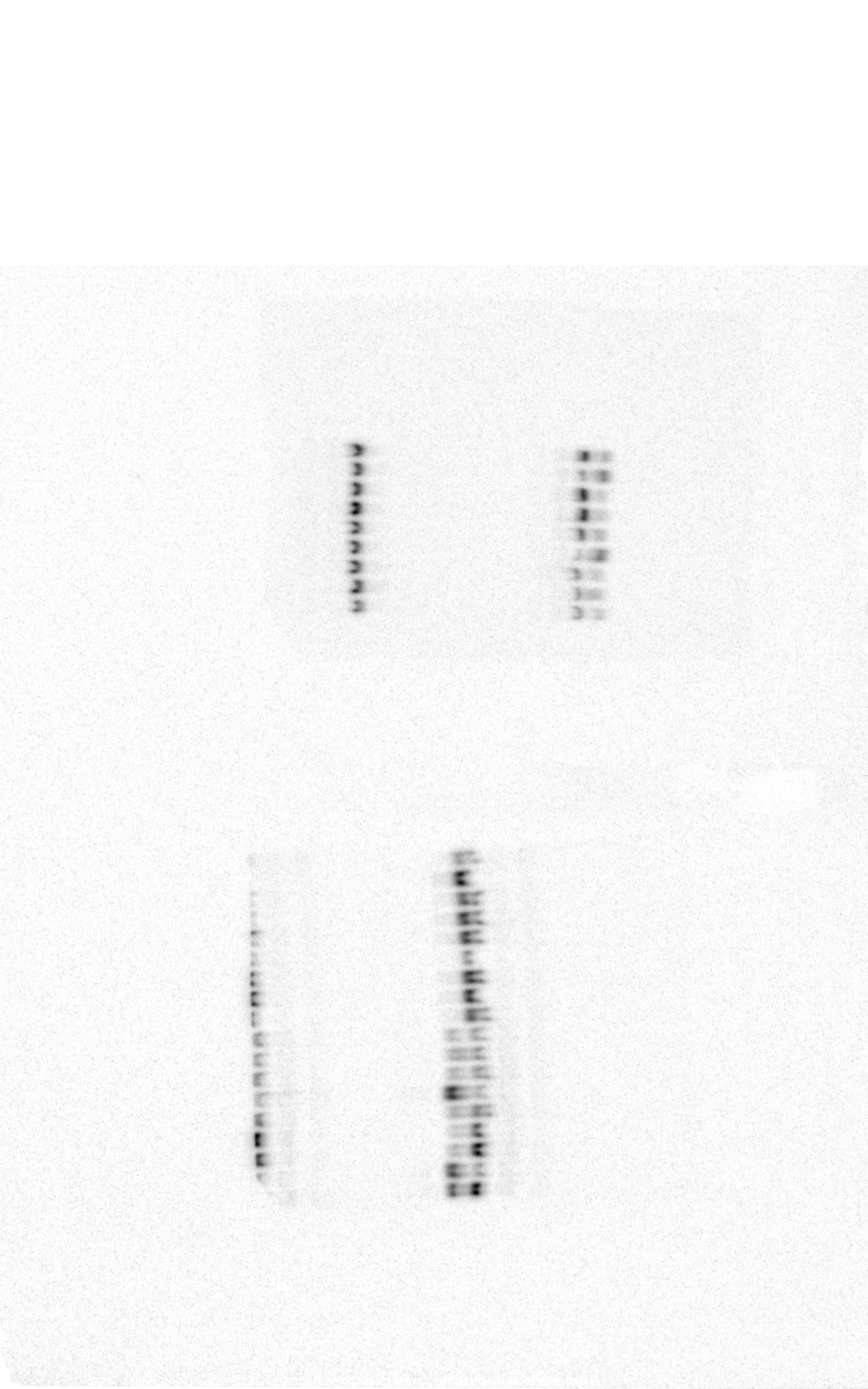

Supplement: Figure 2—source data 2. [file elife-76038-fig2-data2.zip › 2B-C_S2A_northen_blot_images/raw_image_his3_rep_1.gel]

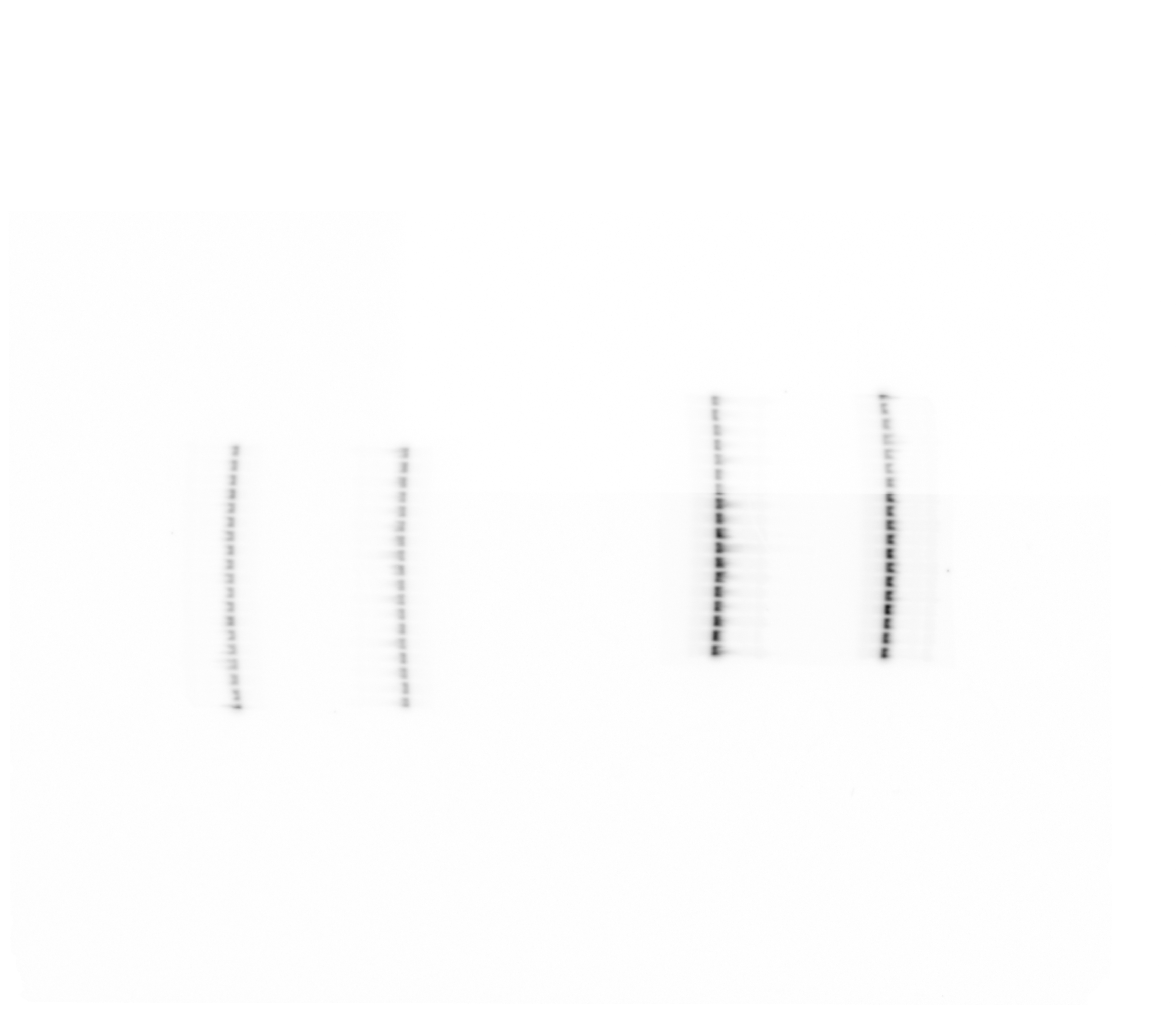

Supplement: Figure 2—source data 2. [file elife-76038-fig2-data2.zip › 2B-C_S2A_northen_blot_images/raw_image_scr1_rep_2-3.gel]

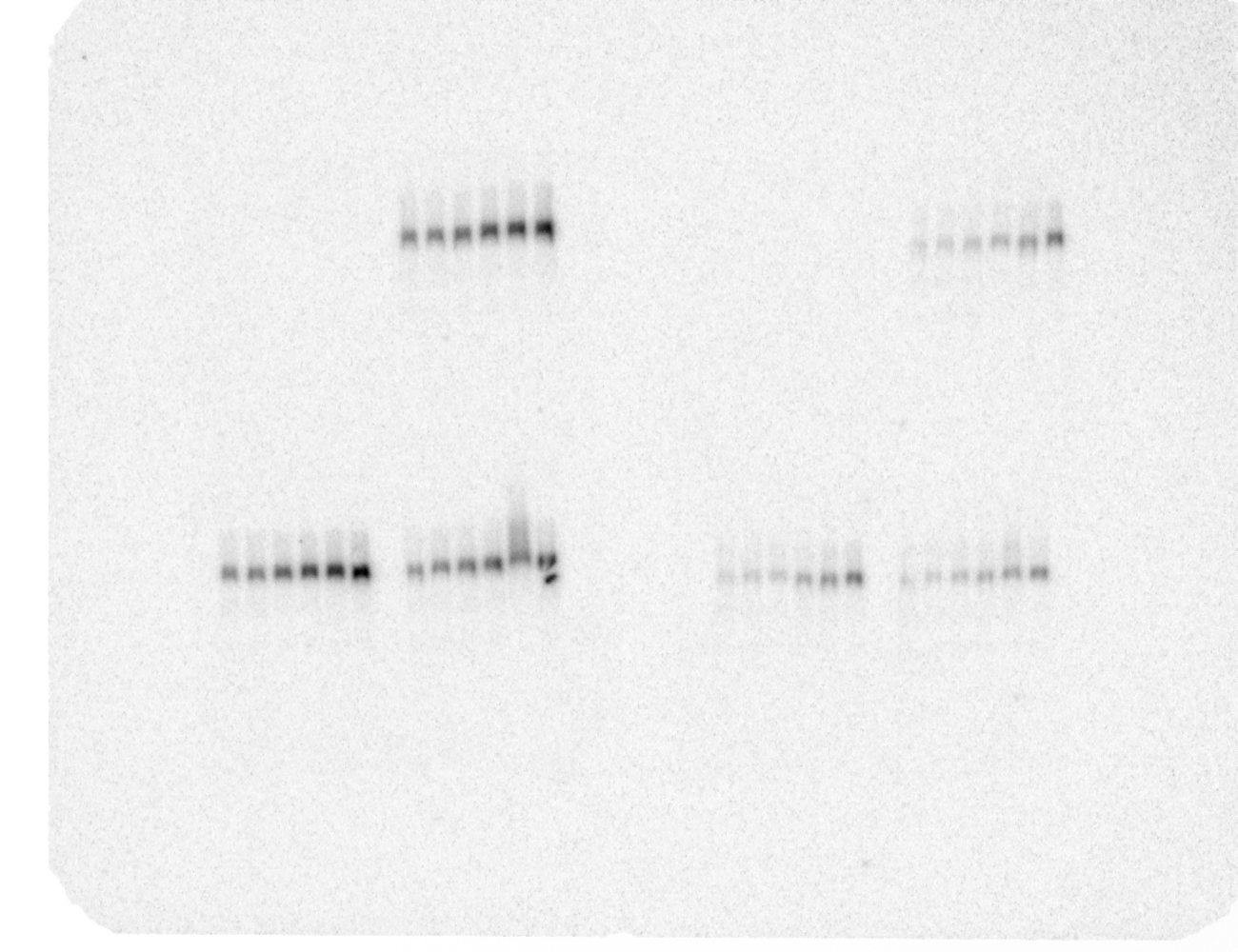

Supplement: Figure 4—source data 1. [file elife-76038-fig4-data1.zip › NOT5/minNONOPT/raw_image_his3_reps_1-3.gel]

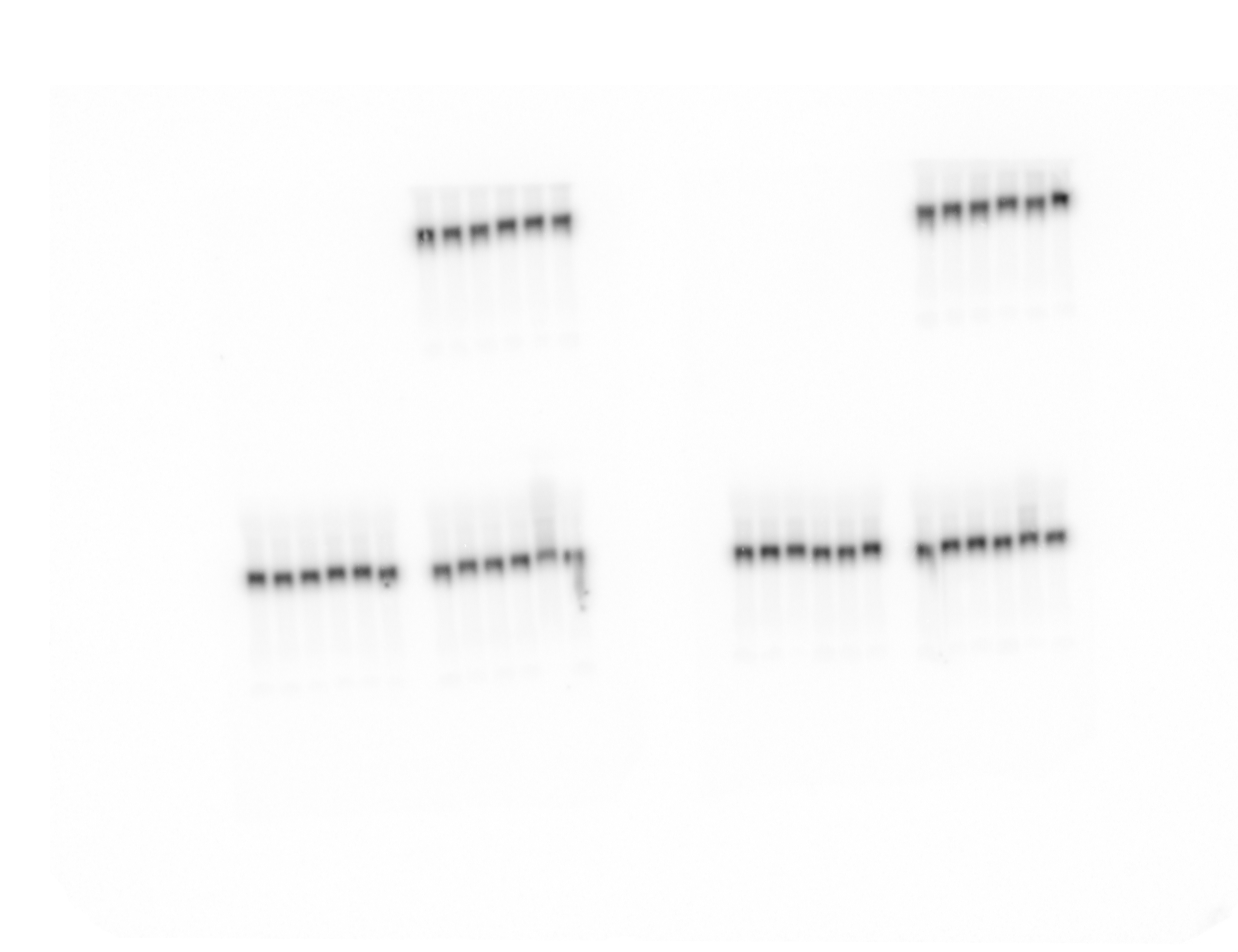

Supplement: Figure 4—source data 1. [file elife-76038-fig4-data1.zip › NOT5/minNONOPT/raw_image_scr1_reps_1-3.gel]

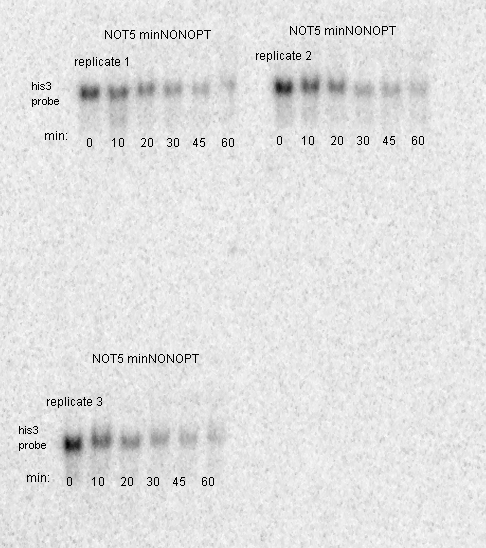

Supplement: Figure 4—source data 1. [file elife-76038-fig4-data1.zip › NOT5/minNONOPT/annotated_his3_reps_1-3.tif]

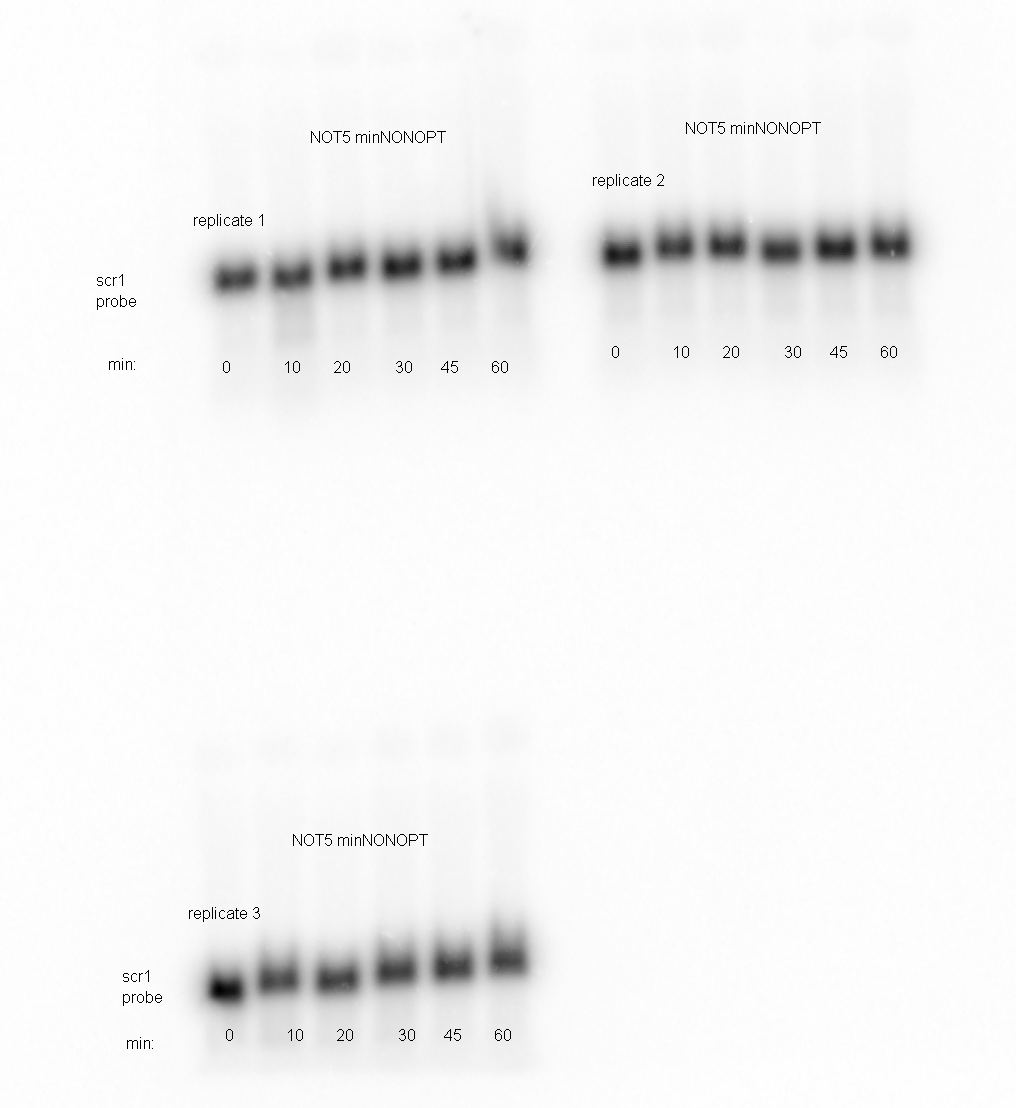

Supplement: Figure 4—source data 1. [file elife-76038-fig4-data1.zip › NOT5/minNONOPT/annotated_scr1_reps_1-3.tif]

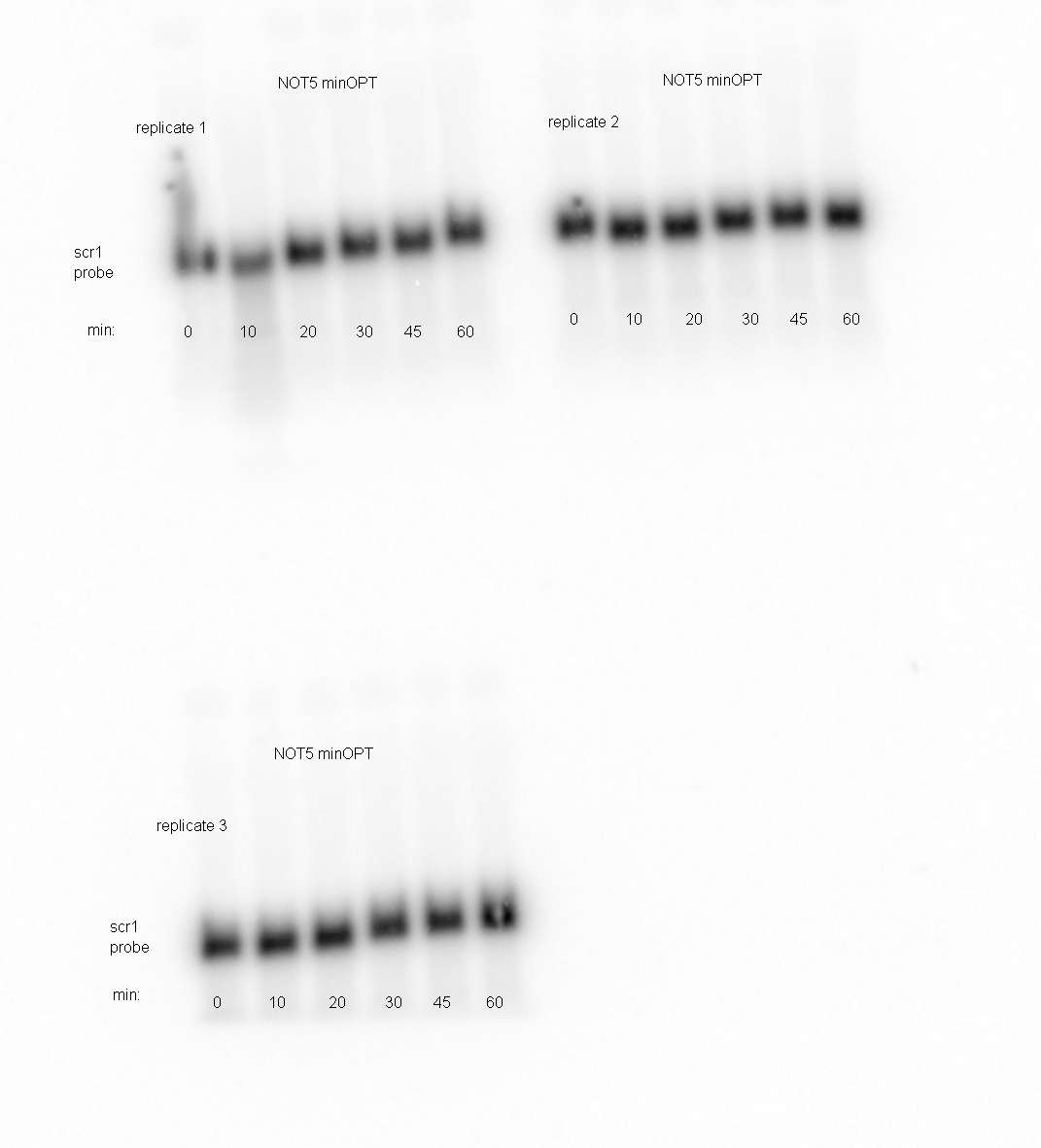

Supplement: Figure 4—source data 1. [file elife-76038-fig4-data1.zip › NOT5/minOPT/annotated_scr1_reps_1-3.tif]

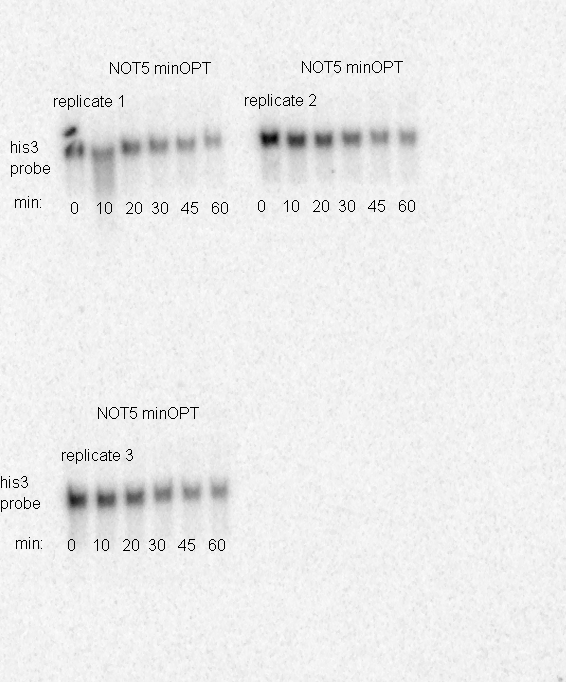

Supplement: Figure 4—source data 1. [file elife-76038-fig4-data1.zip › NOT5/minOPT/annotated_his3_reps_1-3.tif]

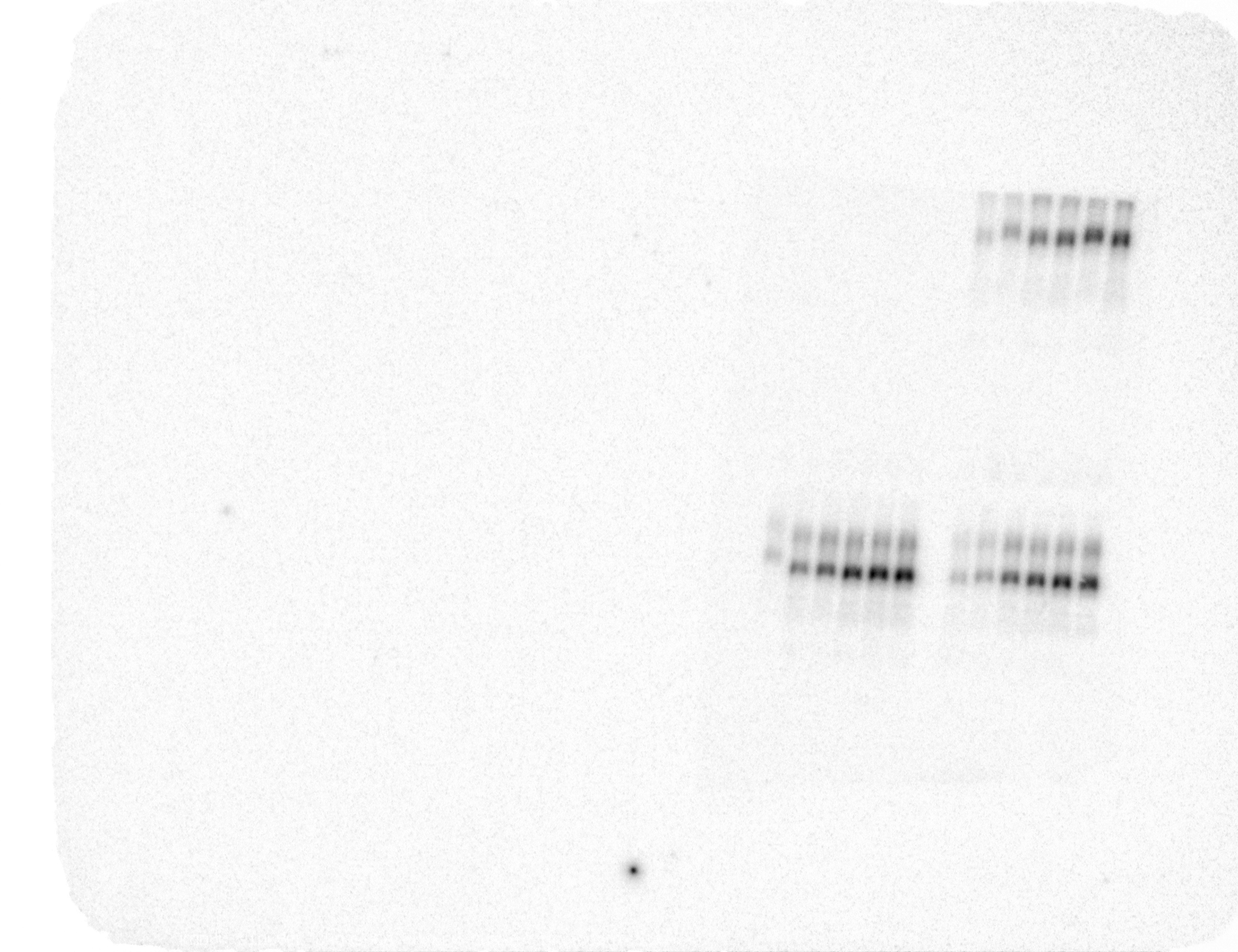

Supplement: Figure 4—source data 1. [file elife-76038-fig4-data1.zip › NOT5/minCGA/raw_image_his3_reps_1-2.gel]

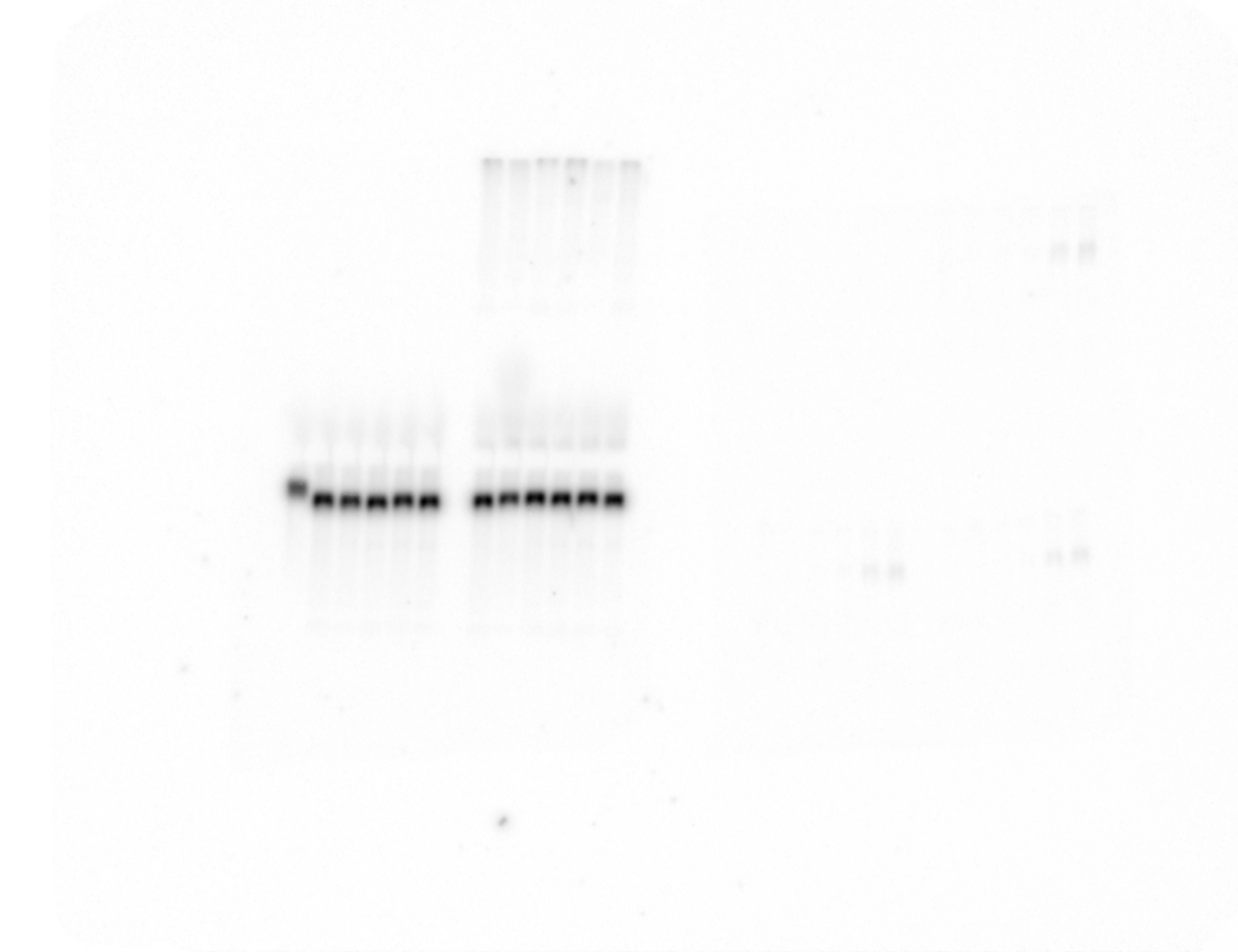

Supplement: Figure 4—source data 1. [file elife-76038-fig4-data1.zip › NOT5/minCGA/raw_image_scr1_reps_1-2.gel]

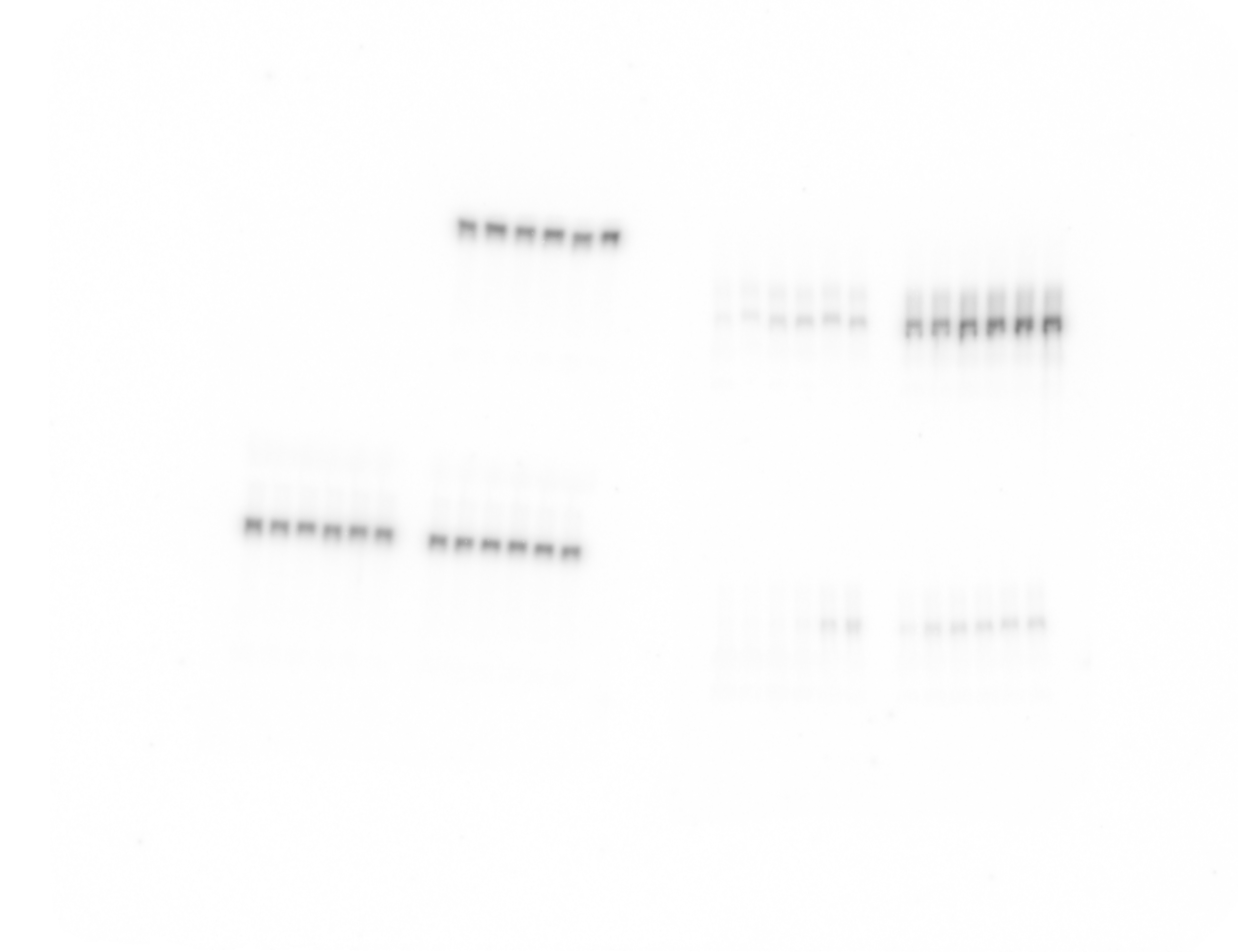

Supplement: Figure 4—source data 1. [file elife-76038-fig4-data1.zip › NOT5/minCGA/raw_image_his3_rep_3.gel]

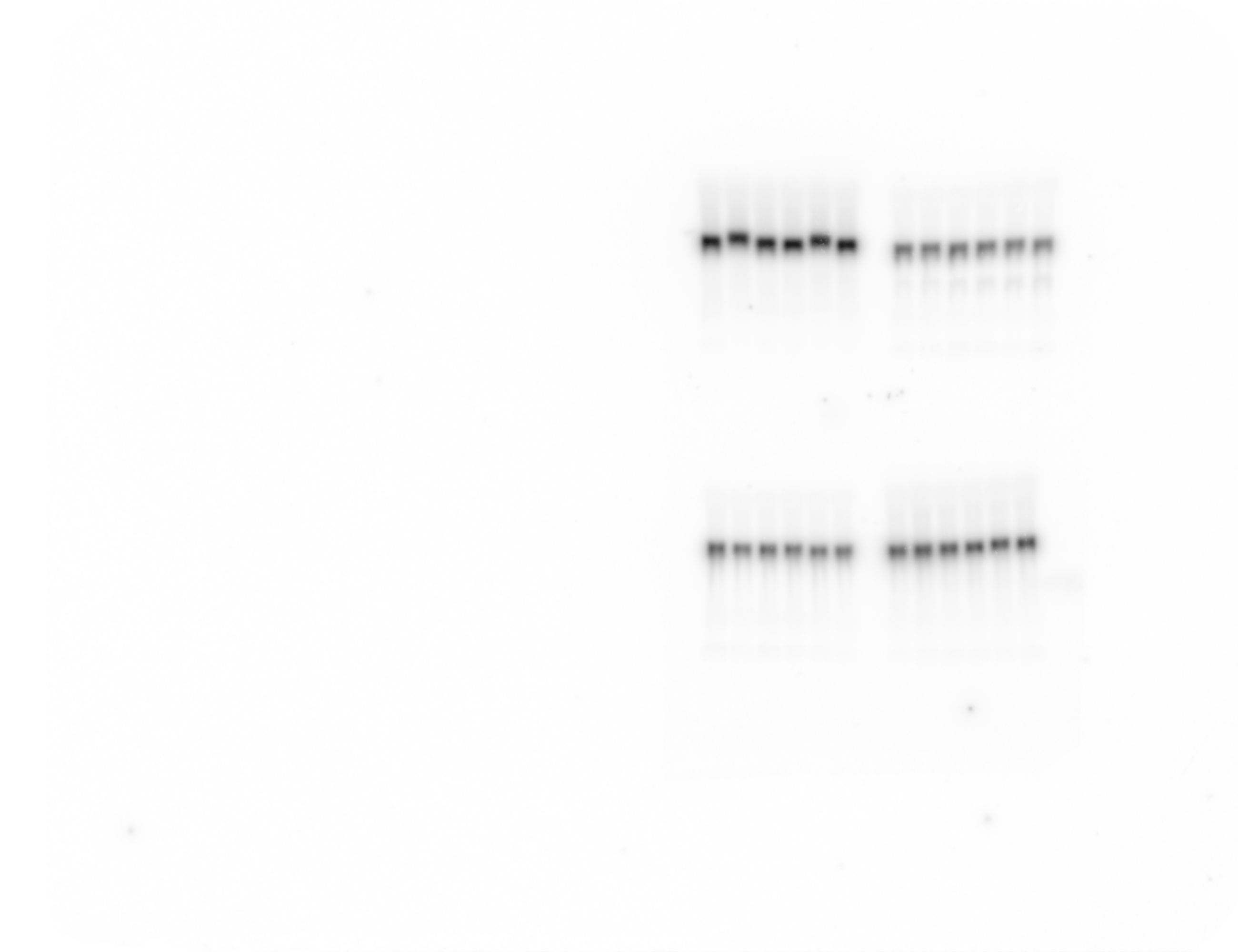

Supplement: Figure 4—source data 1. [file elife-76038-fig4-data1.zip › NOT5/minCGA/raw_image_scr1_rep_3.gel]

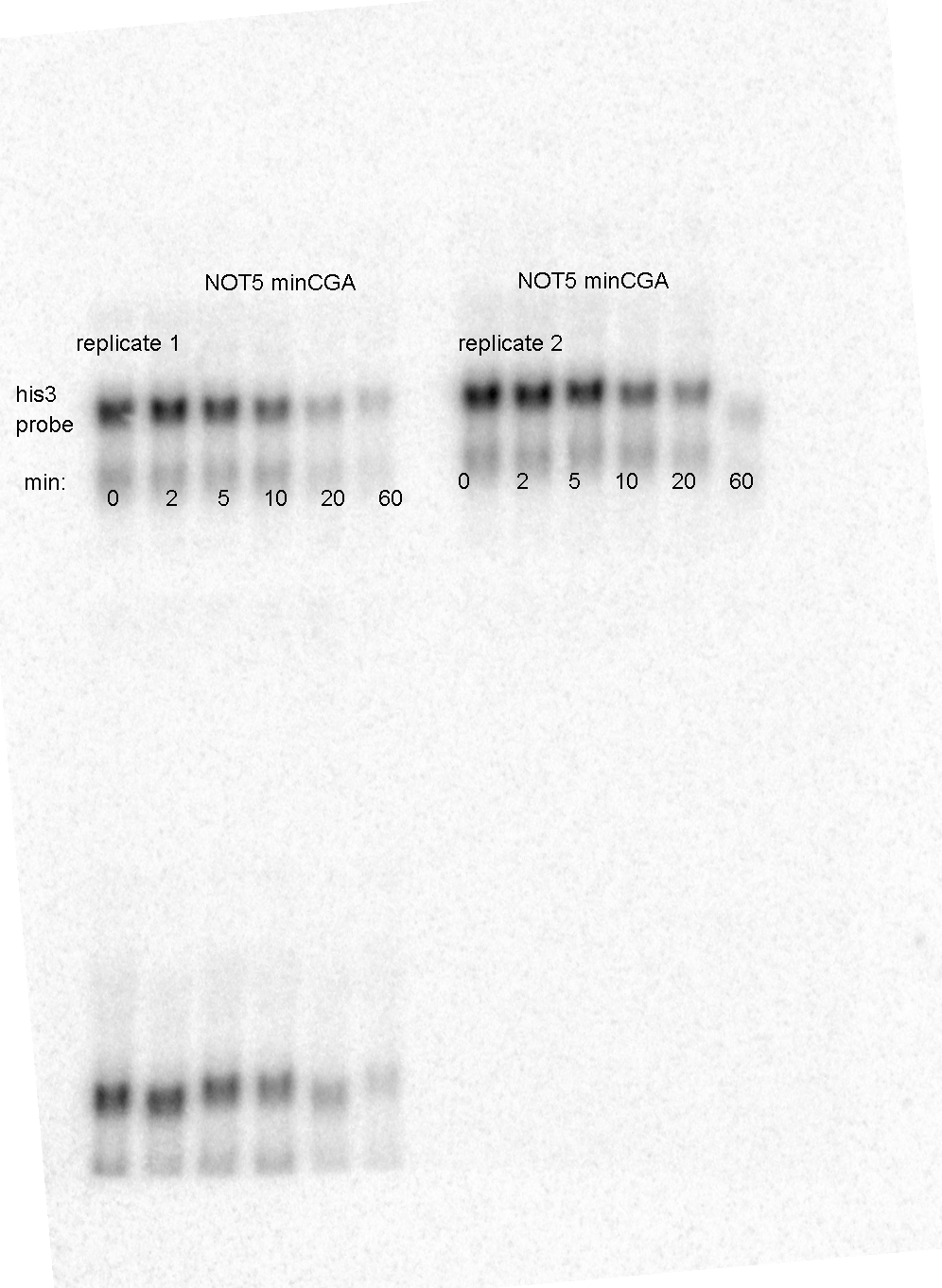

Supplement: Figure 4—source data 1. [file elife-76038-fig4-data1.zip › NOT5/minCGA/annotated_his3_reps_1-2.tif]

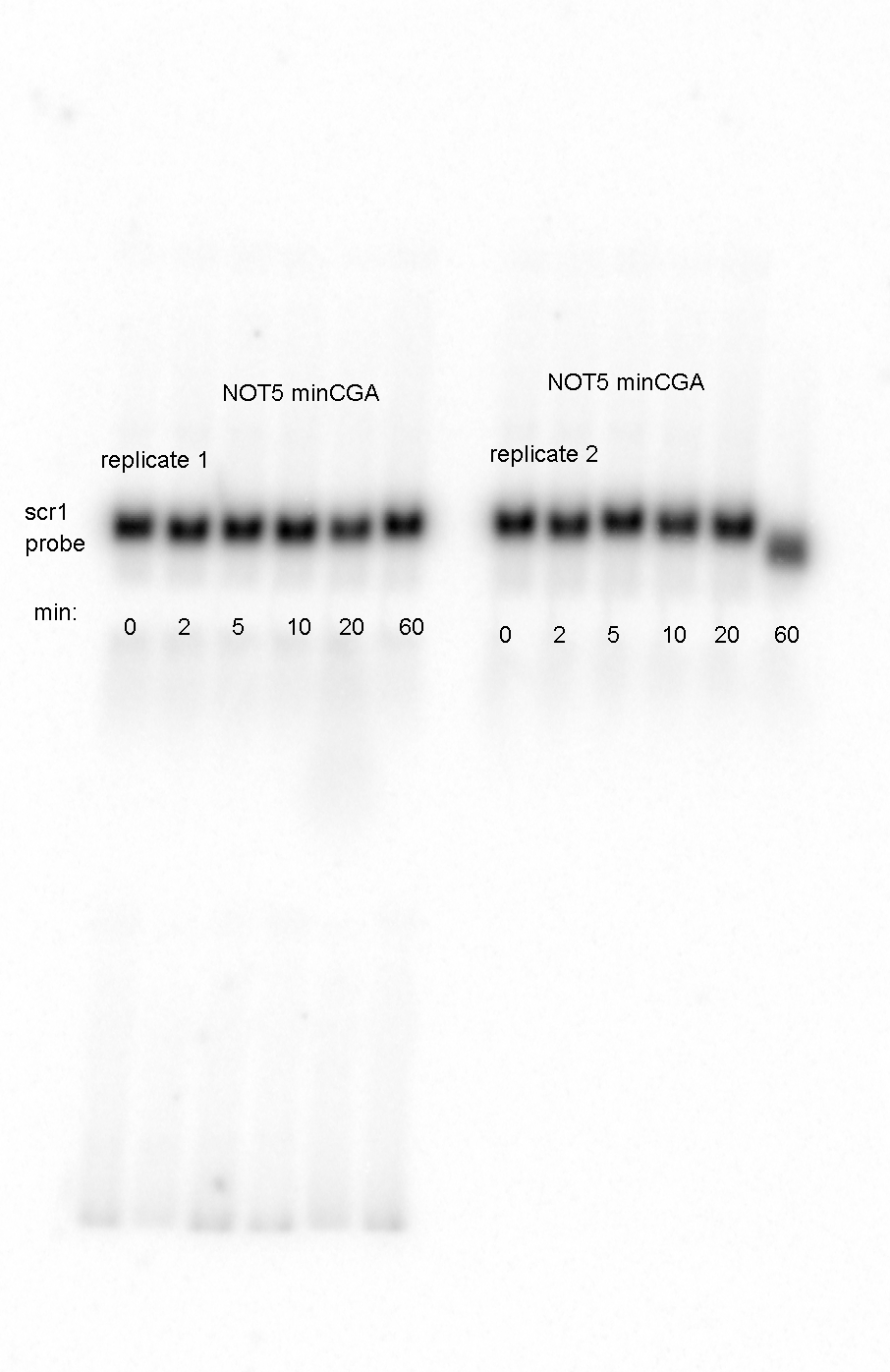

Supplement: Figure 4—source data 1. [file elife-76038-fig4-data1.zip › NOT5/minCGA/annotated_scr1_reps_1-2.tif]

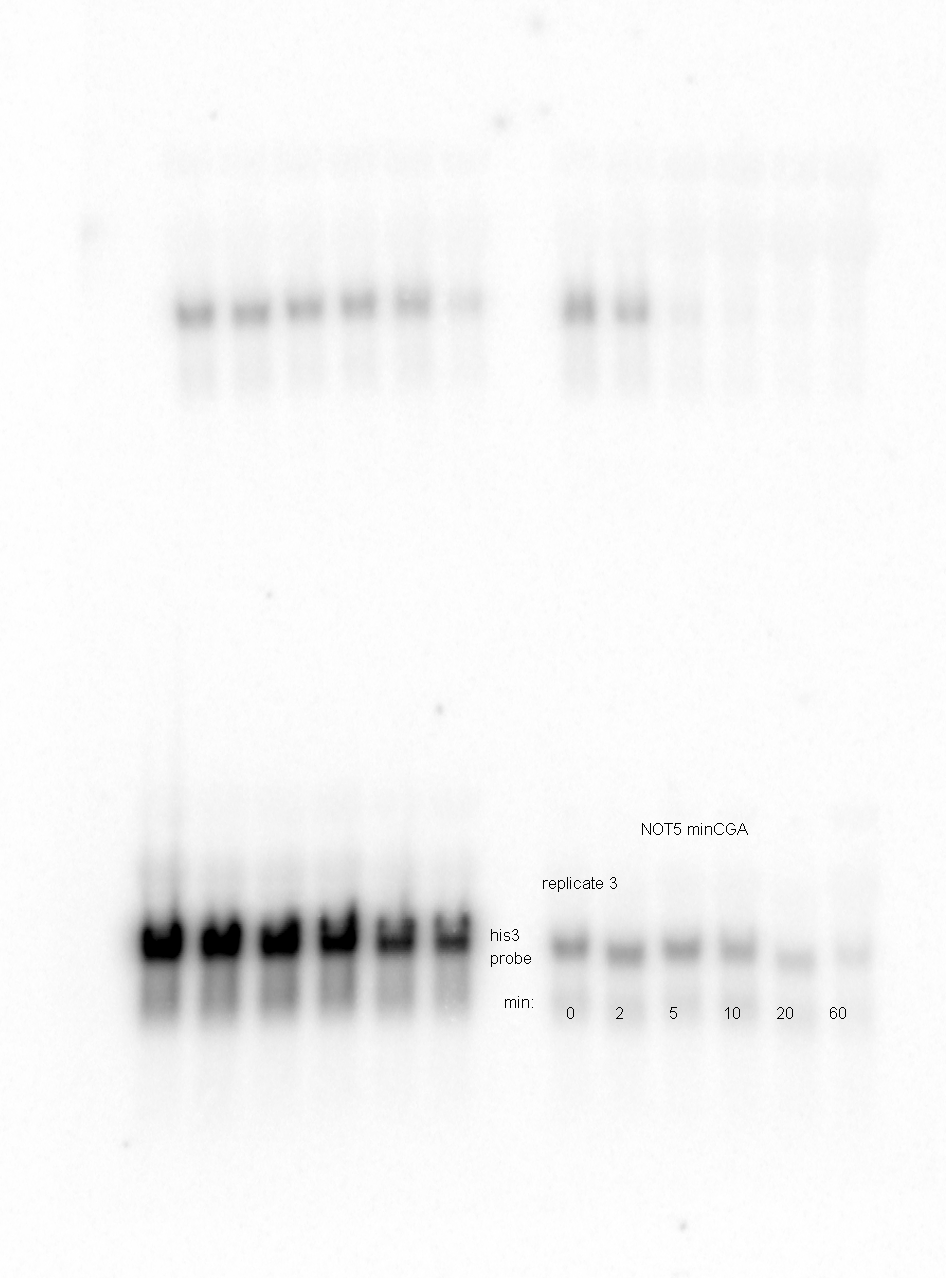

Supplement: Figure 4—source data 1. [file elife-76038-fig4-data1.zip › NOT5/minCGA/annotated_his3_rep_3.tif]

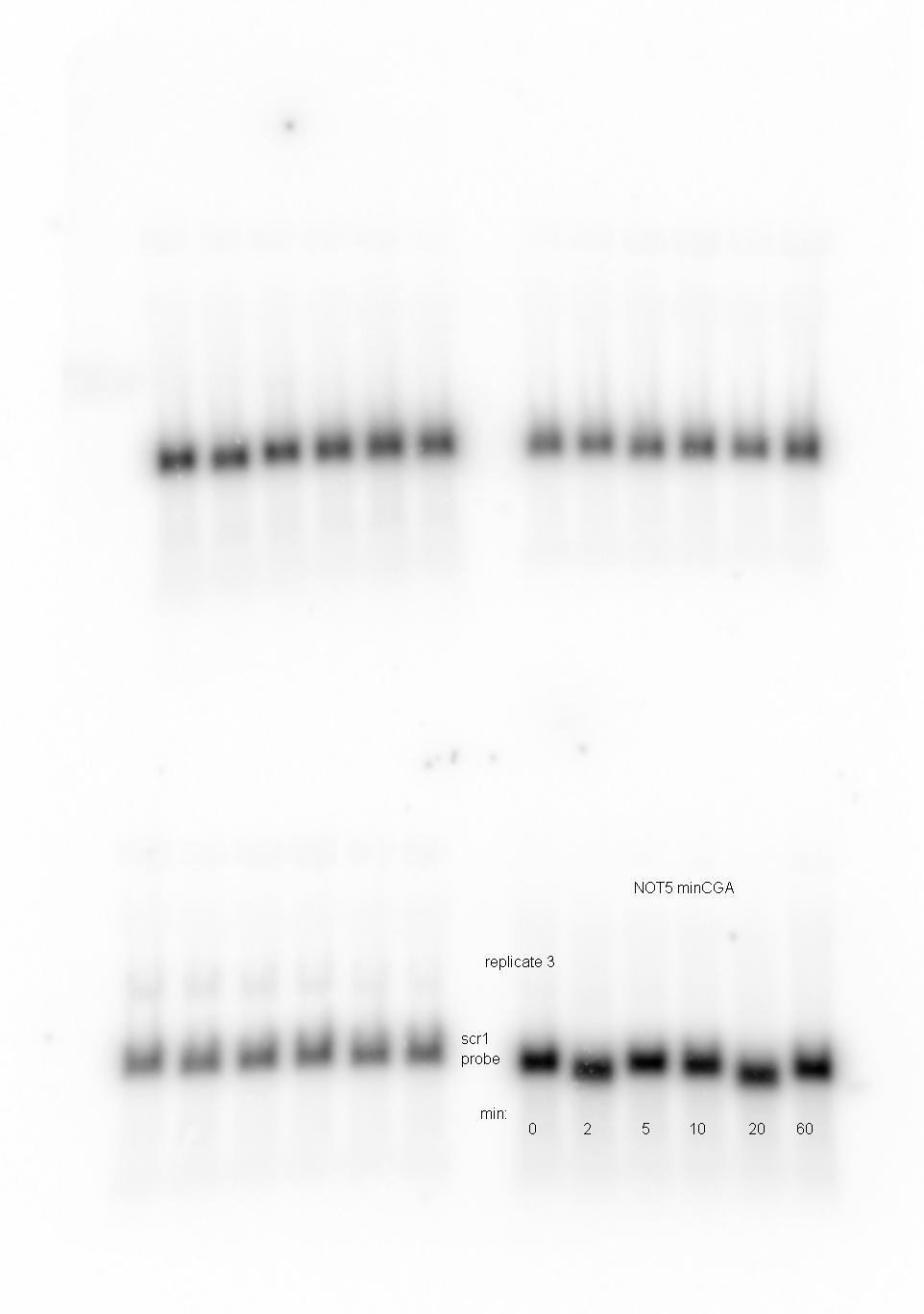

Supplement: Figure 4—source data 1. [file elife-76038-fig4-data1.zip › NOT5/minCGA/annotated_scr1_rep_3.tif]

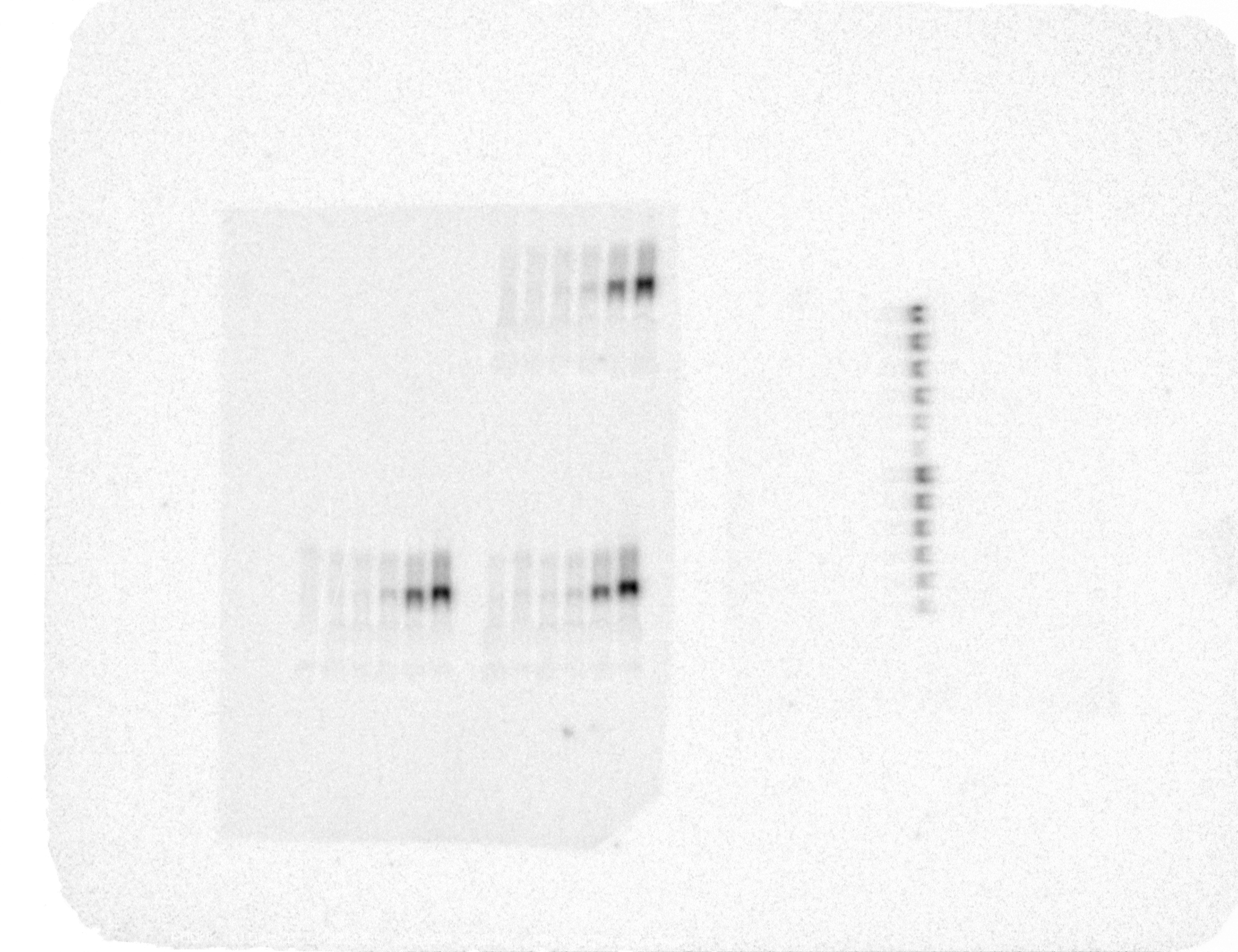

Supplement: Figure 4—source data 2. [file elife-76038-fig4-data2.zip › SYH1/minNONOPT/raw_image_his3_reps_1-3.gel]

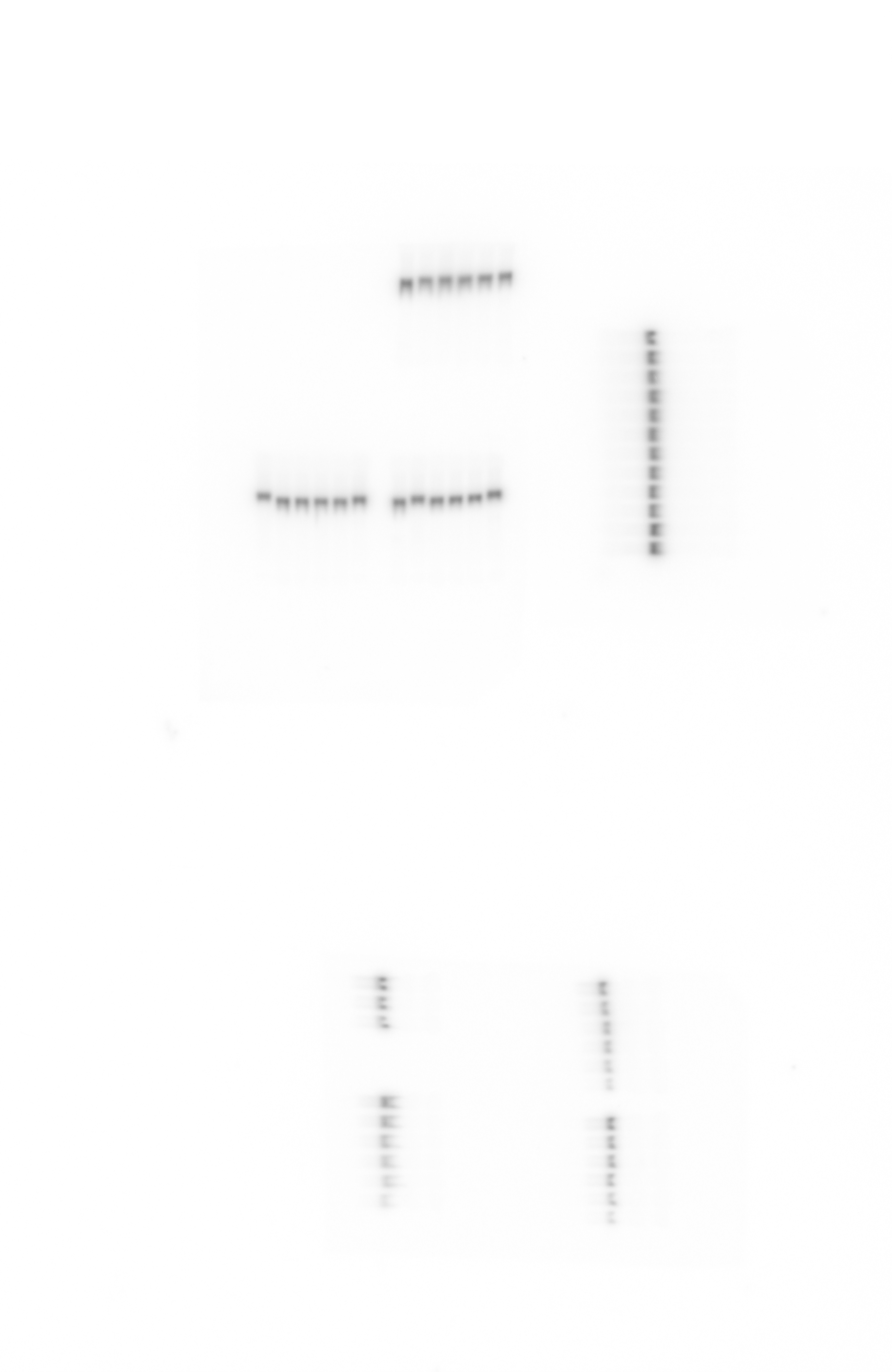

Supplement: Figure 4—source data 2. [file elife-76038-fig4-data2.zip › SYH1/minNONOPT/raw_image_scr1_reps_1-3.gel]

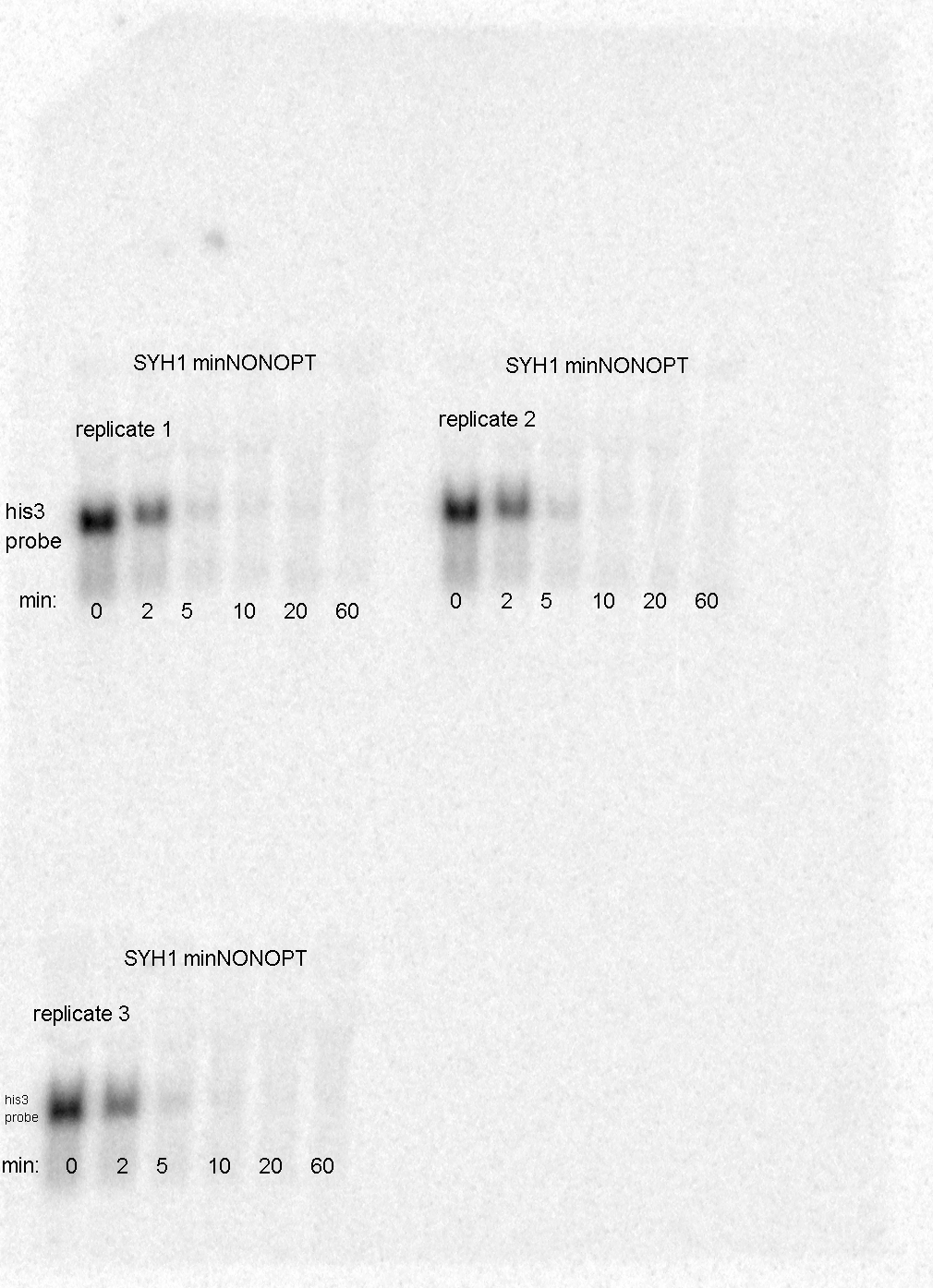

Supplement: Figure 4—source data 2. [file elife-76038-fig4-data2.zip › SYH1/minNONOPT/annotated_his3_reps_1-3.tif]

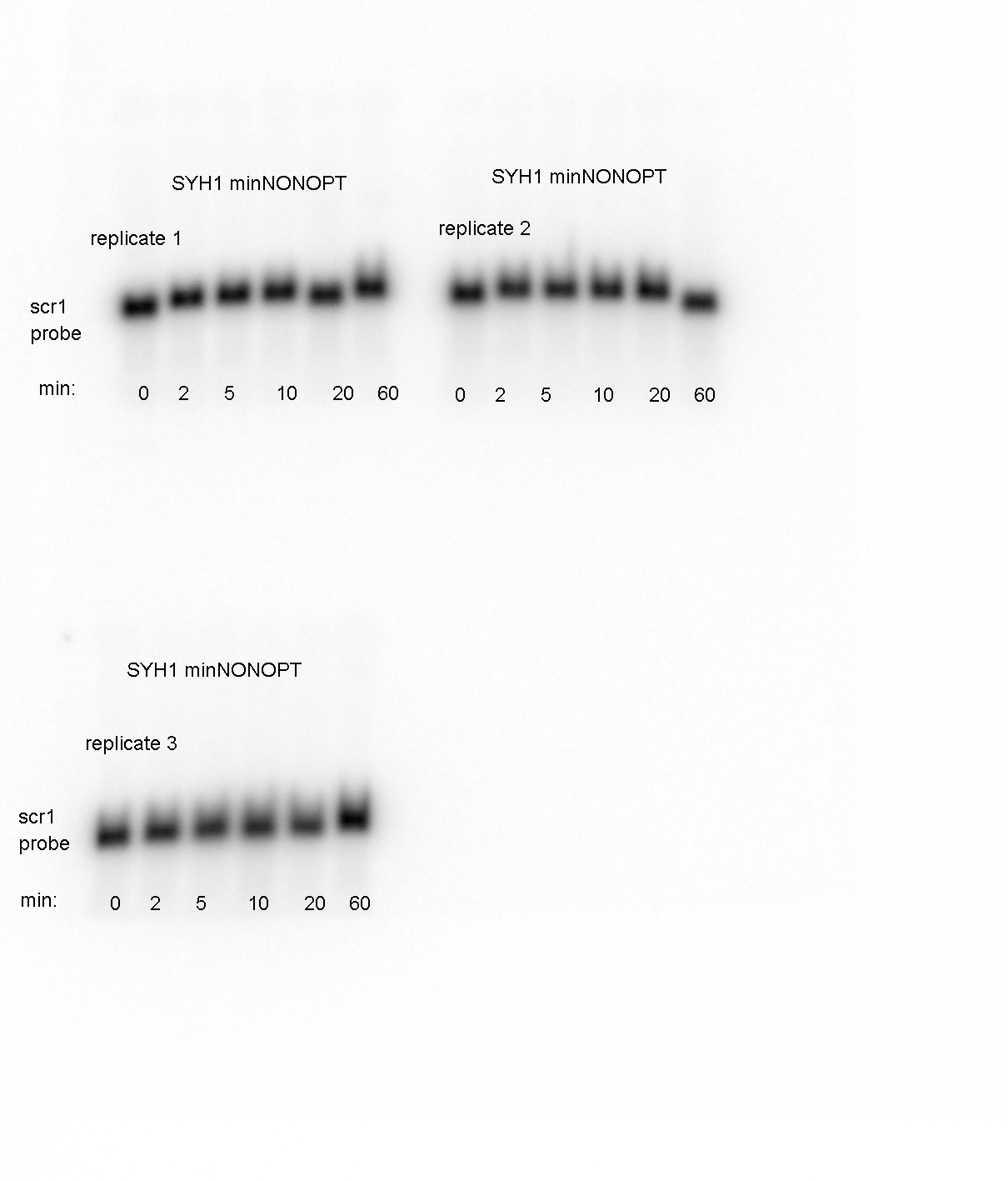

Supplement: Figure 4—source data 2. [file elife-76038-fig4-data2.zip › SYH1/minNONOPT/annotated_scr1_reps_1-3.tif]

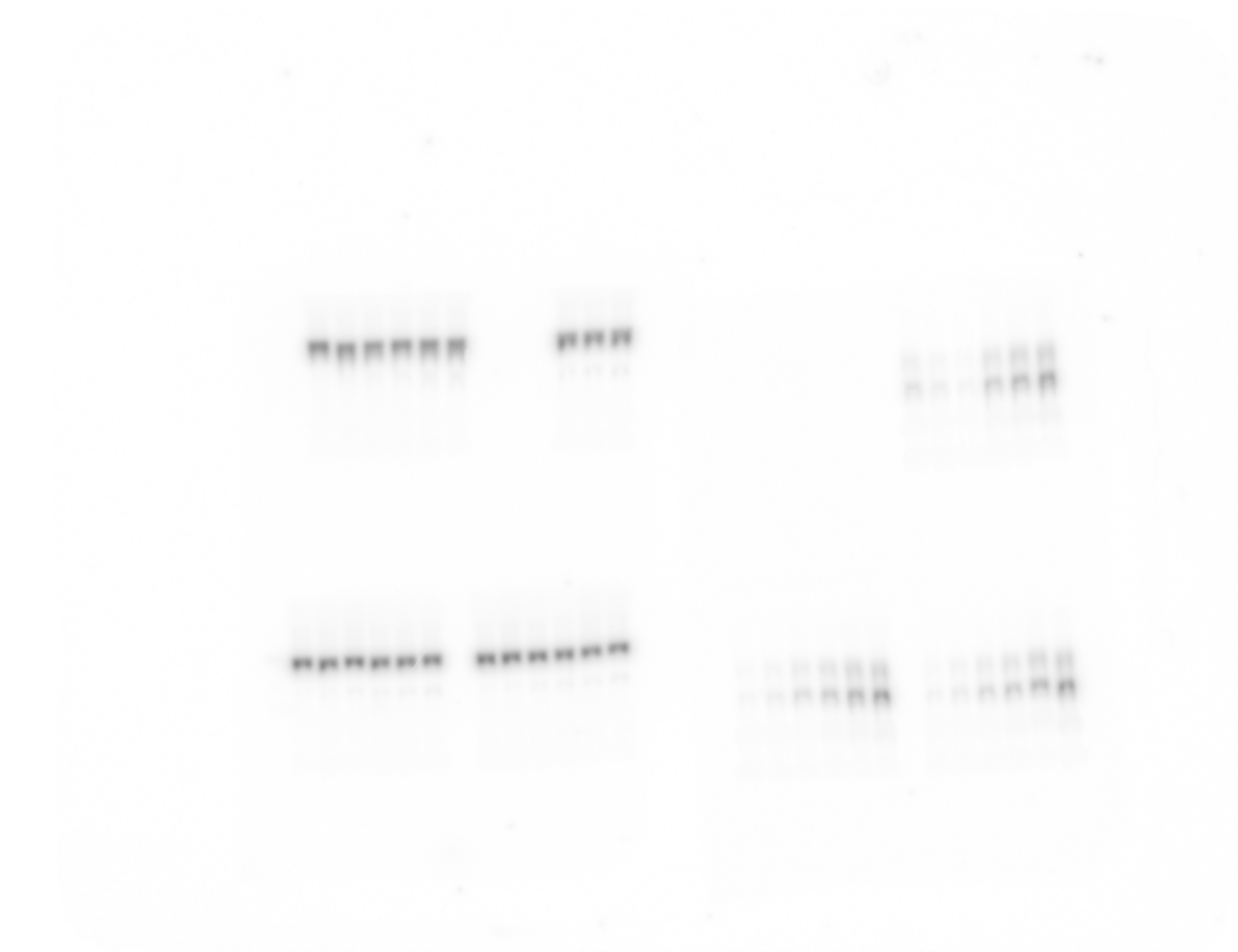

Supplement: Figure 4—source data 2. [file elife-76038-fig4-data2.zip › SYH1/minOPT/raw_image_scr1_reps_1-3.gel]

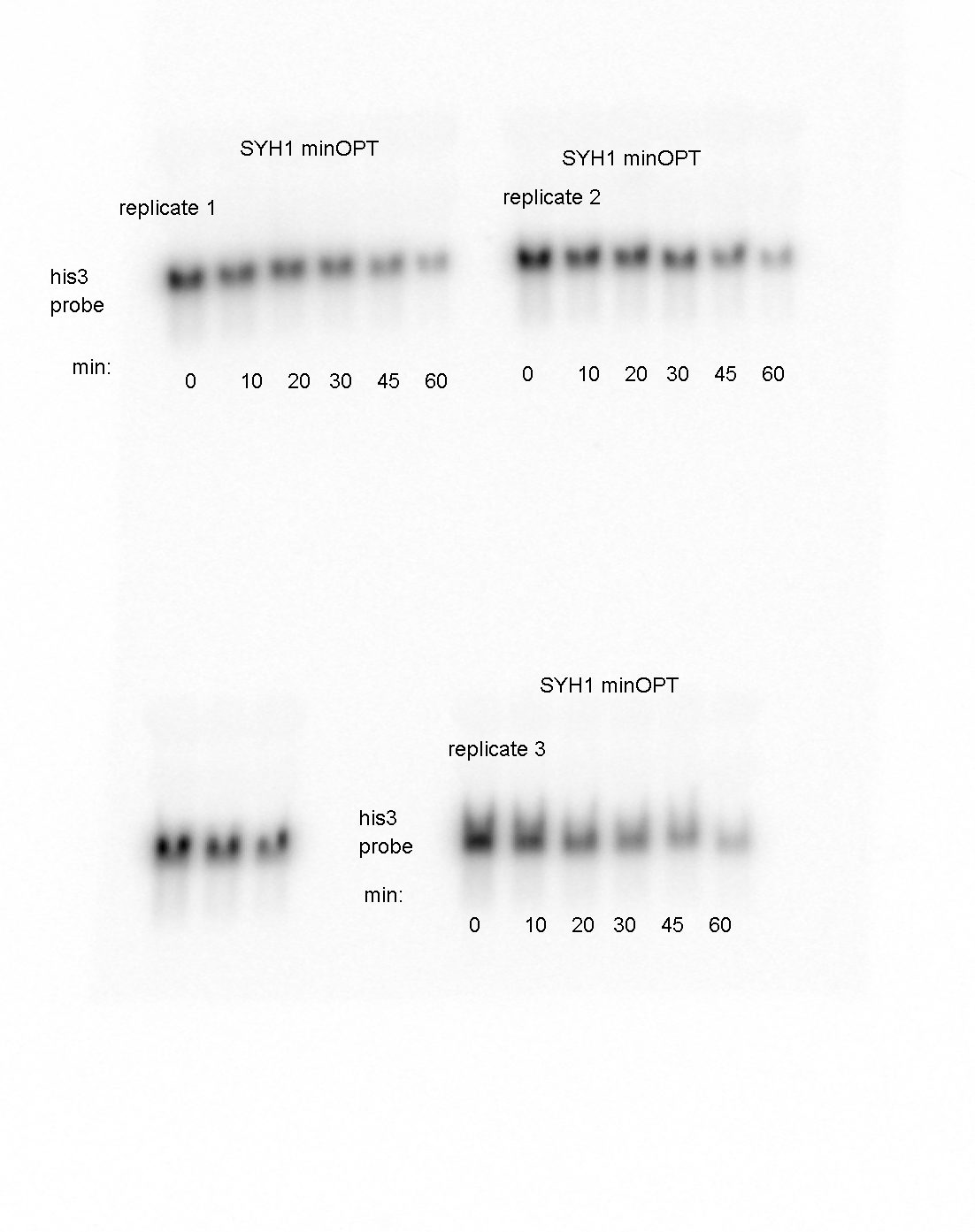

Supplement: Figure 4—source data 2. [file elife-76038-fig4-data2.zip › SYH1/minOPT/annotated_his3_reps_1-3.tif]

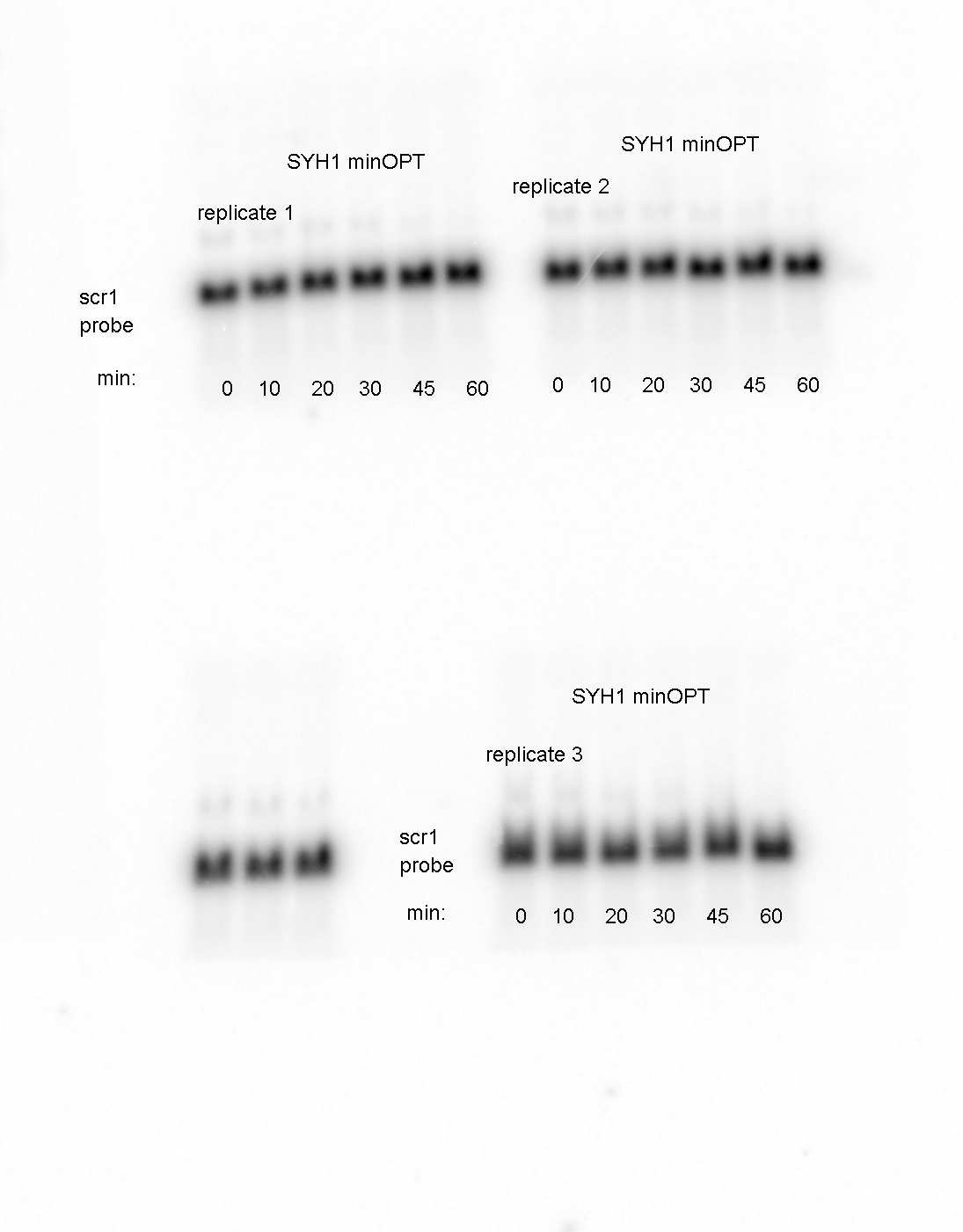

Supplement: Figure 4—source data 2. [file elife-76038-fig4-data2.zip › SYH1/minOPT/annotated_scr1_reps_1-3.tif]

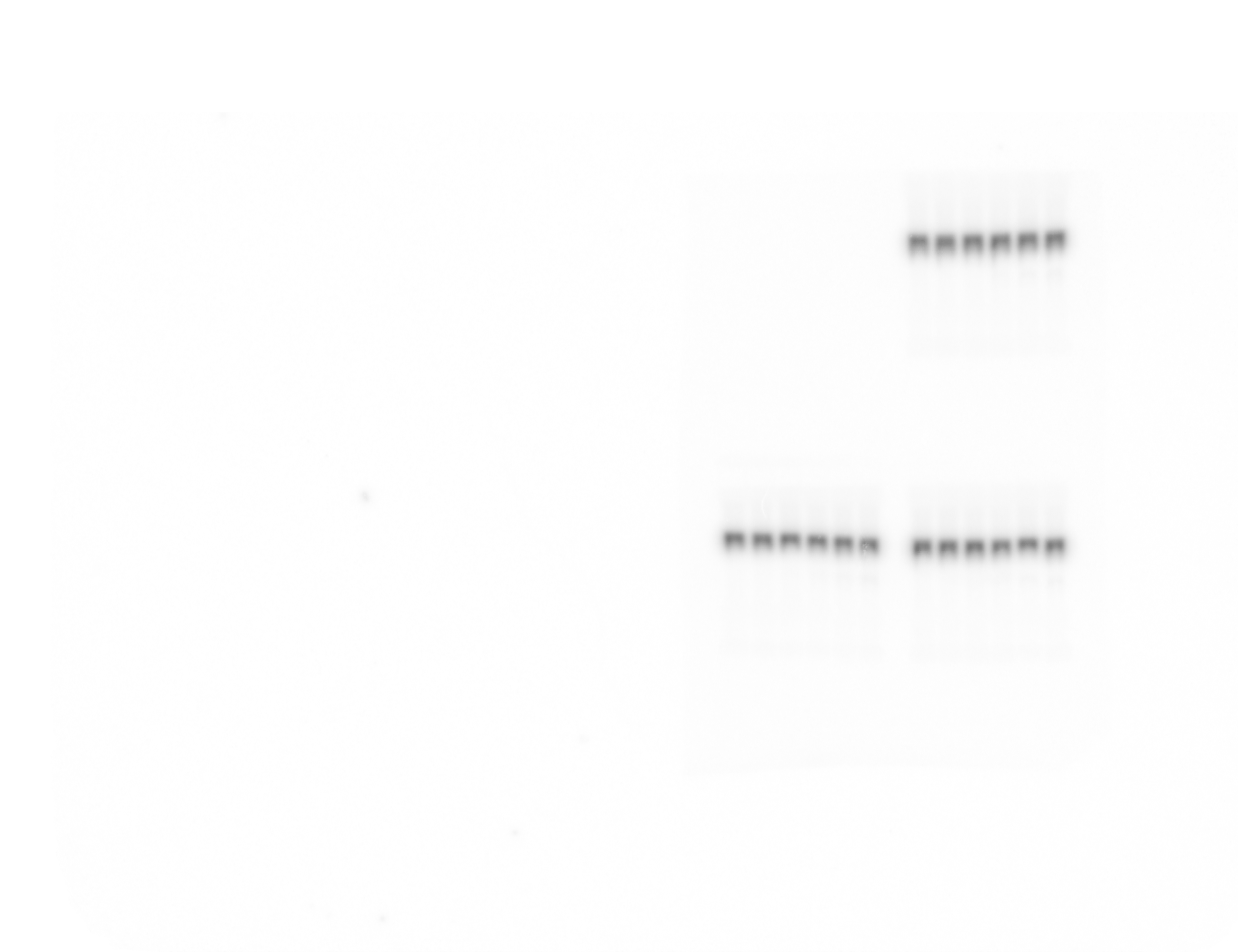

Supplement: Figure 4—source data 2. [file elife-76038-fig4-data2.zip › SYH1/minCGA/raw_image_scr1_reps_1-2.gel]

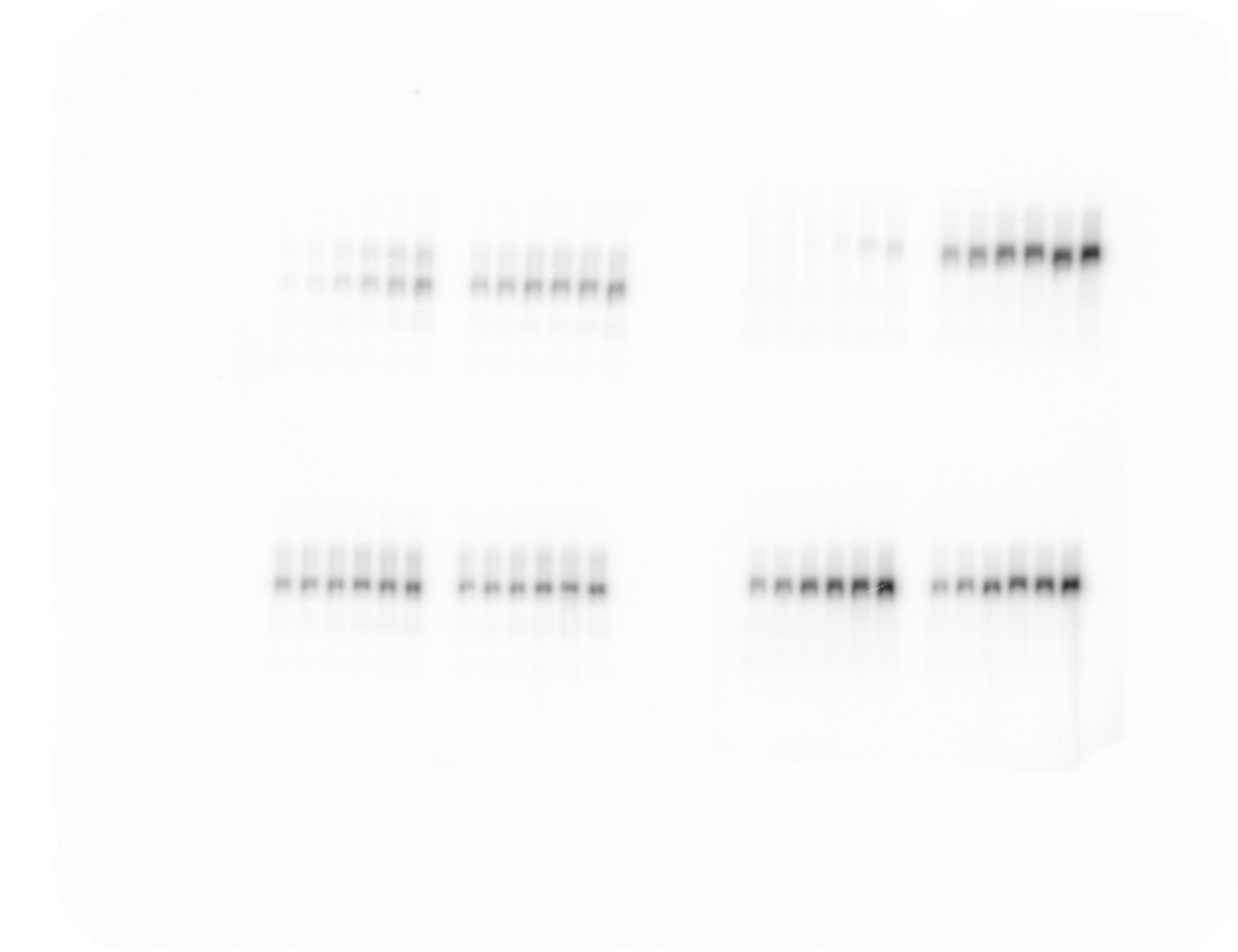

Supplement: Figure 4—source data 2. [file elife-76038-fig4-data2.zip › SYH1/minCGA/raw_image_his3_rep_3.gel]

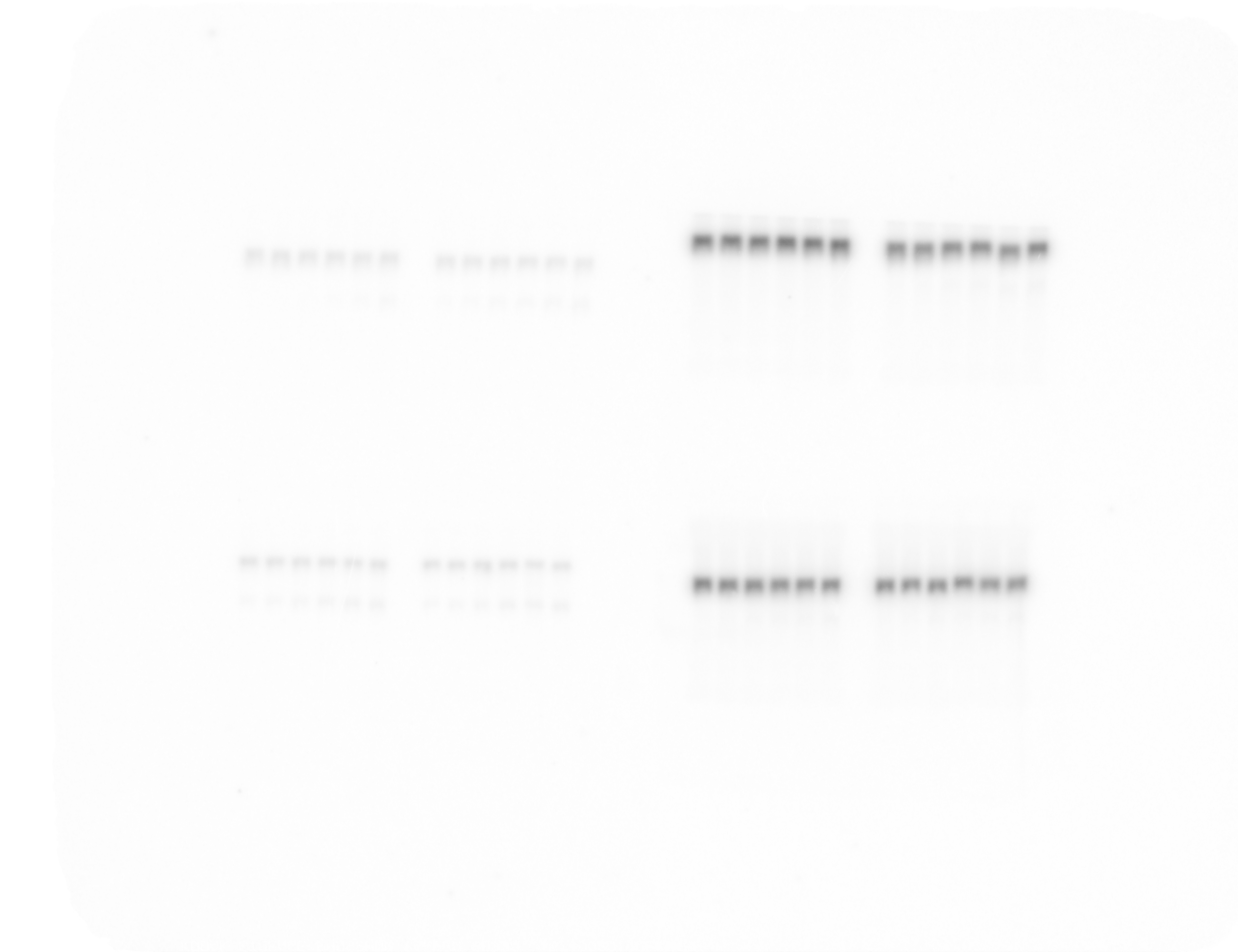

Supplement: Figure 4—source data 2. [file elife-76038-fig4-data2.zip › SYH1/minCGA/raw_image_scr1_rep_3.gel]

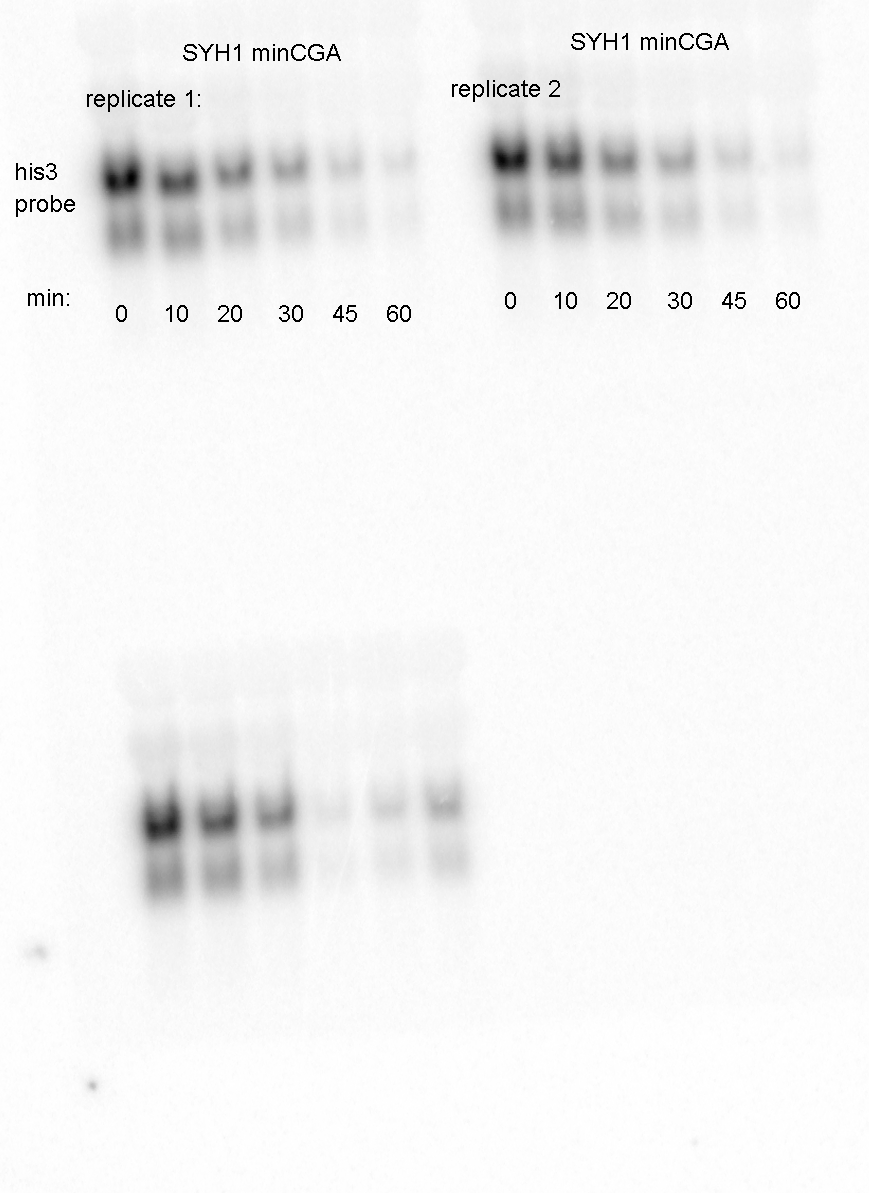

Supplement: Figure 4—source data 2. [file elife-76038-fig4-data2.zip › SYH1/minCGA/annotated_his3_reps_1-2.tif]

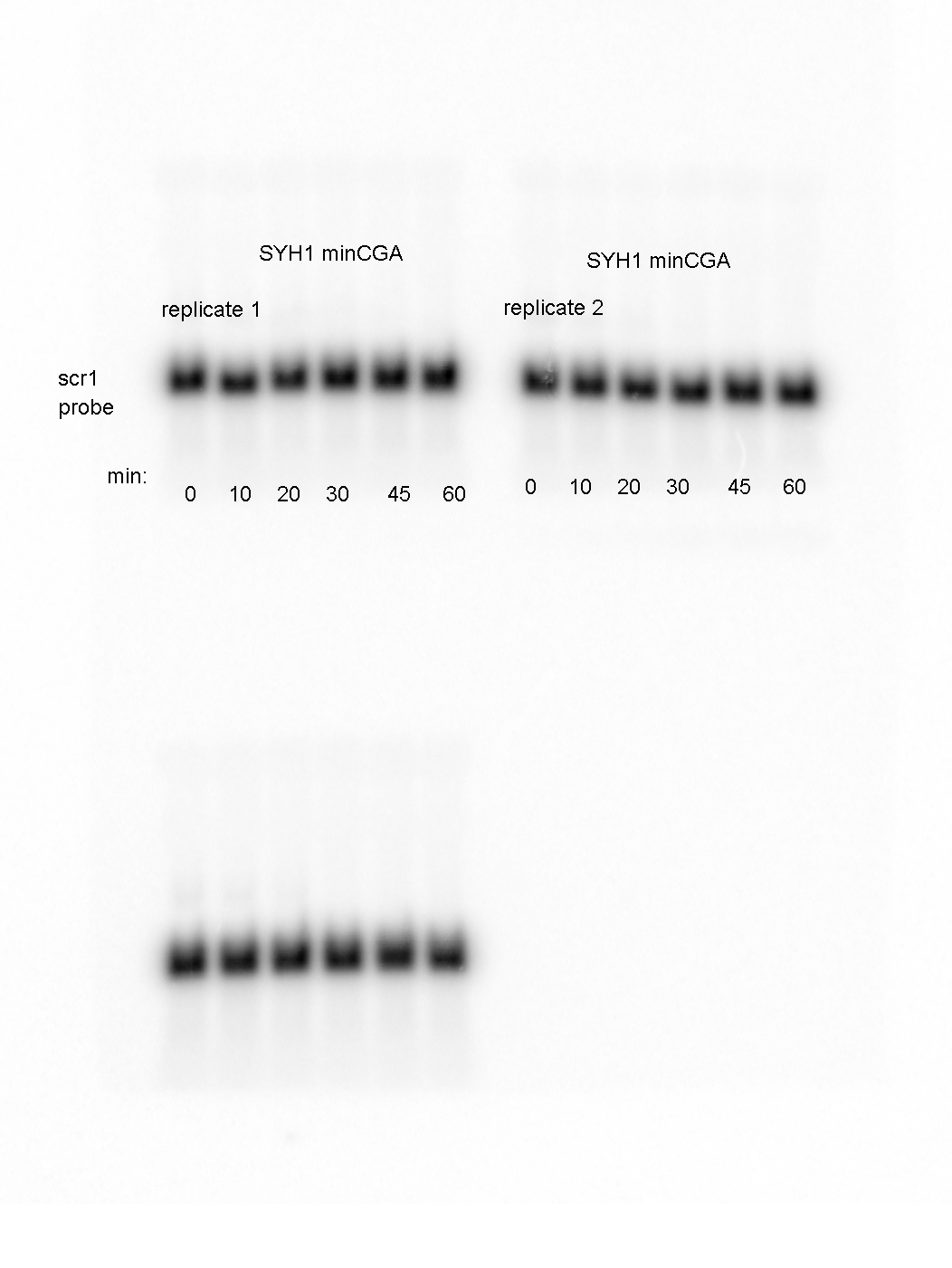

Supplement: Figure 4—source data 2. [file elife-76038-fig4-data2.zip › SYH1/minCGA/annotated_scr1_reps_1-2.tif]

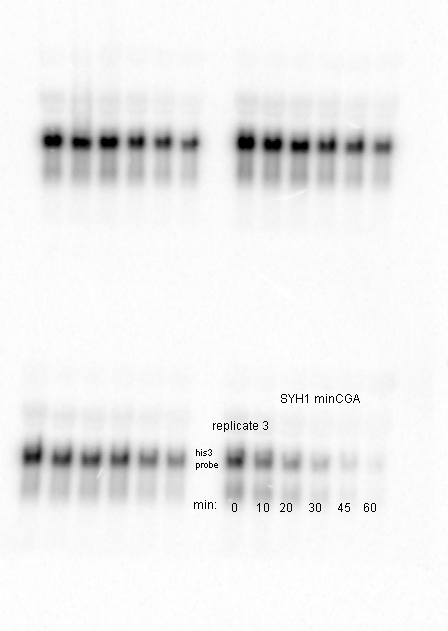

Supplement: Figure 4—source data 2. [file elife-76038-fig4-data2.zip › SYH1/minCGA/annotated_his3_rep_3.tif]

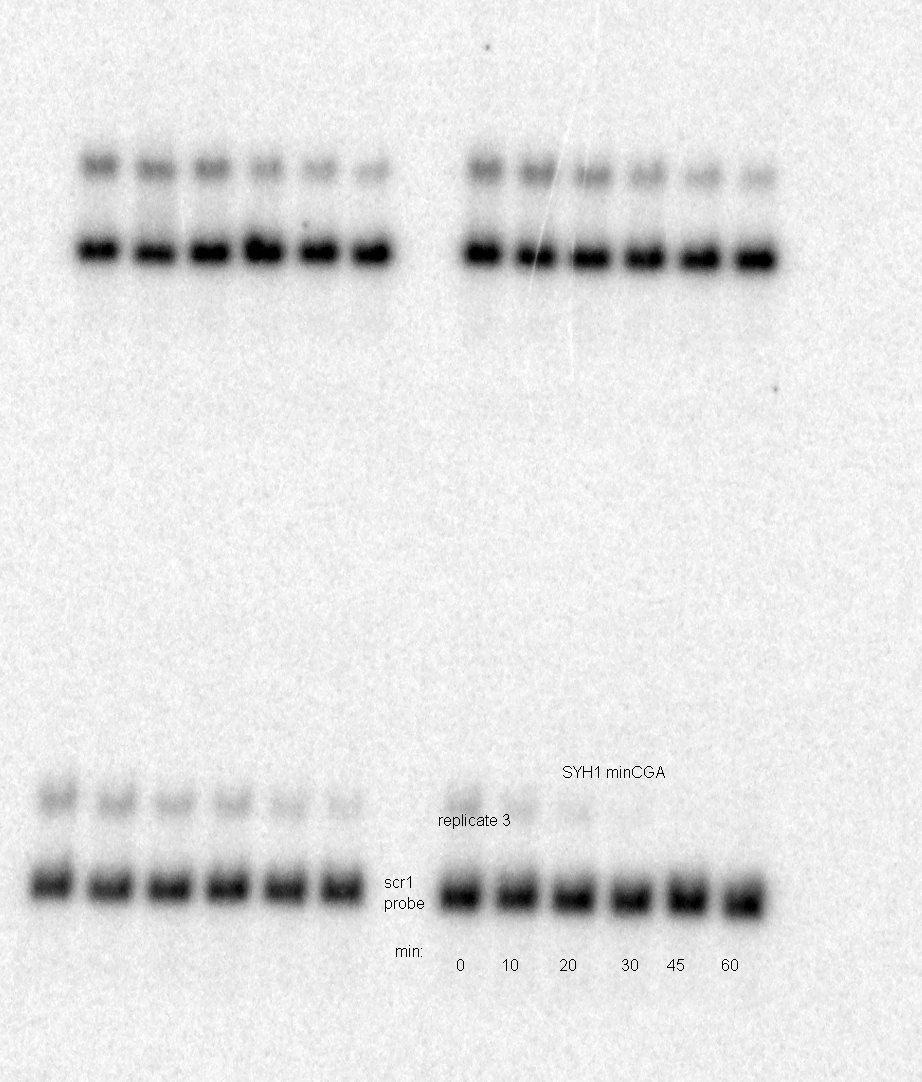

Supplement: Figure 4—source data 2. [file elife-76038-fig4-data2.zip › SYH1/minCGA/annotated_scr1_rep_3.tif]

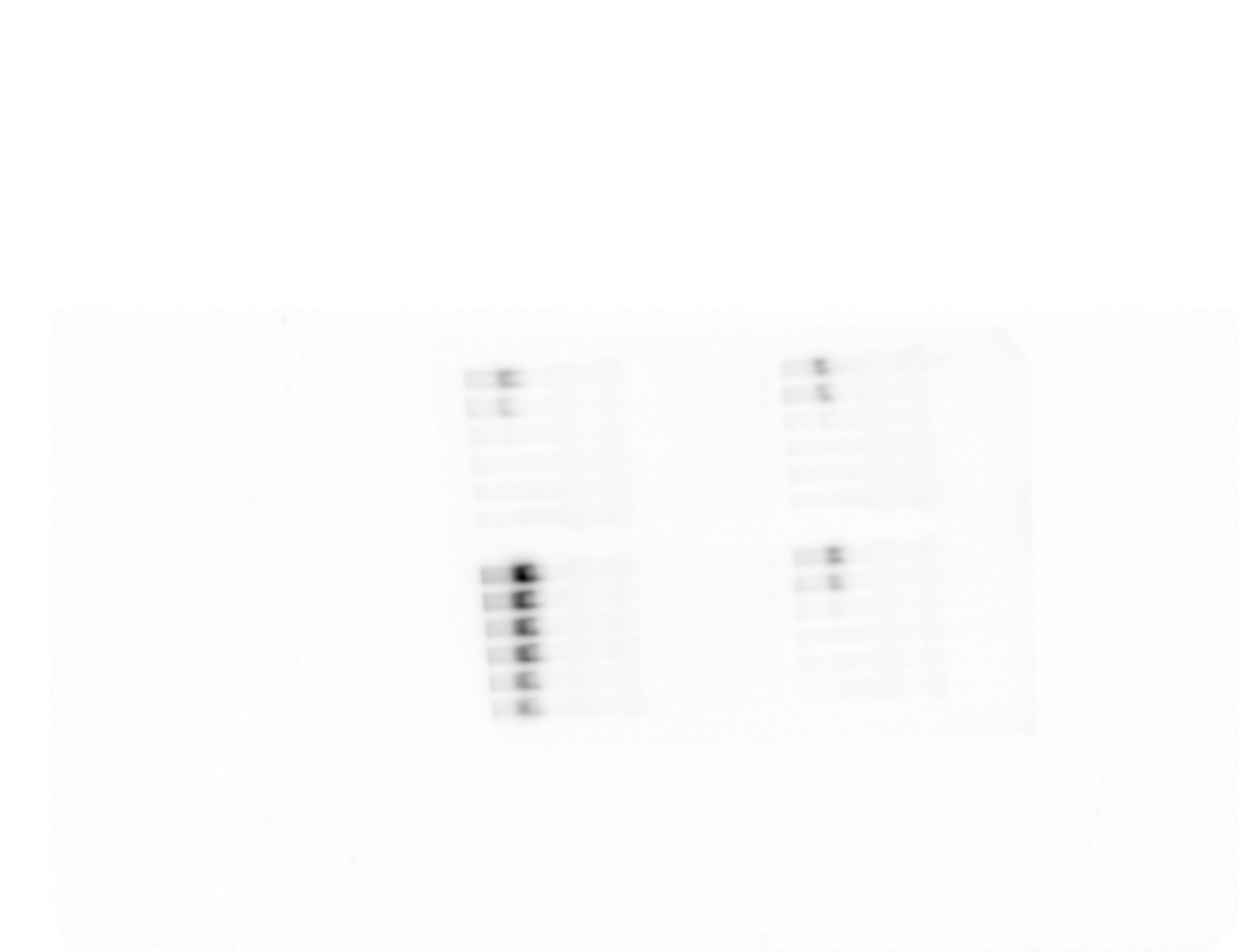

Supplement: Figure 4—source data 3. [file elife-76038-fig4-data3.zip › WT/minNONOPT/raw_image_his3_reps_1-3.gel]

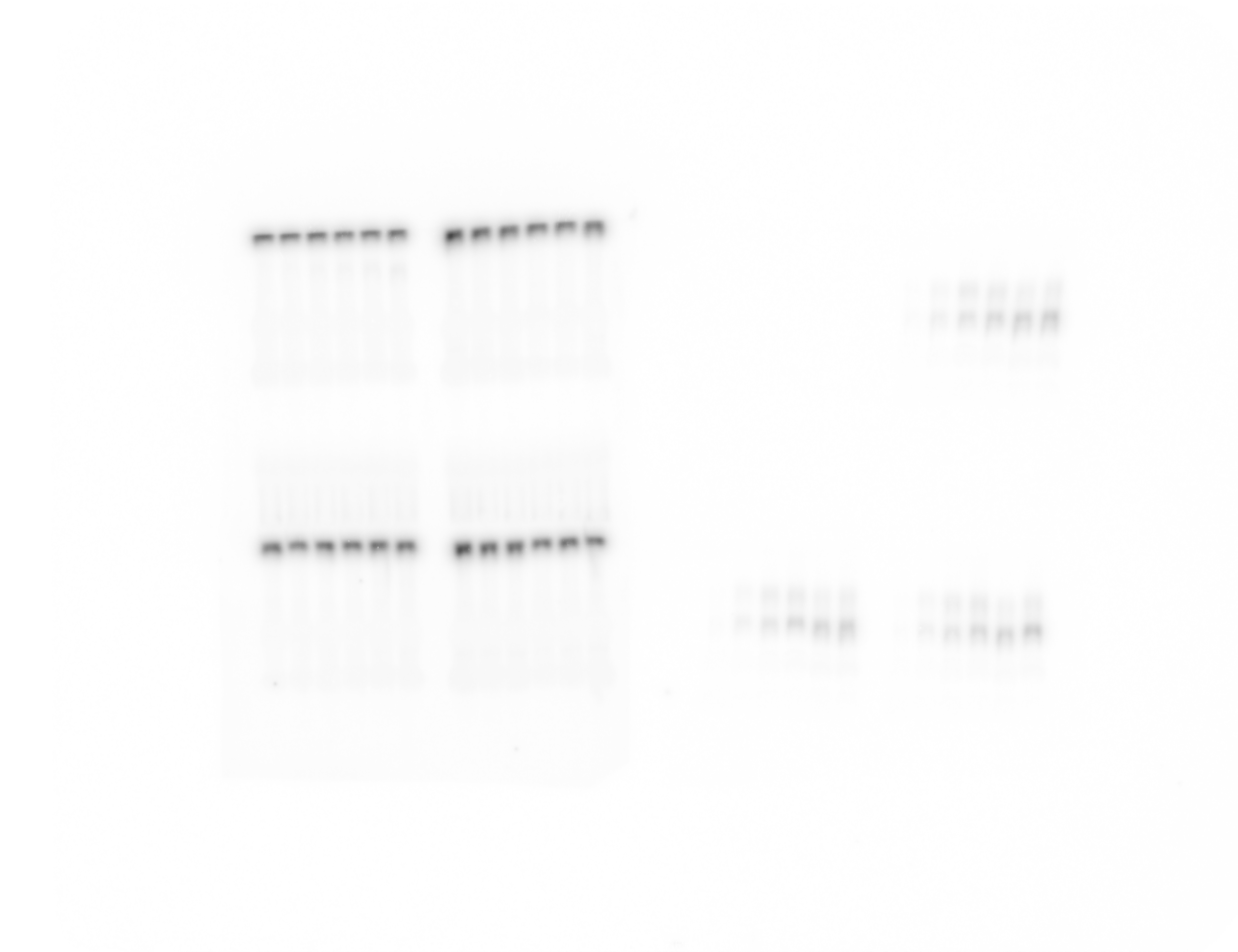

Supplement: Figure 4—source data 3. [file elife-76038-fig4-data3.zip › WT/minNONOPT/raw_image_scr1_reps_1-3.gel]

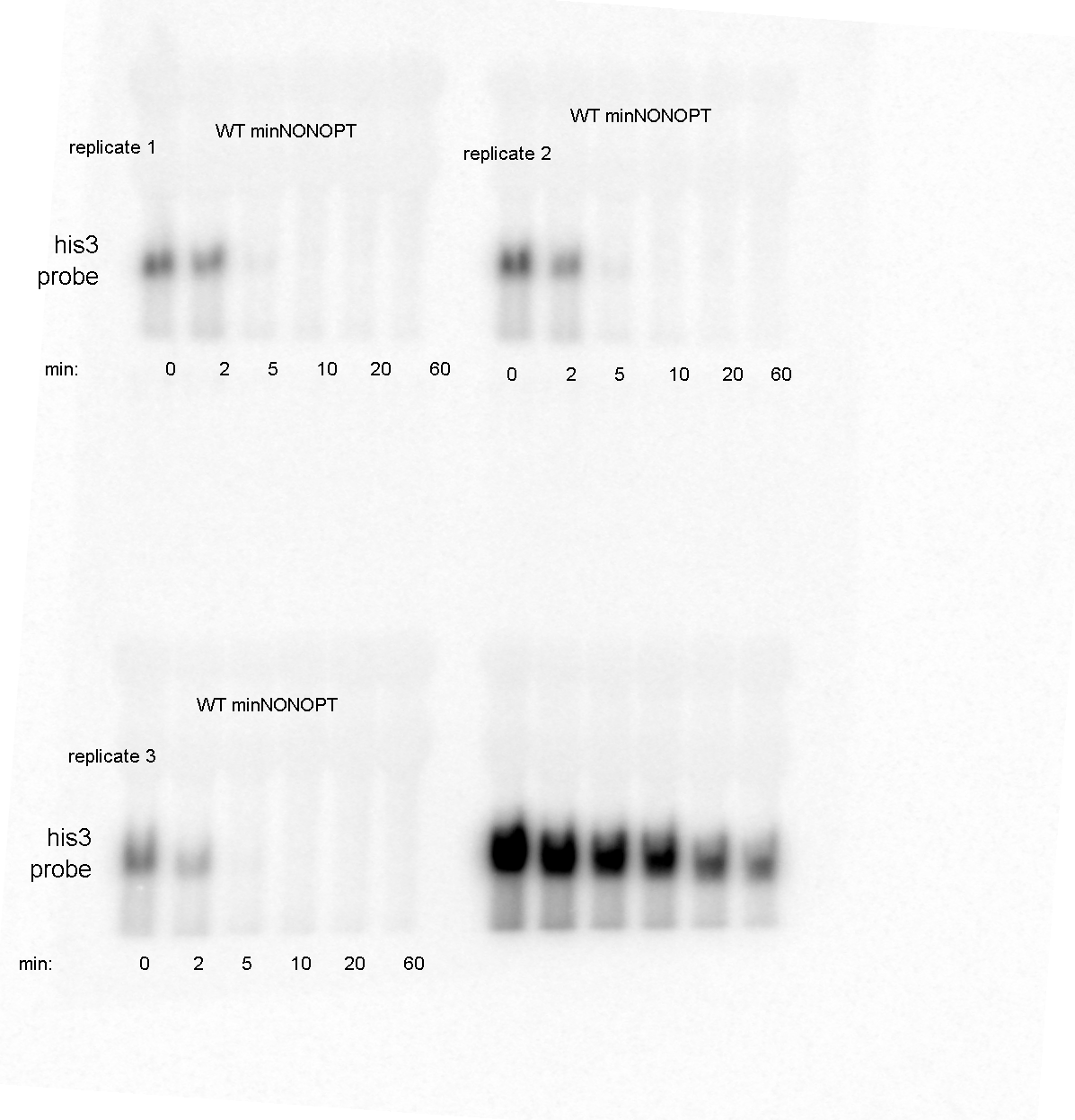

Supplement: Figure 4—source data 3. [file elife-76038-fig4-data3.zip › WT/minNONOPT/annotated_his3_reps_1-3.tif]

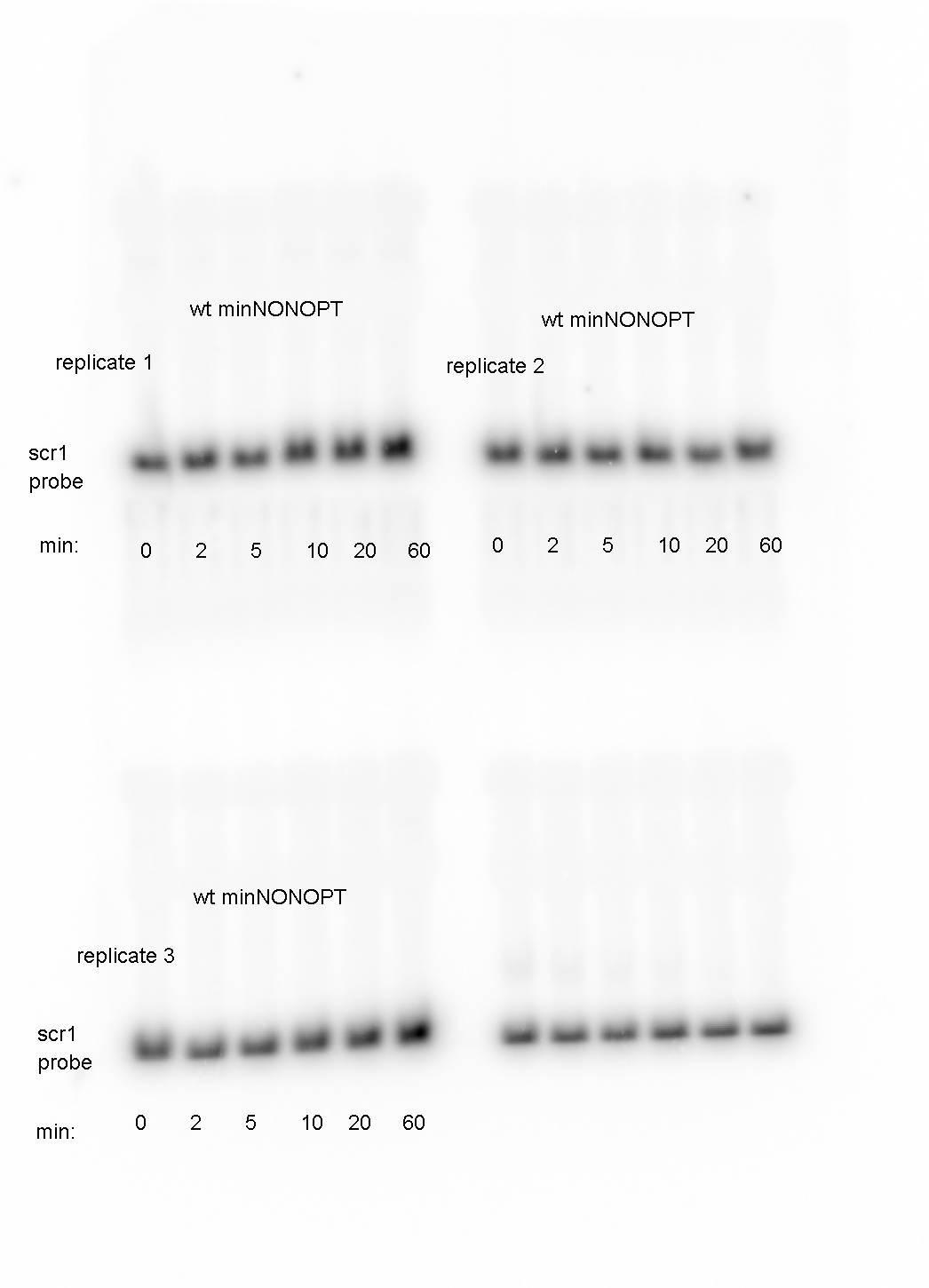

Supplement: Figure 4—source data 3. [file elife-76038-fig4-data3.zip › WT/minNONOPT/annotated_scr1_reps_1-3.tif]

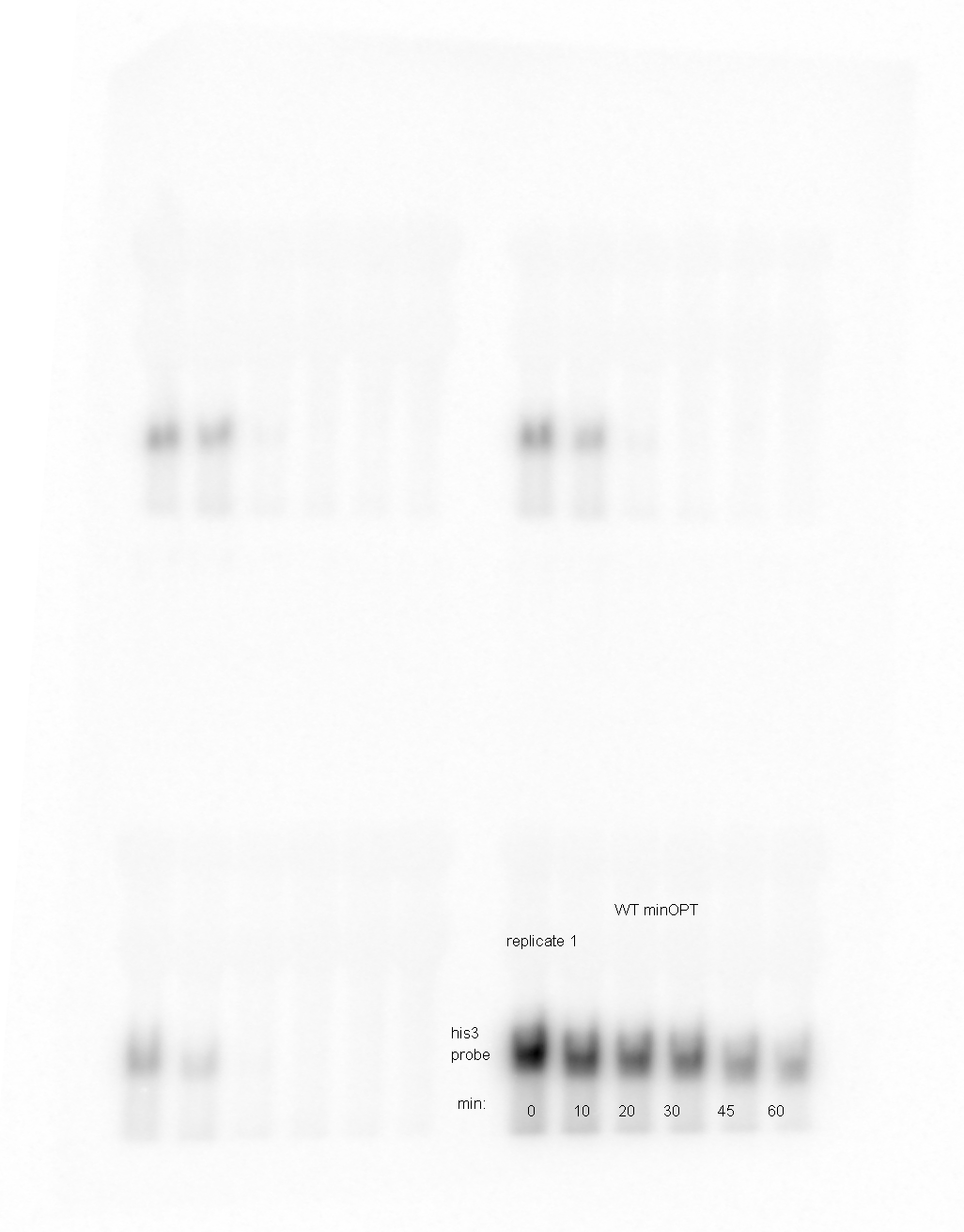

Supplement: Figure 4—source data 3. [file elife-76038-fig4-data3.zip › WT/minOPT/annotated_his3_rep_1.tif]

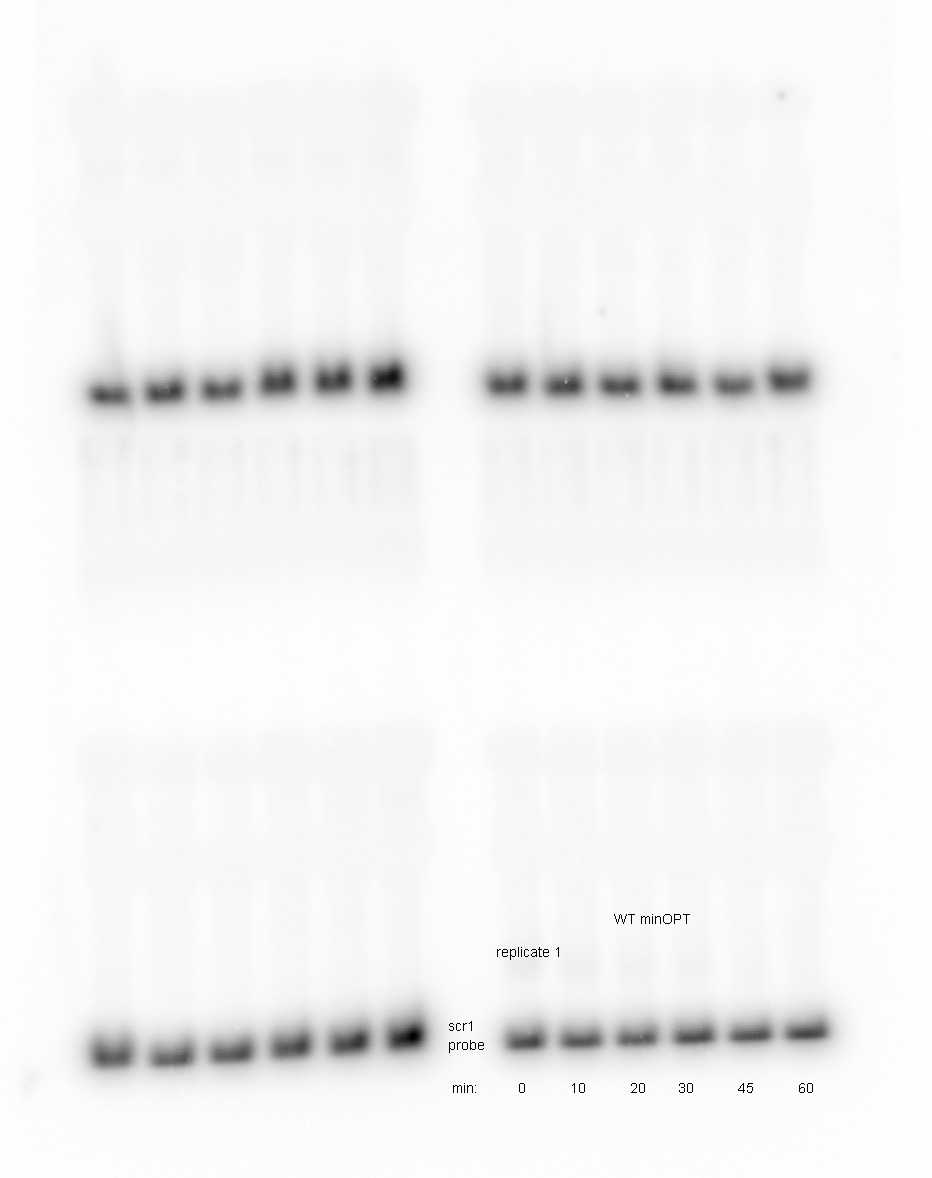

Supplement: Figure 4—source data 3. [file elife-76038-fig4-data3.zip › WT/minOPT/annotated_scr1_rep_1.tif]

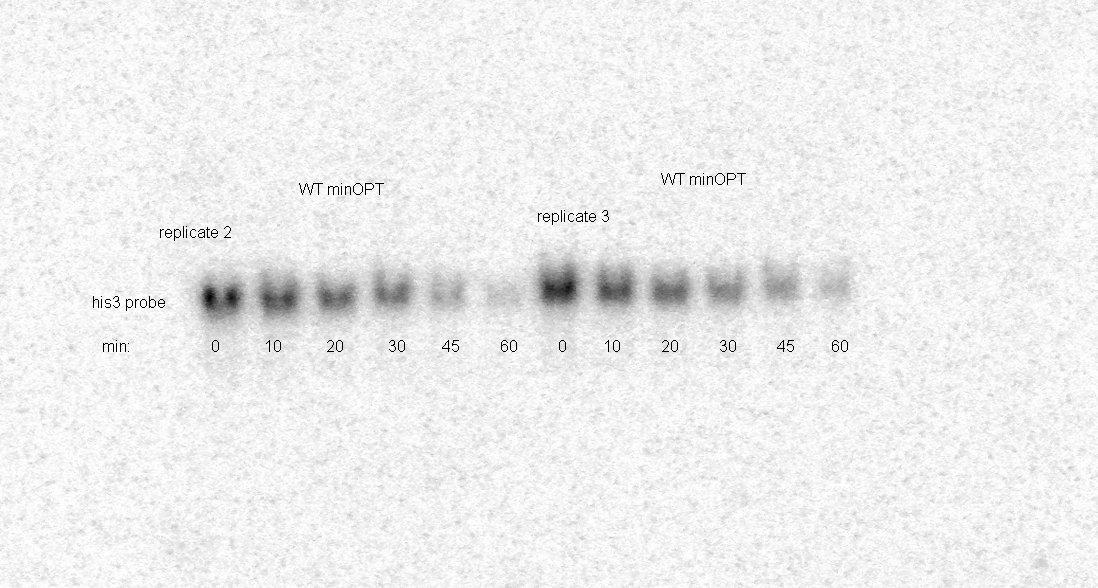

Supplement: Figure 4—source data 3. [file elife-76038-fig4-data3.zip › WT/minOPT/annotated_his3_reps_2-3.tif]

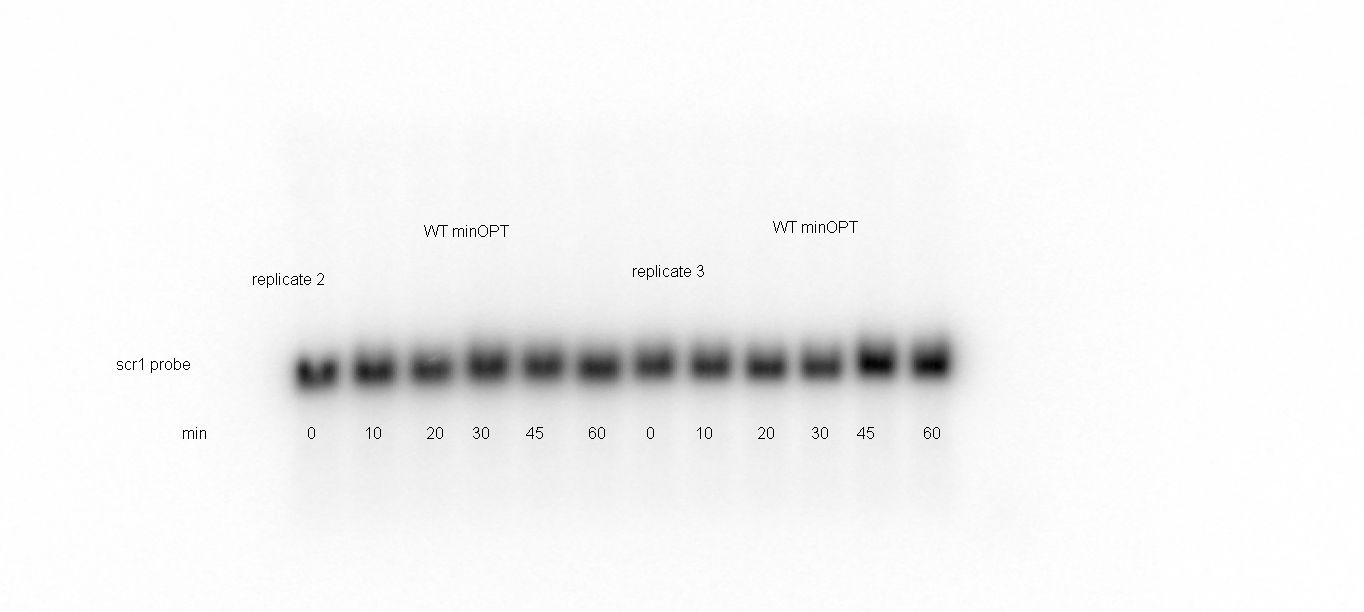

Supplement: Figure 4—source data 3. [file elife-76038-fig4-data3.zip › WT/minOPT/annotated_scr1_reps_2-3.tif]

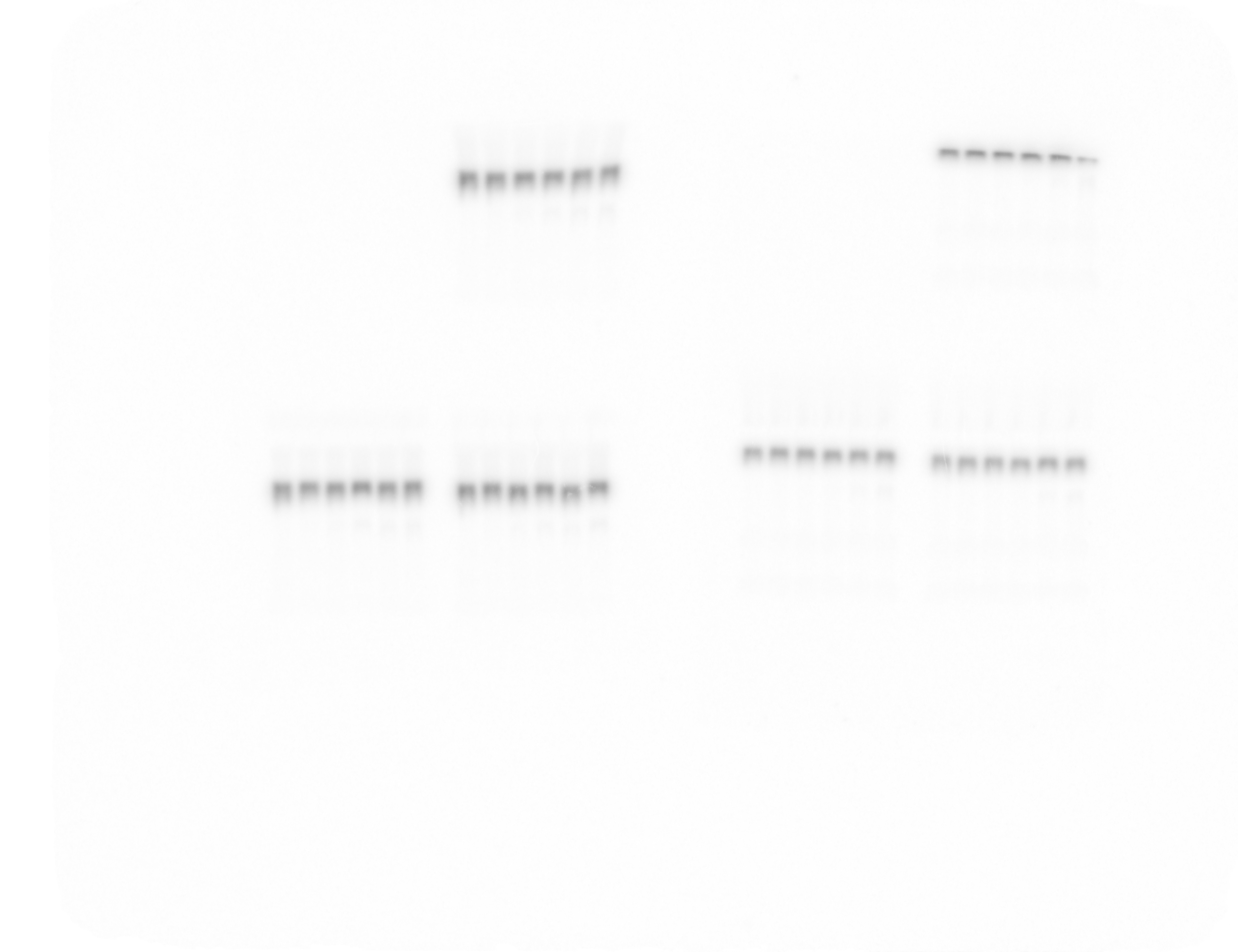

Supplement: Figure 4—source data 3. [file elife-76038-fig4-data3.zip › WT/minCGA/raw_image_scr1_reps_1-3.gel]

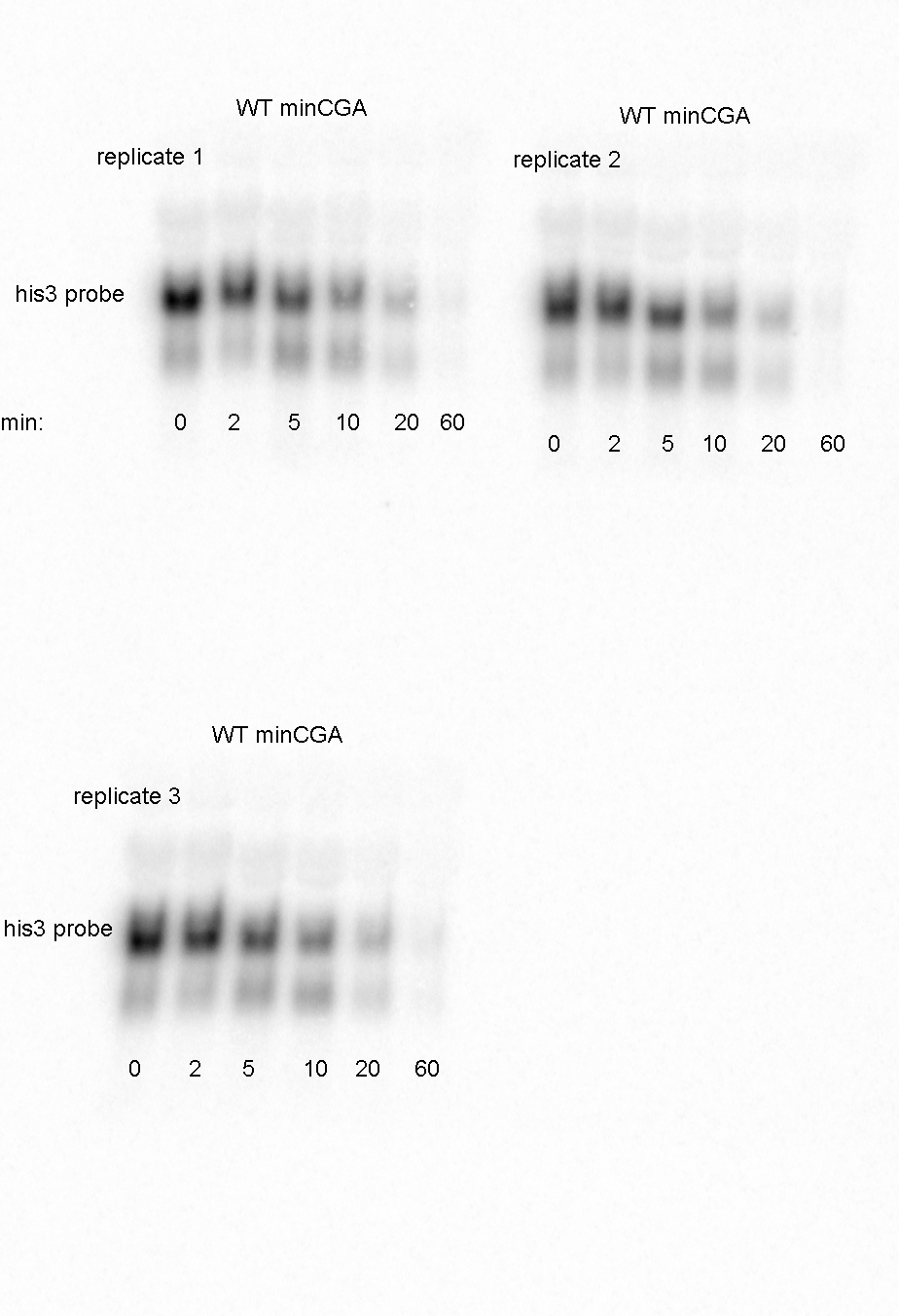

Supplement: Figure 4—source data 3. [file elife-76038-fig4-data3.zip › WT/minCGA/annotated_his3_reps_1-3.tif]

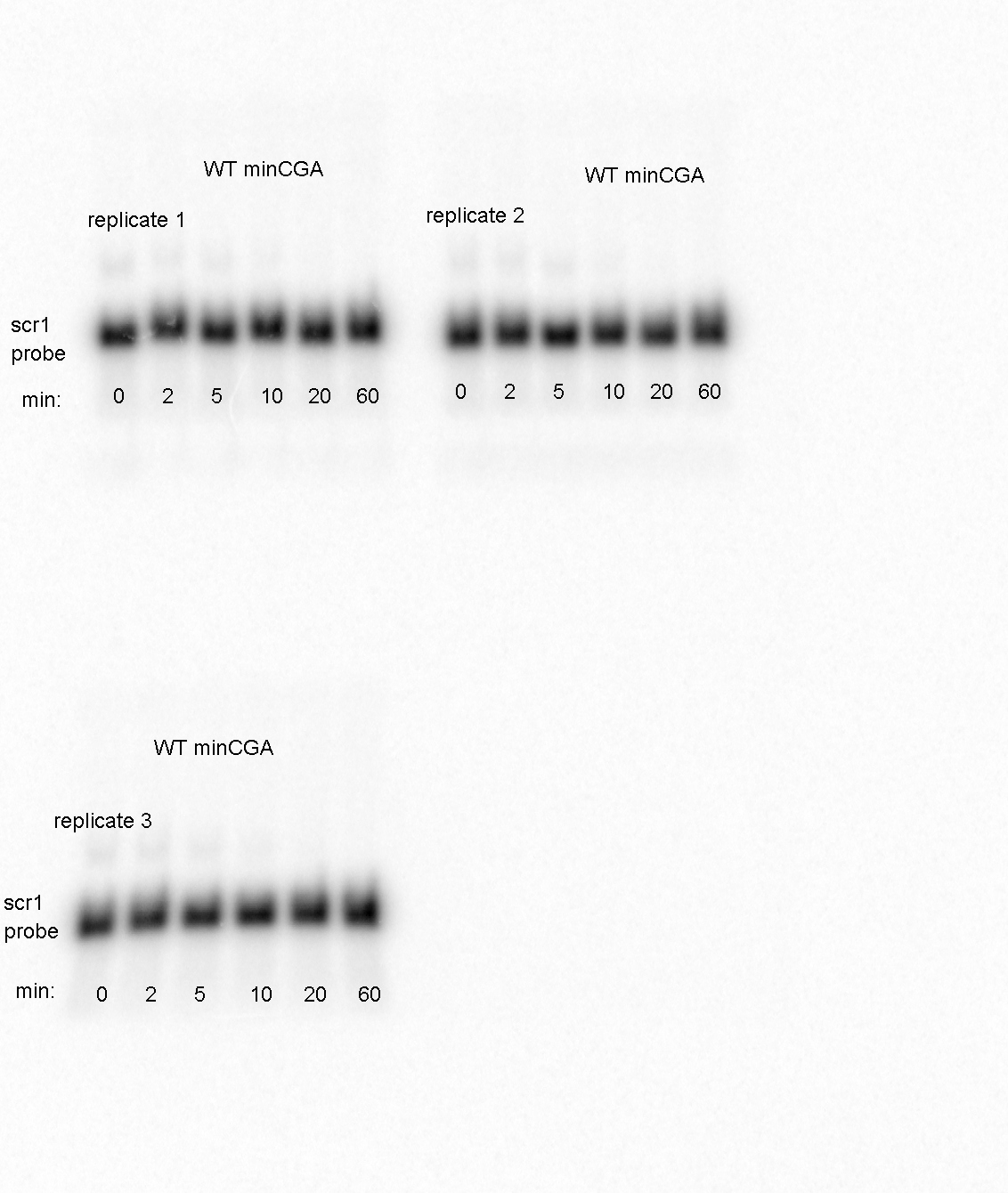

Supplement: Figure 4—source data 3. [file elife-76038-fig4-data3.zip › WT/minCGA/annotated_scr1_reps_1-3.tif]

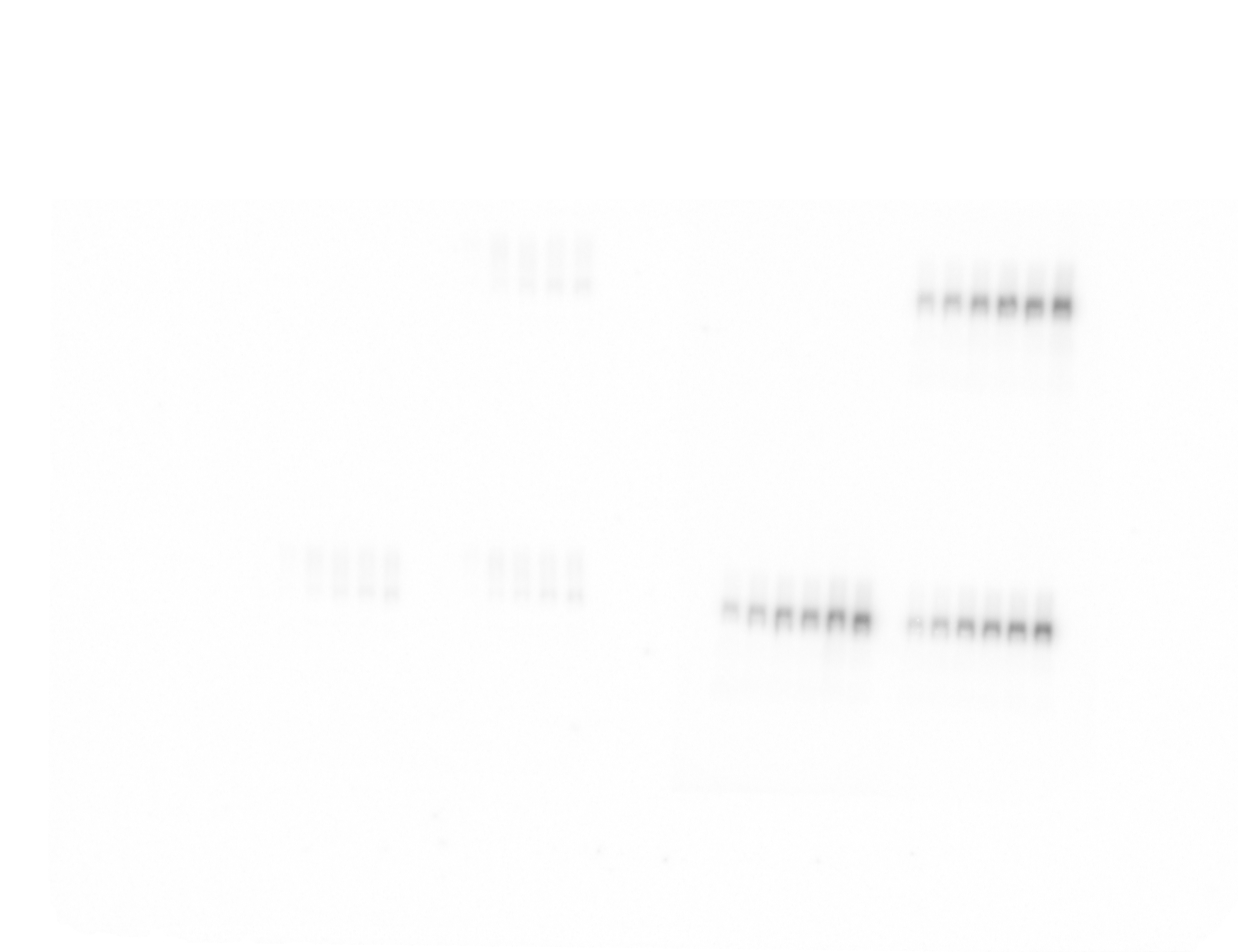

Supplement: Figure 4—source data 4. [file elife-76038-fig4-data4.zip › HEL2/minOPT/raw_image_his3_reps_1-3.gel]

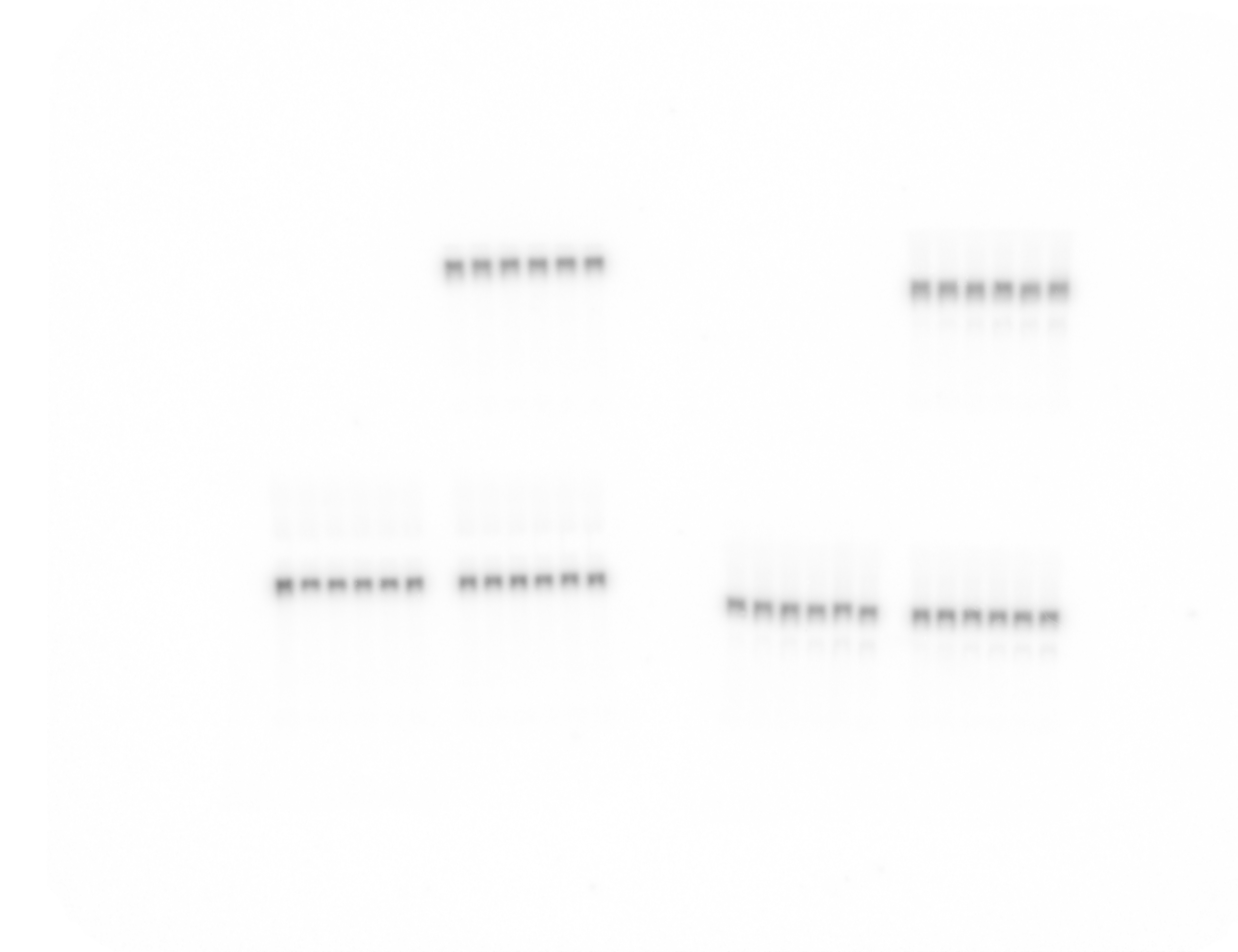

Supplement: Figure 4—source data 4. [file elife-76038-fig4-data4.zip › HEL2/minOPT/raw_image_scr1_reps_1-3.gel]

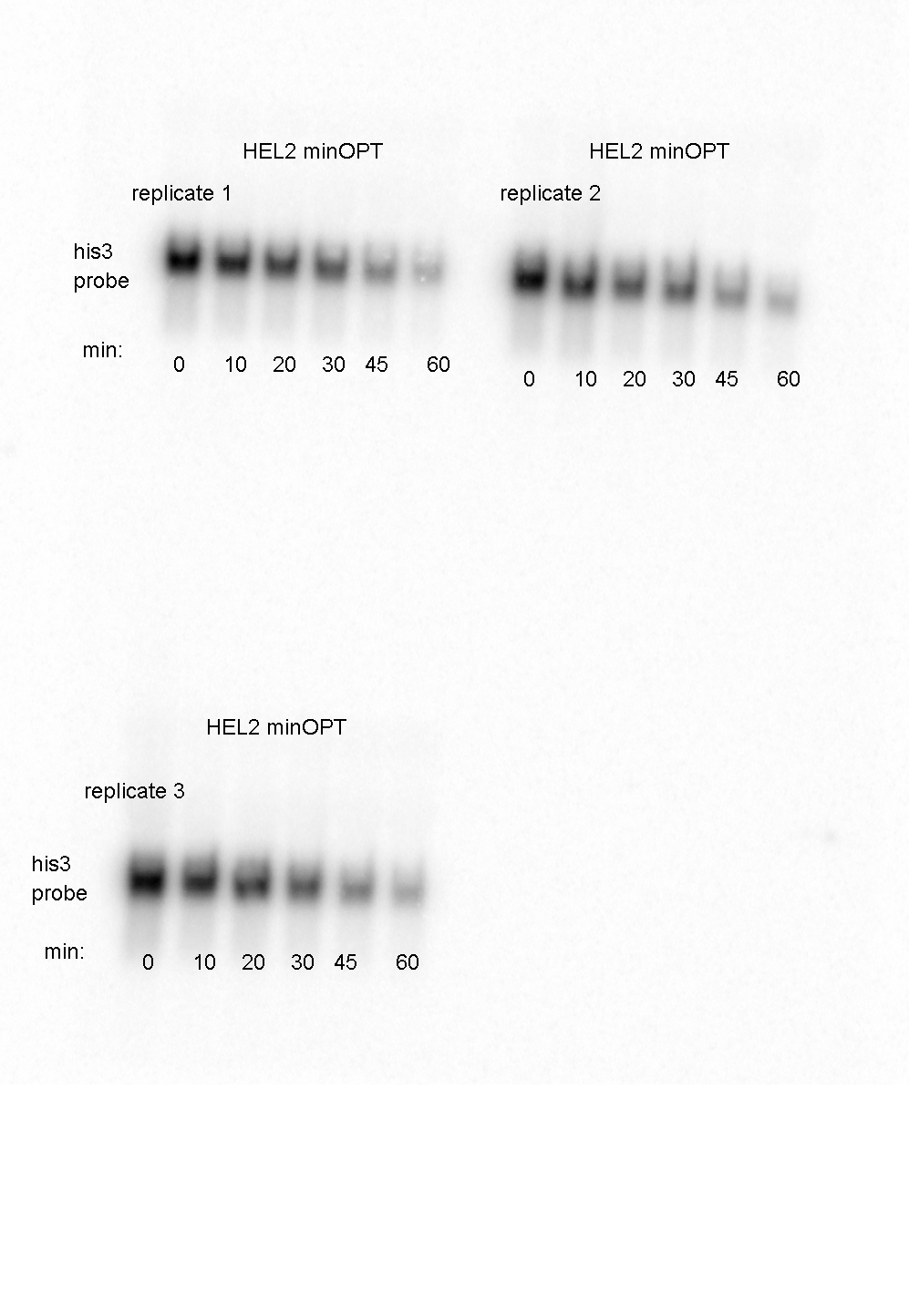

Supplement: Figure 4—source data 4. [file elife-76038-fig4-data4.zip › HEL2/minOPT/annotated_his3_reps_1-3.tif]

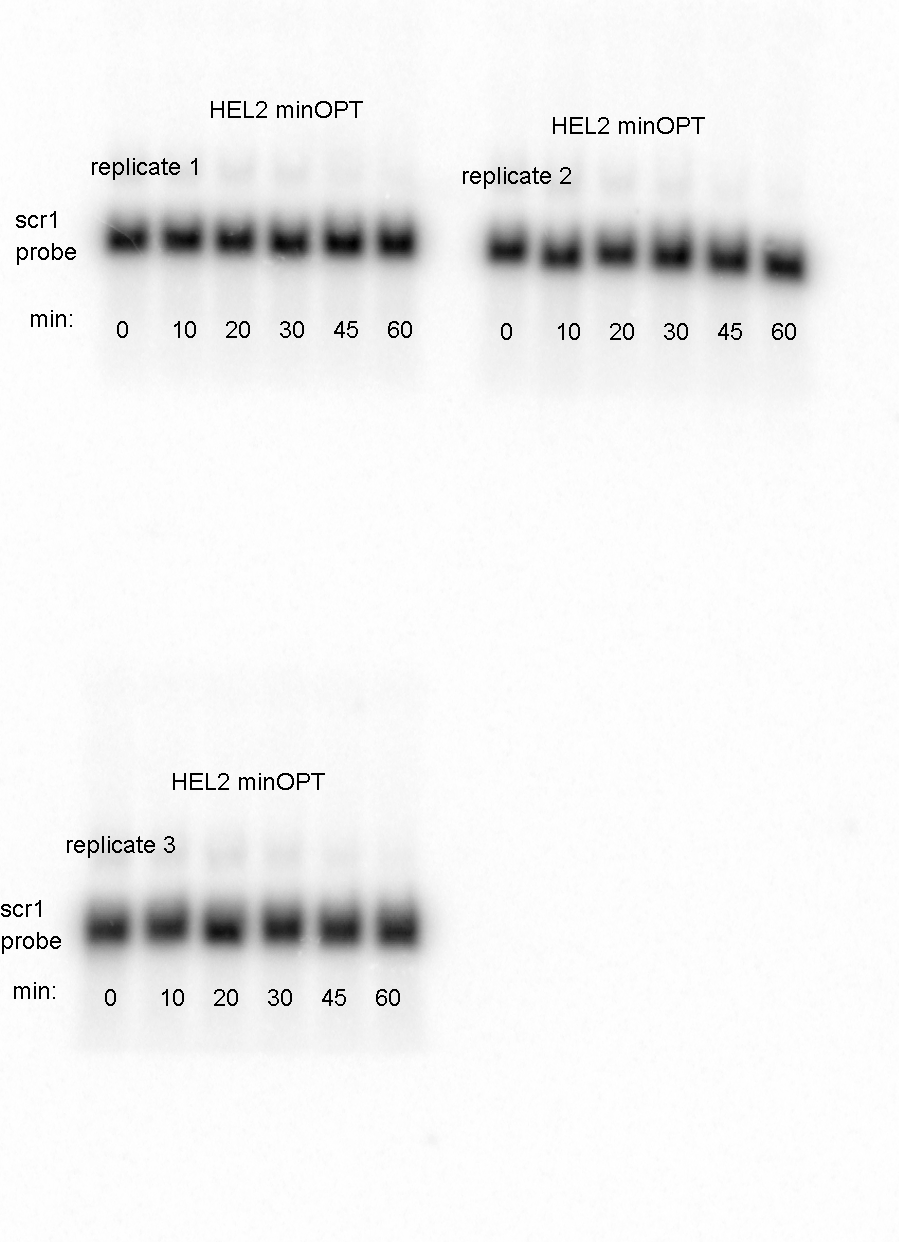

Supplement: Figure 4—source data 4. [file elife-76038-fig4-data4.zip › HEL2/minOPT/annotated_scr1_reps_1-3.tif]

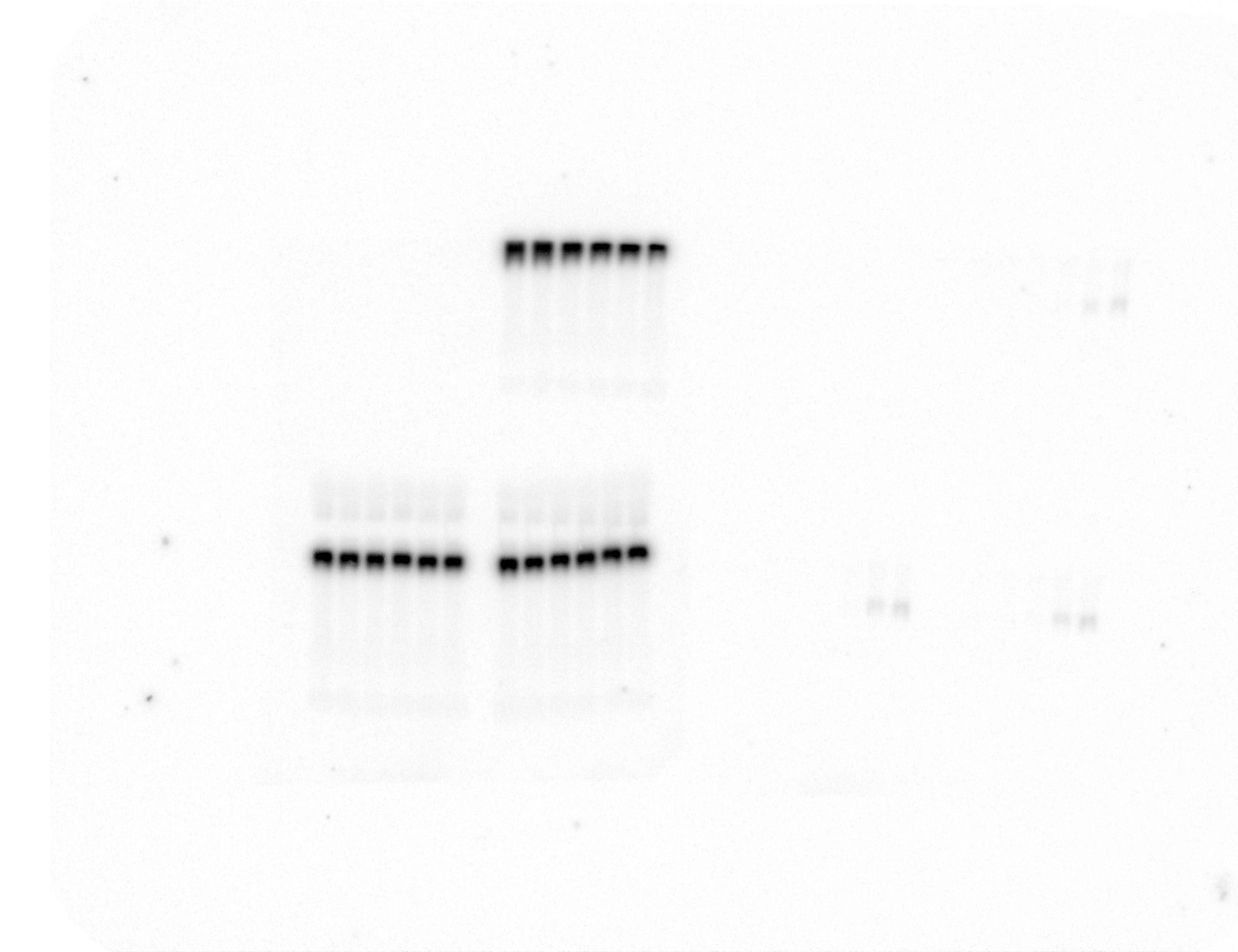

Supplement: Figure 4—source data 4. [file elife-76038-fig4-data4.zip › HEL2/minNONOPT/raw_image_his3_reps_1-3.gel]

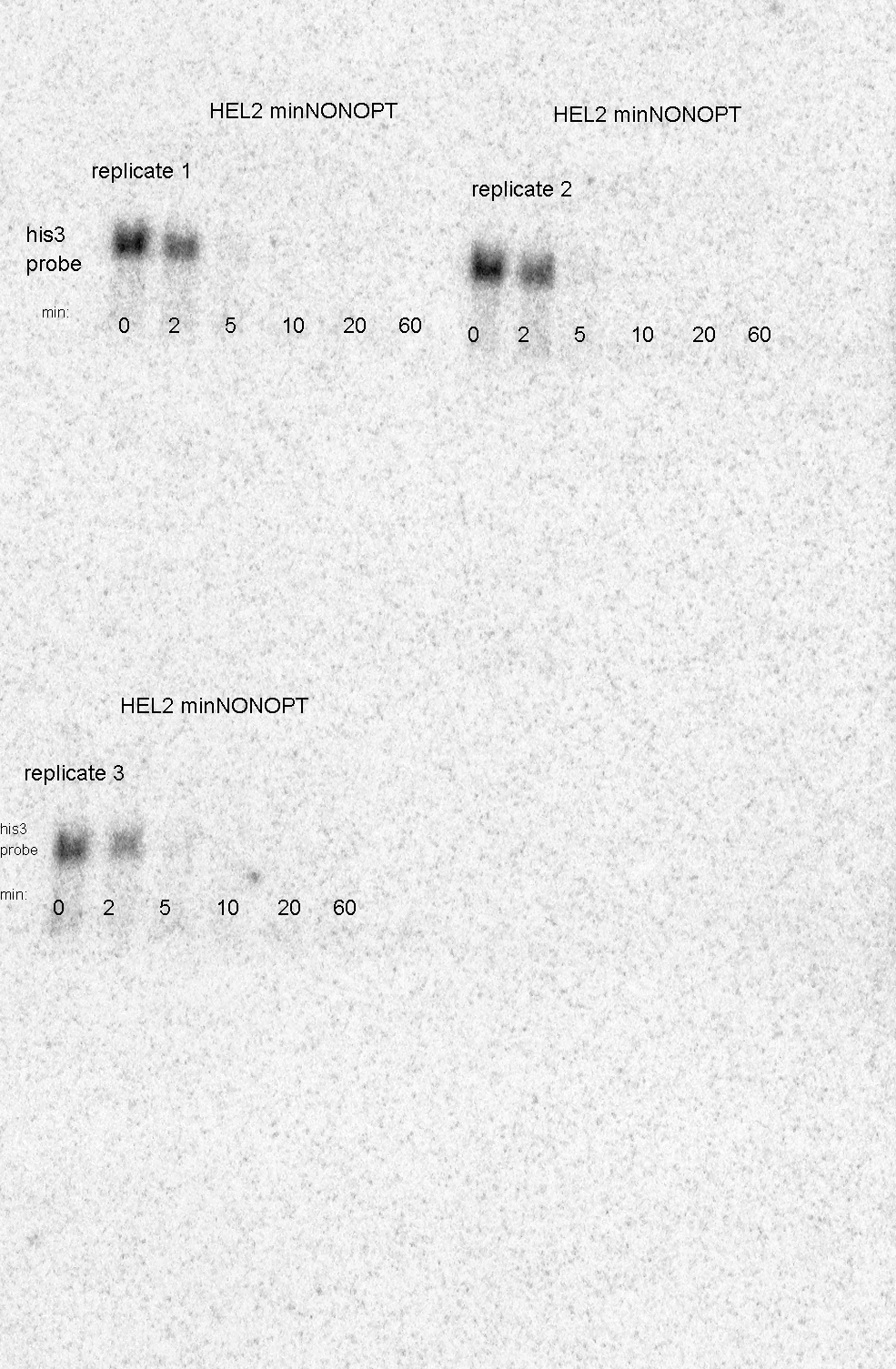

Supplement: Figure 4—source data 4. [file elife-76038-fig4-data4.zip › HEL2/minNONOPT/annotated_his3_reps_1-3.tif]

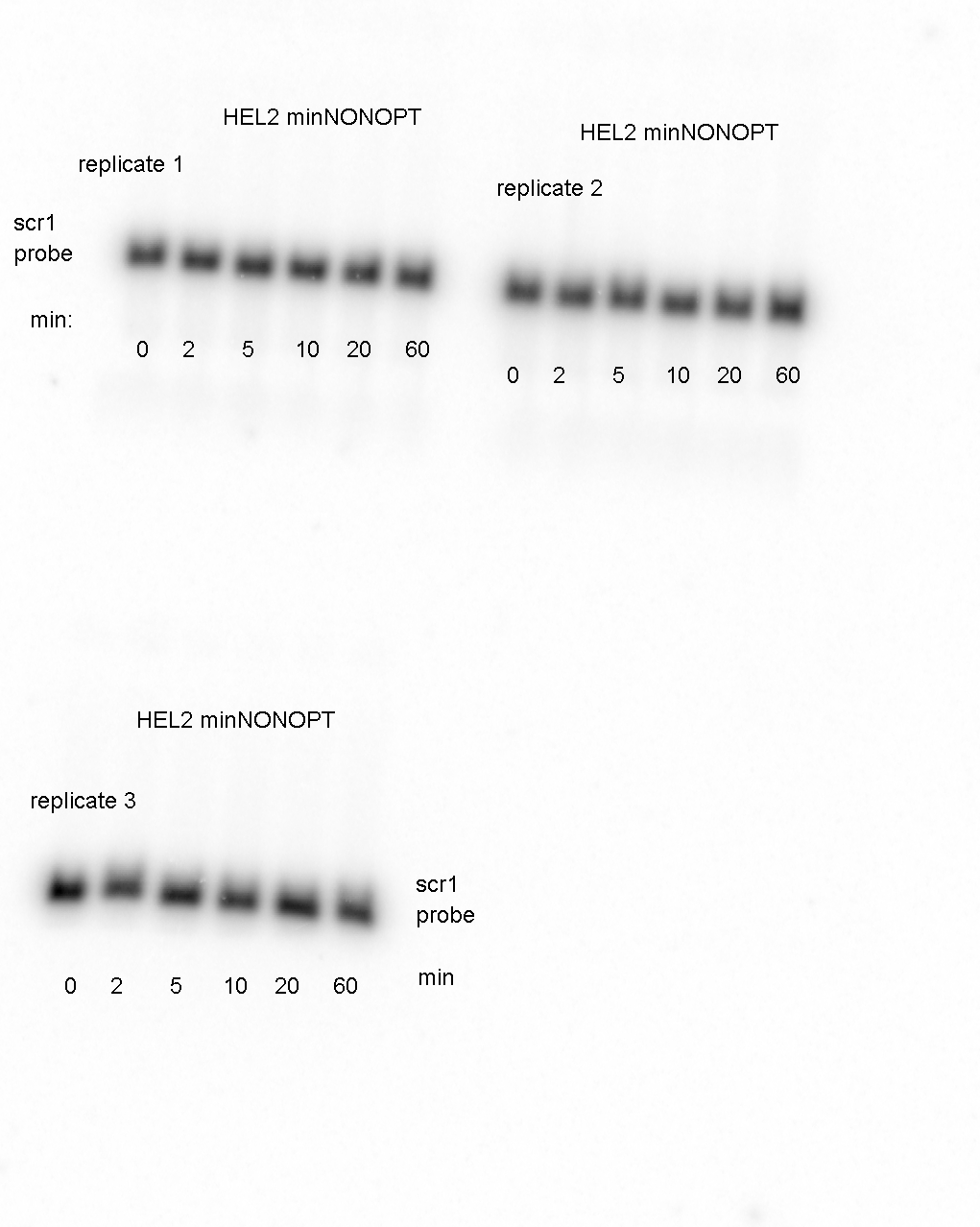

Supplement: Figure 4—source data 4. [file elife-76038-fig4-data4.zip › HEL2/minNONOPT/annotated_scr1_reps_1-3.tif]

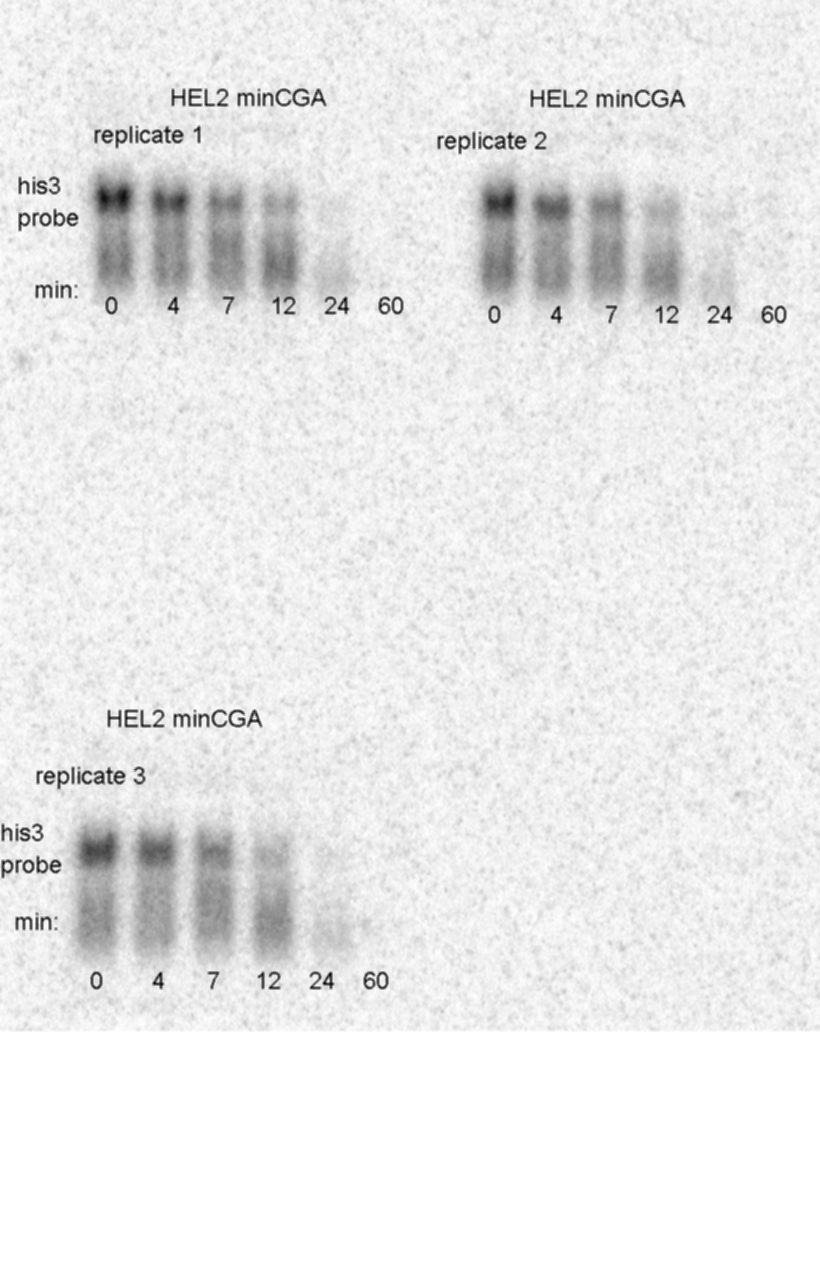

Supplement: Figure 4—source data 4. [file elife-76038-fig4-data4.zip › HEL2/minCGA/annotated_his3_reps_1-3.tif]

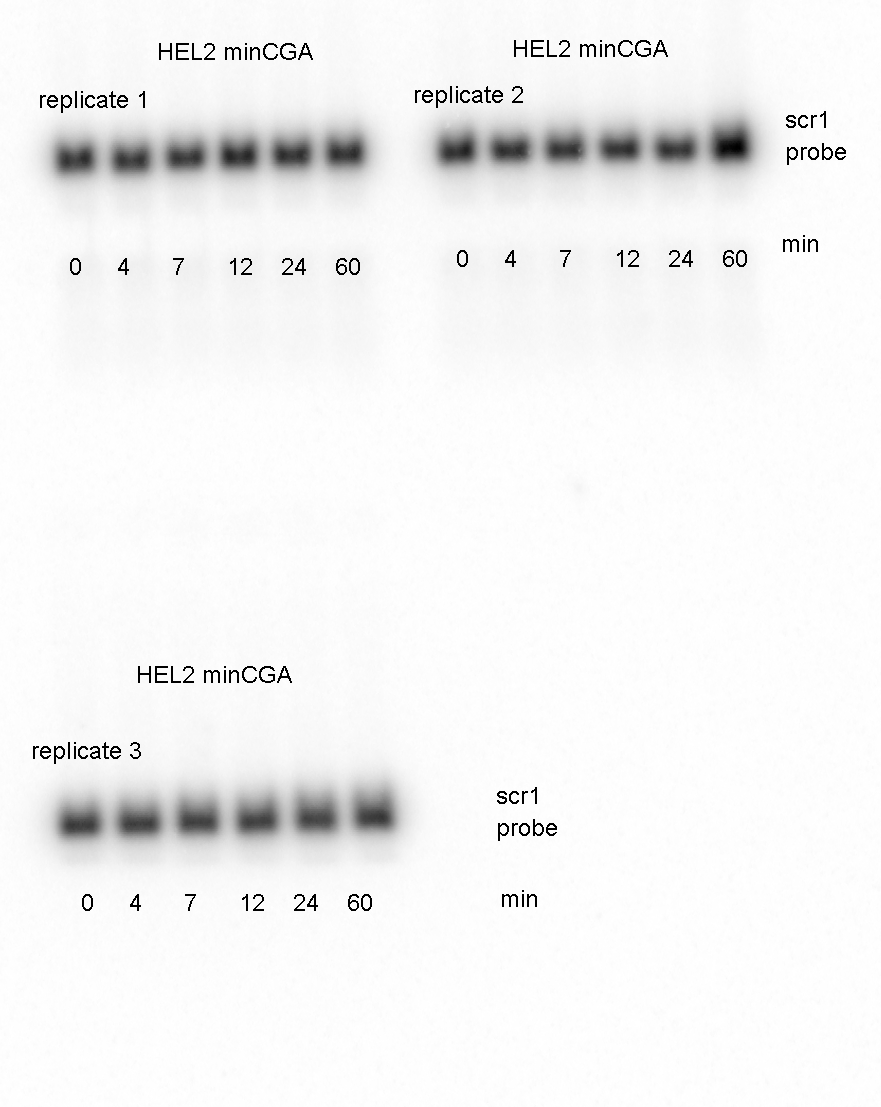

Supplement: Figure 4—source data 4. [file elife-76038-fig4-data4.zip › HEL2/minCGA/annotated_scr1_reps_1-3.tif]

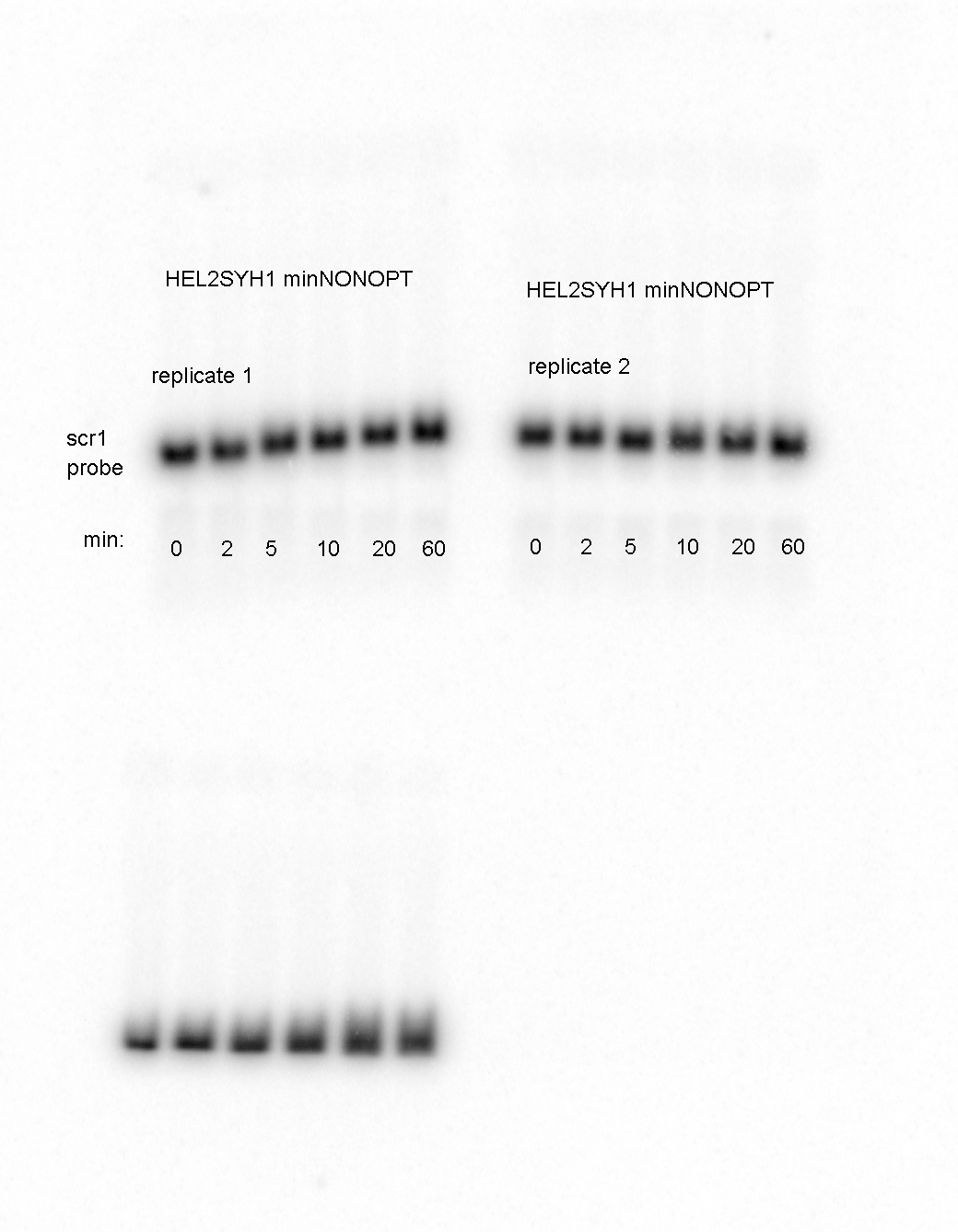

Supplement: Figure 4—source data 5. [file elife-76038-fig4-data5.zip › HEL2SYH1/minNONOPT/annotated_scr1_reps_1-2.tif]

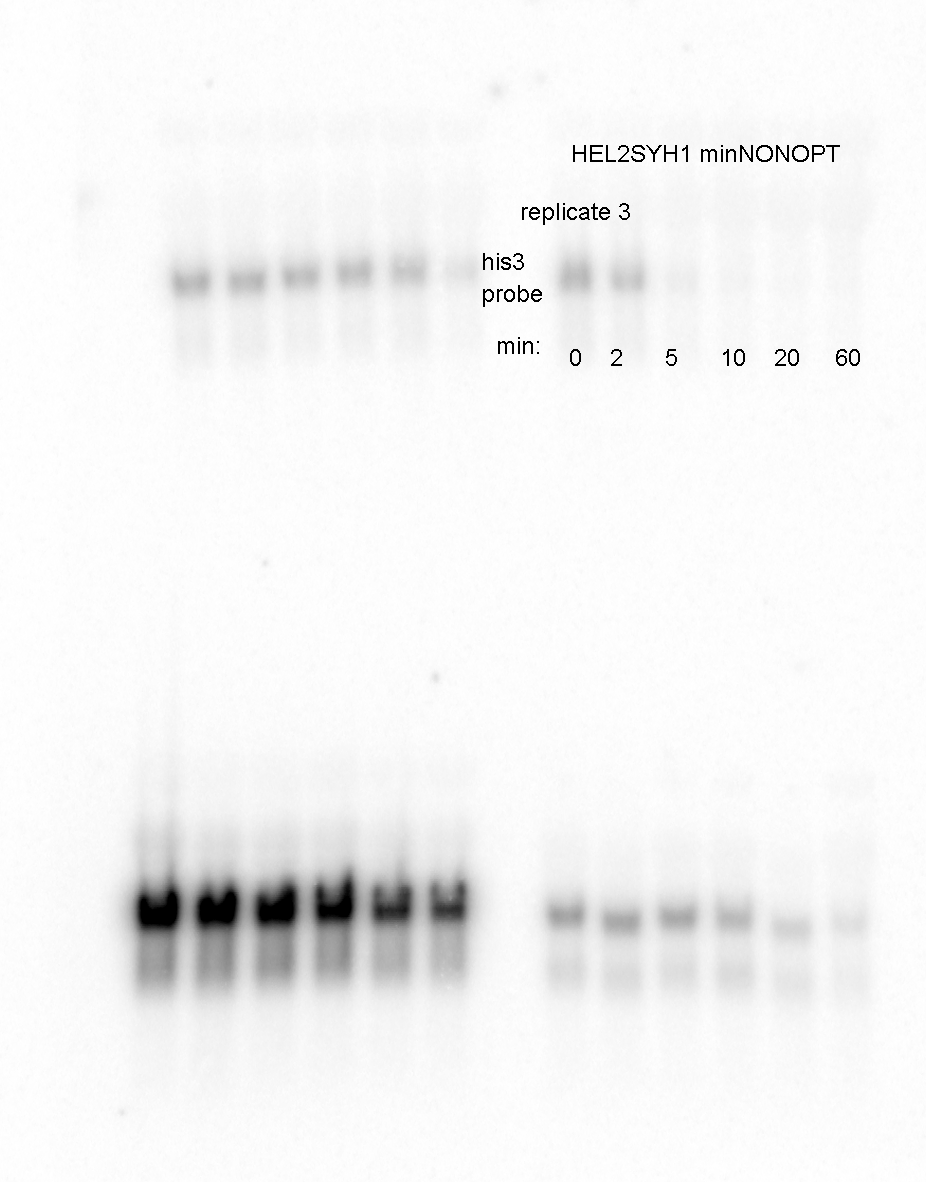

Supplement: Figure 4—source data 5. [file elife-76038-fig4-data5.zip › HEL2SYH1/minNONOPT/annotated_his3_rep_3.tif]

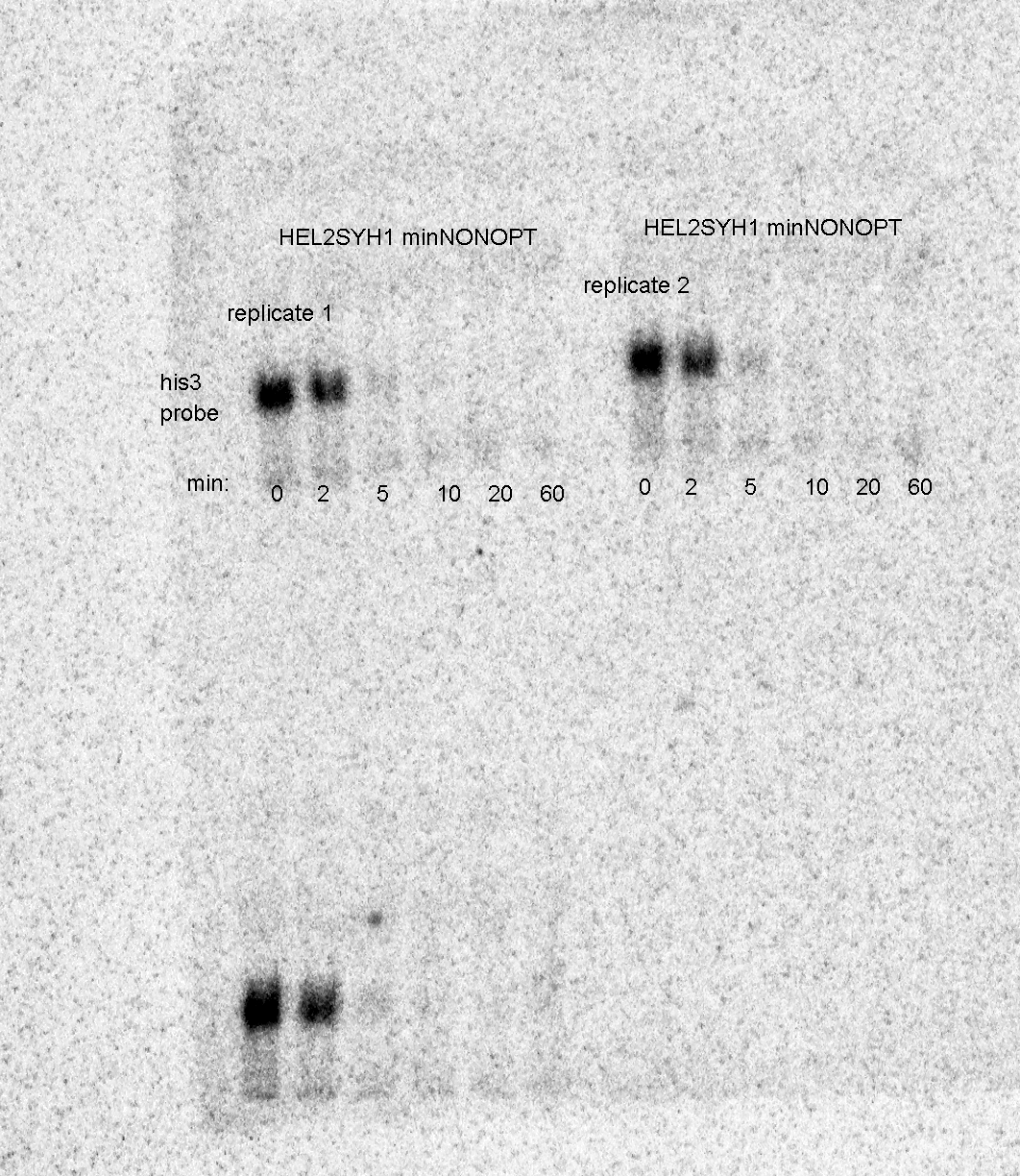

Supplement: Figure 4—source data 5. [file elife-76038-fig4-data5.zip › HEL2SYH1/minNONOPT/annotated_his3_reps_1-2.tif]

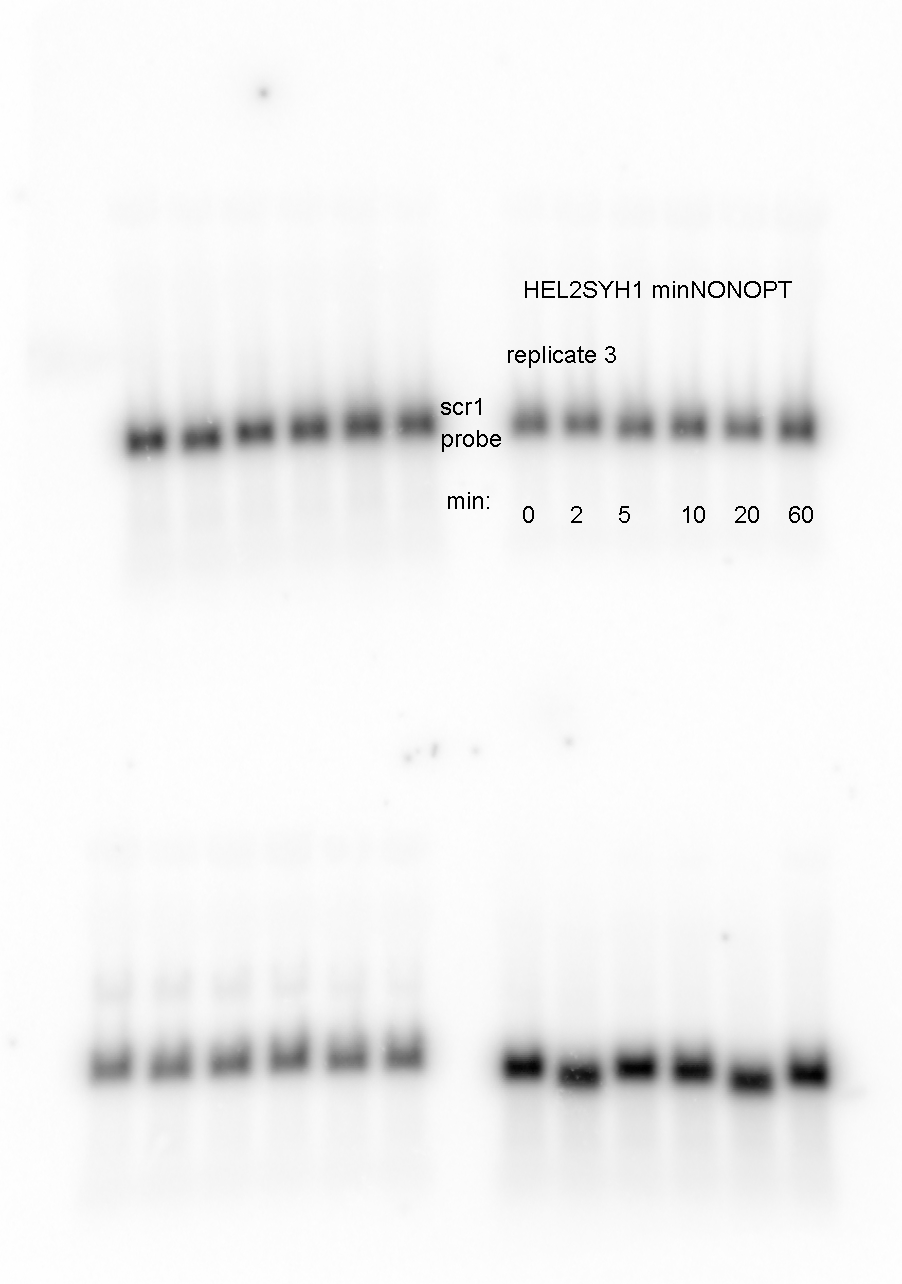

Supplement: Figure 4—source data 5. [file elife-76038-fig4-data5.zip › HEL2SYH1/minNONOPT/annotated_scr1_rep_3.tif]

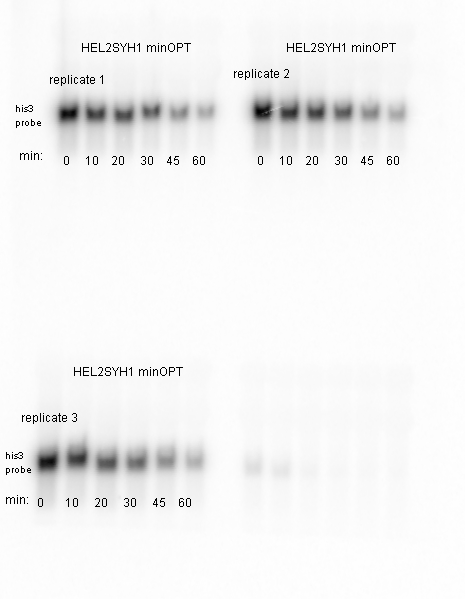

Supplement: Figure 4—source data 5. [file elife-76038-fig4-data5.zip › HEL2SYH1/minOPT/annotated_his3_reps_1-3.tif]

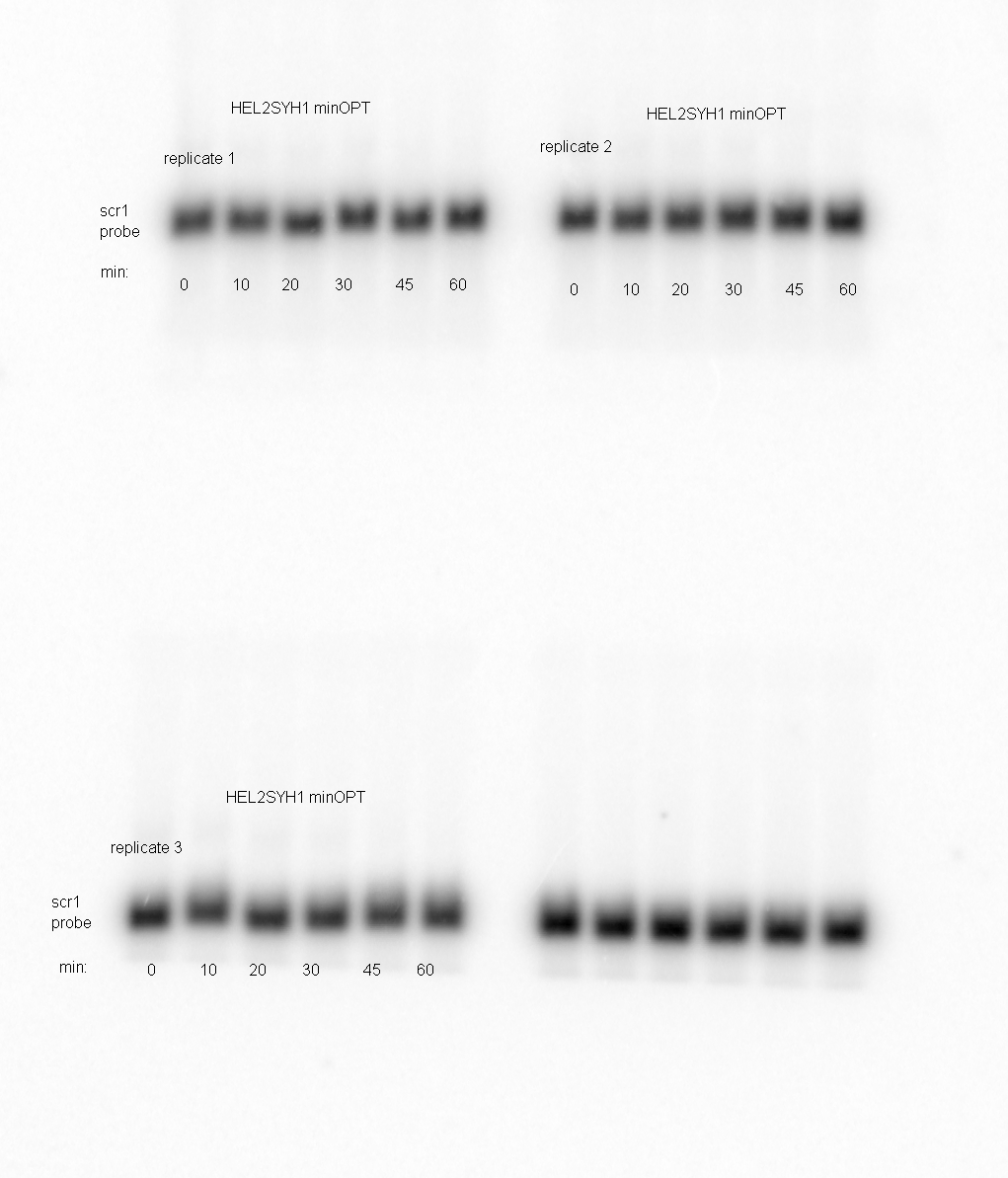

Supplement: Figure 4—source data 5. [file elife-76038-fig4-data5.zip › HEL2SYH1/minOPT/annotated_scr1_reps_1-3.tif]

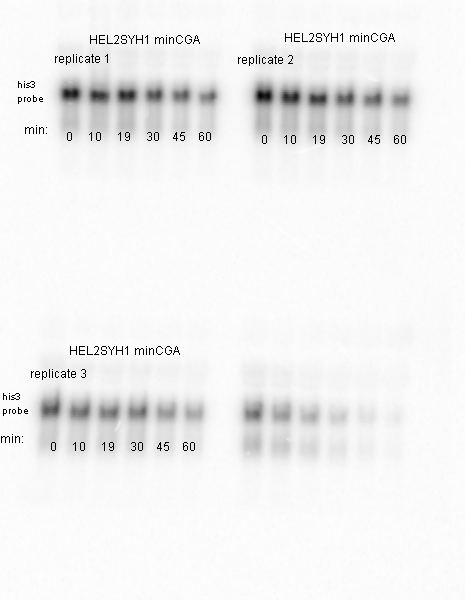

Supplement: Figure 4—source data 5. [file elife-76038-fig4-data5.zip › HEL2SYH1/minCGA/annotated_his3_reps_1-3.tif]

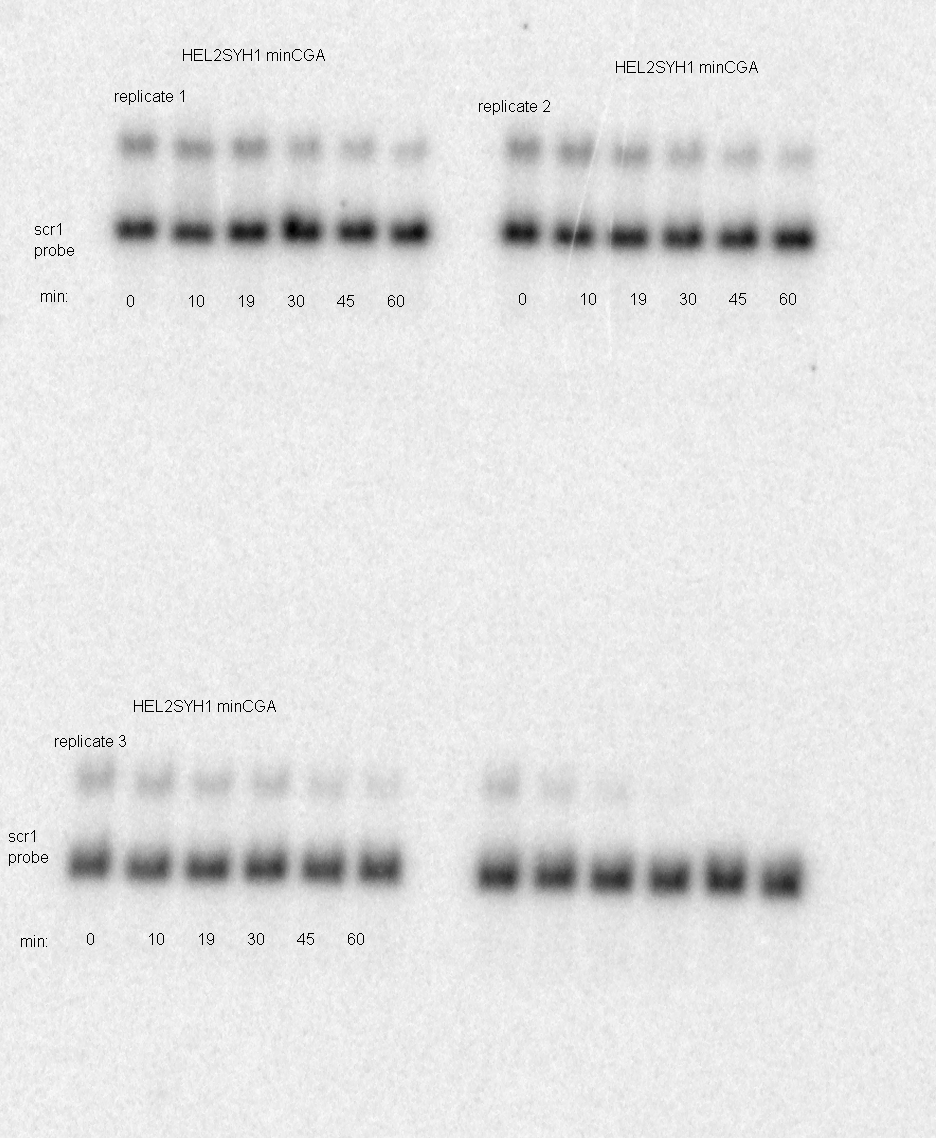

Supplement: Figure 4—source data 5. [file elife-76038-fig4-data5.zip › HEL2SYH1/minCGA/annotated_scr1_reps_1-3.tif]

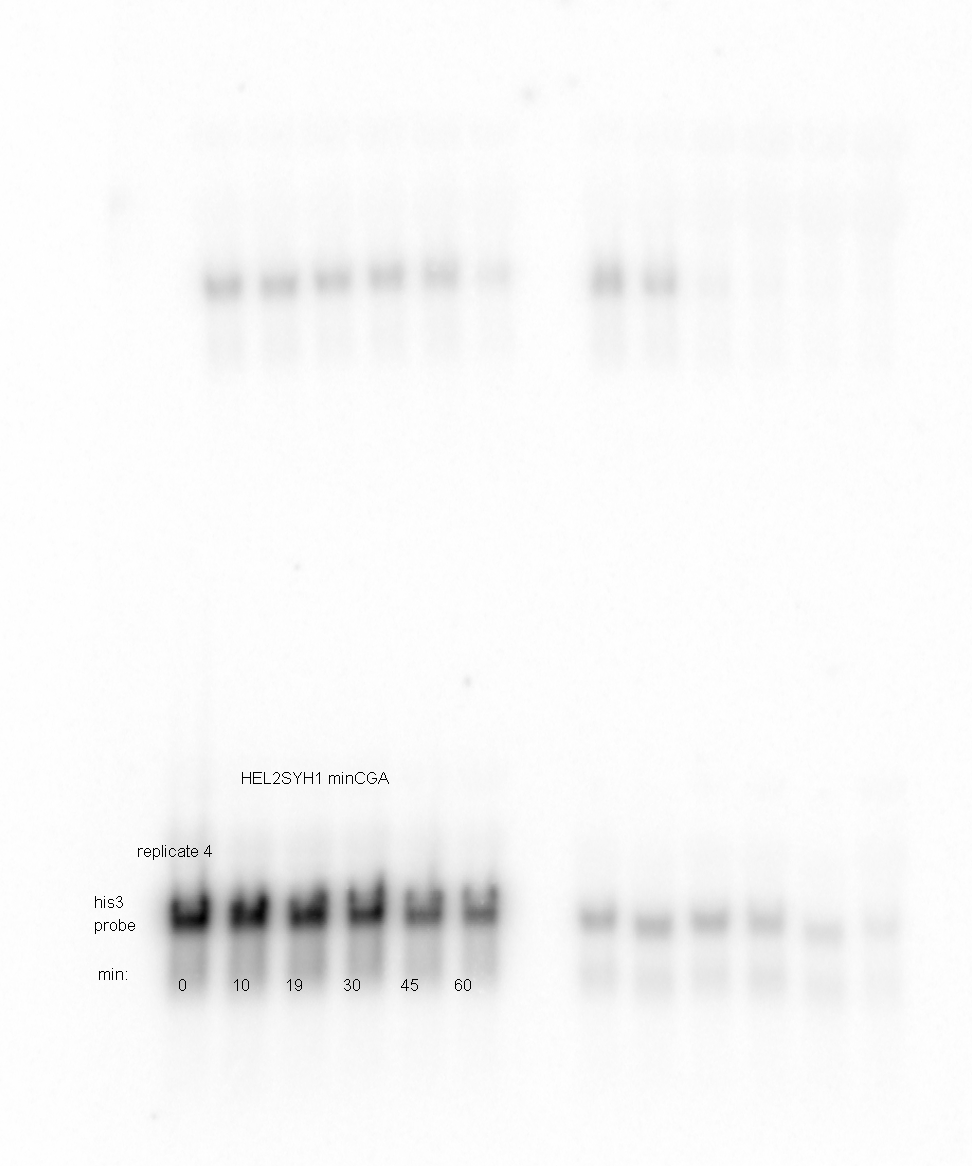

Supplement: Figure 4—source data 5. [file elife-76038-fig4-data5.zip › HEL2SYH1/minCGA/annotated_his3_rep_4.tif]

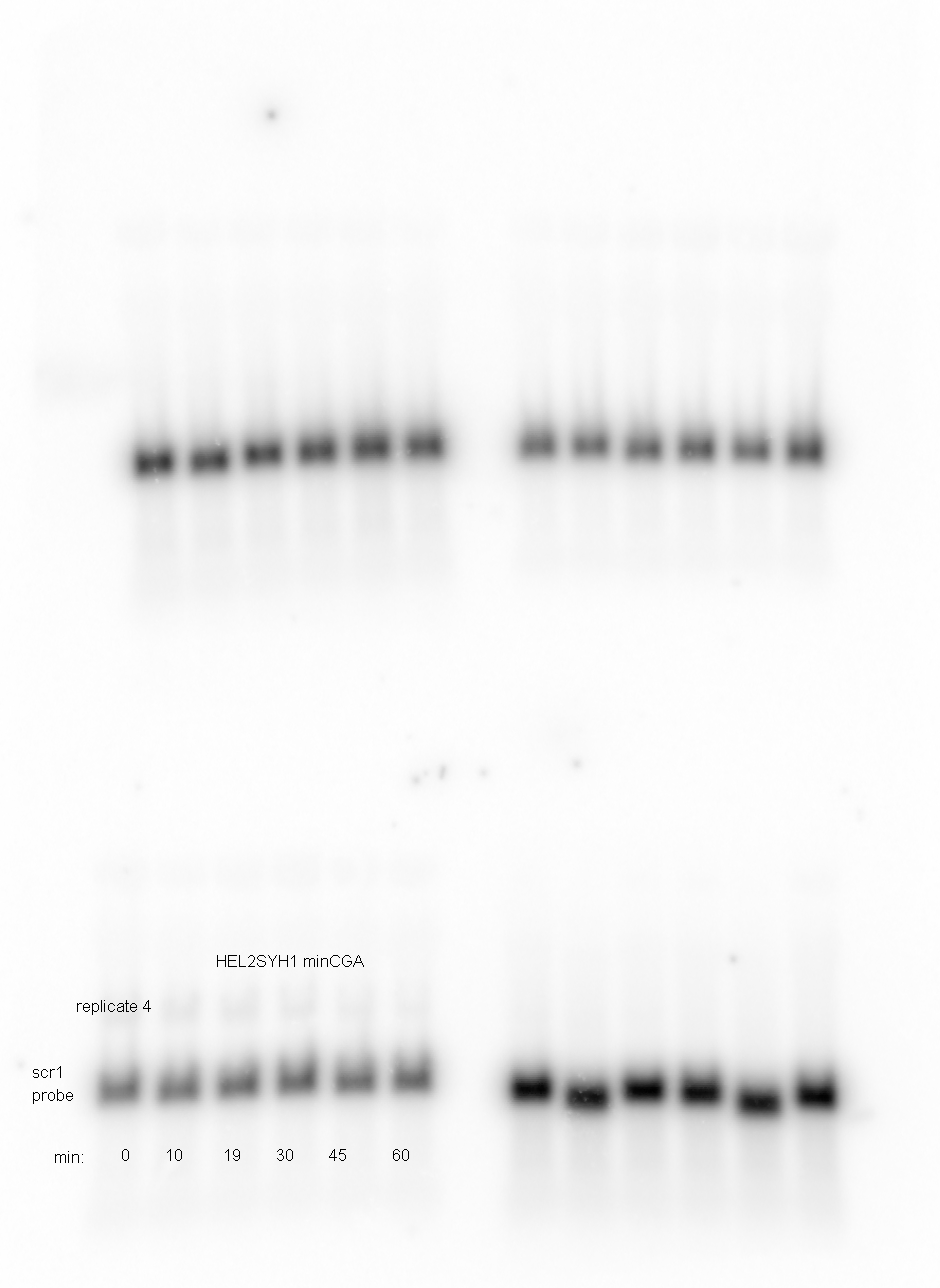

Supplement: Figure 4—source data 5. [file elife-76038-fig4-data5.zip › HEL2SYH1/minCGA/annotated_scr1_rep_4.tif]
